# FGF6 and FGF9 regulate UCP1 expression independent of brown adipogenesis

**DOI:** 10.1038/s41467-020-15055-9

**Published:** 2020-03-17

**Authors:** Farnaz Shamsi, Ruidan Xue, Tian Lian Huang, Morten Lundh, Yang Liu, Luiz O. Leiria, Matthew D. Lynes, Elena Kempf, Chih-Hao Wang, Satoru Sugimoto, Pasquale Nigro, Kathrin Landgraf, Tim Schulz, Yiming Li, Brice Emanuelli, Srinivas Kothakota, Lewis T. Williams, Niels Jessen, Steen Bønløkke Pedersen, Yvonne Böttcher, Matthias Blüher, Antje Körner, Laurie J. Goodyear, Moosa Mohammadi, C. Ronald Kahn, Yu-Hua Tseng

**Affiliations:** 1 Section on Integrative Physiology and Metabolism, Joslin Diabetes Center, Harvard Medical School, Boston, MA 02215 USA; 2 Division of Endocrinology and Metabolism, Huashan Hospital, Shanghai Medical College, Fudan University, Shanghai, China; 3 The Novo Nordisk Foundation Center for Basic Metabolic Research, University of Copenhagen, Copenhagen, Denmark; 4 Department of Biochemistry and Molecular Pharmacology, New York University School of Medicine, New York, NY 10016 USA; 5 Department of Pharmacology, Ribeirao Preto Medical School, University of São Paulo, Ribeirão Preto, Brazil; 6 Center of Research of Inflammatory Diseases, Ribeirao Preto Medical School, University of São Paulo, Ribeirão Preto, Brazil; 7 Center for Pediatric Research Leipzig, University Hospital for Children and Adolescents, University of Leipzig, Leipzig, Germany; 8 German Institute of Human Nutrition, Potsdam-Rehbrücke, Germany; 9 Five Prime Therapeutics, San Francisco, CA 94080 USA; 10 Steno Diabetes Center Aarhus, Aarhus University Hospital, 8200 Aarhus N, Denmark; 11 Department of Biomedicine, Aarhus University, 8000 Aarhus C, Denmark; 12 Department of Clinical Medicine, Aarhus University, 8200 Aarhus N, Denmark; 13 Institute of Clinical Medicine, University of Oslo, Oslo, Norway; 14 Department of Clinical Molecular Biology, Akershus Universitetssykehus, Lørenskog, Norway; 15 IFB Adiposity Diseases, University of Leipzig, Leipzig, Germany; 16 Department of Internal Medicine (Endocrinology and Nephrology), University of Leipzig, Leipzig, Germany; 17 Harvard Stem Cell Institute, Harvard University, Cambridge, MA 02138 USA

**Keywords:** Fat metabolism, Adipocytes

## Abstract

Uncoupling protein-1 (UCP1) plays a central role in energy dissipation in brown adipose tissue (BAT). Using high-throughput library screening of secreted peptides, we identify two fibroblast growth factors (FGF), FGF6 and FGF9, as potent inducers of UCP1 expression in adipocytes and preadipocytes. Surprisingly, this occurs through a mechanism independent of adipogenesis and involves FGF receptor-3 (FGFR3), prostaglandin-E2 and interaction between estrogen receptor-related alpha, flightless-1 (FLII) and leucine-rich-repeat-(in FLII)-interacting-protein-1 as a regulatory complex for UCP1 transcription. Physiologically, FGF6/9 expression in adipose is upregulated by exercise and cold in mice, and FGF9/FGFR3 expression in human neck fat is significantly associated with UCP1 expression. Loss of FGF9 impairs BAT thermogenesis. In vivo administration of FGF9 increases UCP1 expression and thermogenic capacity. Thus, FGF6 and FGF9 are adipokines that can regulate UCP1 through a transcriptional network that is dissociated from brown adipogenesis, and act to modulate systemic energy metabolism.

## Abstract (web summary)

Uncoupling protein-1 (UCP1) plays a central role in energy dissipation in brown adipose tissue. Here the authors show that FGF6 and FGF9 induce UCP1 expression in adipocytes and preadipocytes, via modulation of a transcriptional network that is dissociated from brown adipogenesis.

## Introduction

Brown adipose tissue (BAT) plays an essential role in adaptive thermogenesis, a physiological process in which energy is dissipated in response to environmental changes such as cold and diet^1^. In addition to classical BAT, brown-like multilocular beige/brite adipocytes can be recruited within white adipose tissue (WAT) upon cold exposure, treatment with β-adrenergic receptor agonists, and other stimuli^2,3^. The identification of metabolically active brown and beige/brite fat in adult humans has positioned this tissue at the center of investigations into human energy metabolism^4–7^. In addition to increased energy dissipation, activation of brown and beige/brite fat leads to enhanced glucose tolerance and insulin sensitivity in both humans and mice^8–10^. Therefore, strategies leading to increased mass or enhanced activity of BAT can potentially be utilized to combat obesity and its related metabolic disorders.

The unique thermogenic capacity of brown and beige/brite fat is attributable to the high density of mitochondria and the expression of uncoupling protein 1 (UCP1) in these thermogenic adipocytes^11,12^. UCP1 acts as a proton channel, localized to the inner mitochondrial membrane that allows protons in the mitochondrial intermembrane space to re-enter the mitochondrial matrix without generating ATP. This process dissipates energy, converting chemical energy to heat. Although some UCP1-independent thermogenic pathways have been shown to exist^13,14^, UCP1 is indispensable for acute cold adaptation. Mice deficient in UCP1 are cold sensitive^15^ and have increased susceptibility to diet-induced obesity (DIO)^16–18^. Conversely, overexpression of UCP1 in WAT protects mice from DIO^19^.

The conventional view of brown adipocyte differentiation from preadipocytes involves the activation of adipogenic/thermogenic transcriptional cascades, including peroxisome proliferator-activated receptor gamma (*Pparg*), CCAAT/enhancer binding proteins, PR domain containing 16 (*Prdm16*), and peroxisome proliferative activated receptor, gamma, coactivator 1 alpha (*Ppargc1a*)^3,20^. This activation results in the expression of genes characteristic of fully differentiated brown adipocytes, including fatty acid binding protein 4 (*Fabp4)*, deiodinase 2 (*Dio2*), cell death-inducing DNA fragmentation factor A (*Cidea*), and the brown fat defining marker *Ucp1*. During regular in vitro differentiation, UCP1 expression is restricted to the late stage of brown fat differentiation when lipid accumulation is already evident.

Given the critical function of UCP1, we design a peptide library screen to identify the factors that can induce *Ucp1* expression in a murine brown preadipocyte cell line. The screen identifies fibroblast growth factor 6 (FGF6) and FGF9 as potent inducers of *Ucp1* expression. Contrary to the classical view of brown adipogenesis, here we find that FGF6 and FGF9 can induce a high level of *Ucp1* expression in brown and white preadipocytes independent of adipogenic differentiation. Instead, FGF6- and FGF9-induced *Ucp1* expression is mediated by stimulation of prostaglandin E2 (PGE2) biosynthesis and is completely uncoupled from the conventional adipogenic or brown fat specific transcription factors. Combining CRISPR-based chromatin immunoprecipitation (ChIP) with quantitative proteomics, we discover a transcriptional regulatory complex composed of nuclear receptor estrogen related receptor, alpha (ESRRA or ERRA), transcription coactivator flightless I actin binding protein (FLII), and leucine rich repeat (in FLII) interacting protein 1 (LRRFIP1) that regulates *Ucp1* gene expression. Importantly, *Fgf6* and *Fgf9* expression is induced in adipose tissue in response to thermogenic stimuli such as cold exposure and exercise training. Loss of FGF9 in BAT impairs thermoregulation and reduces BAT thermogenic capacity. Conversely, in vivo administration of wild-type or endocrinized FGF9 enhances BAT thermogenic function. Collectively, these data establish the mechanism for the induction of UCP1 expression and thermogenic activity by FGF6/9.

## Results

### FGF6 and FGF9 induce UCP1 independently of adipogenesis

To identify factors that induce *Ucp1* expression, we performed a high-throughput screen using a peptide library containing more than 5000 mammalian secreted peptides^21^ on an immortalized murine brown preadipocyte cell line. The screen identified some paracrine members of the FGF family, the most potent of which were FGF6 and FGF9, as strong inducers of *Ucp1* expression (Supplementary Fig. 1a).

FGFs regulate a multitude of developmental processes and physiological functions^22^. In mammals, the 18 members of the FGF family are divided into two categories, endocrine and paracrine. FGF6 and FGF9 are paracrine FGFs. Apart from the recent discovery of the anti-diabetic effects of FGF1^23,24^, paracrine FGFs have not been previously implicated in metabolism.

**Fig. 1:**
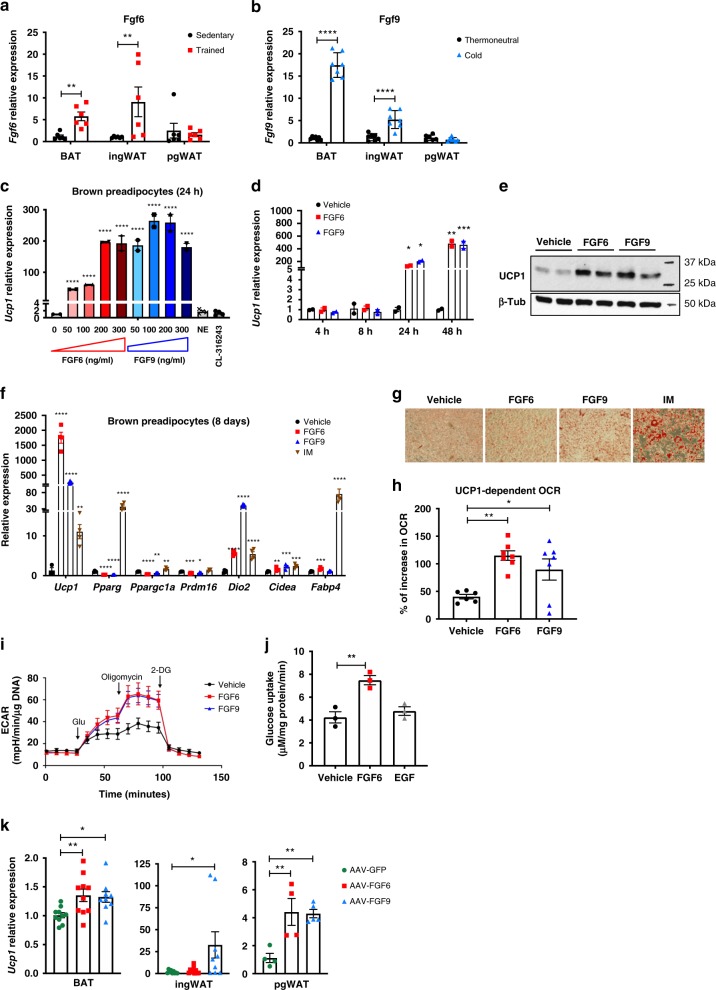
FGF6 and FGF9 induce UCP1 expression in preadipocytes independent of adipocyte differentiation. **a** *Fgf6* expression in BAT, ingWAT, and pgWAT of male C57BL6 mice (trained or sedentary). *N* = 5–6 per group. **b** *Fgf9* expression in BAT, ingWAT, and pgWAT of male C57BL6 mice exposed to 5 °C (cold) or 30 °C (thermoneutral) for 7 days. *N* = 5–7 per group. **c** *Ucp1* gene expression in brown preadipocytes in response to 24 h treatment with various concentrations of FGF6 or FGF9. *N* = 3 per group. Ct values for vehicle: 26; FGF6: 20–18; and FGF9: 17–18. **d** Time course of *Ucp1* induction in brown preadipocytes in response to FGF6 (200 ng/ml) or FGF9 (100 ng/ml) treatment. *N* = 3 per group. **e** UCP1 protein level in brown preadipocytes treated with vehicle, FGF6 (200 ng/ml), or FGF9 (100 ng/ml) for 48 h. Twenty three micrograms of total protein was used for WB. **f** Gene expression and **g** Oil Red O staining in murine brown preadipocytes treated with vehicle, FGF6 (200 ng/ml), FGF9 (100 ng/ml), or adipogenic induction/differentiation cocktail for 8 days. Scale bar = 200 μm. *N* = 2–3 per group. Ct values for vehicle: 31–32; FGF6: 21–22; FGF9: 24–25; and IM: 28–29. **h** Change in oxygen consumption rate (OCR) upon stimulation with the UCP1-specific activator PFOA (600 μM) in brown preadipocytes treated with vehicle, FGF6 (200 ng/ml), or FGF9 (100 ng/ml) for 48 h. *N* = 6–7 per group. **i** Glycolysis stress test in brown preadipocytes treated with vehicle, FGF6 (200 ng/ml), or FGF9 (100 ng/ml) for 24 h. *N* = 6–7 per group. **j** Glucose uptake in brown preadipocytes treated with vehicle, FGF6 (200 ng/ml), or EGF for 24 h. *N* = 3 per group. **k** *Ucp1* expression in BAT, ingWAT, and pgWAT depot of mice injected with AAV-GFP, AAV-FGF6, or AAV-FGF9. *N* = 4–5 per group. Data are presented as means ± SEM. One-way ANOVA in **c**, **h**, **j**, and **k**. Two-way ANOVA in **a**, **b**, **d**, and **f**. ****p < 0.0001, ***p < 0.001, **p < 0.01, and *p < 0.05. A representative from a total of two to three independent experiments is shown. Source data are provided as a Source Data file.

We sought to determine whether FGF6 and FGF9 could be induced in response to cold exposure, the classical stimulus that activates brown and beige/brite adipose tissue for thermogenesis^25^. In addition to cold, exercise training also induces UCP1 expression and browning of subcutaneous inguinal WAT (ingWAT)^26,27^. Notably, we found that in both BAT and ingWAT, *Fgf6* expression was induced by exercise training (Fig. 1a). Consistent with a previous report^28^, *Fgf9* expression was increased in these fat depots of cold-exposed mice (Fig. 1b). High-fat feeding also increased *Fgf9* expression in BAT (Supplementary Fig. 1b). Cold exposure also increased *Fgf9* expression in BAT of DIO mice (Supplementary Fig. 1b). *Fgf9* expression in BAT was dependent on sympathetic innervation since it was significantly reduced after BAT denervation (Supplementary Fig. 1c). These data suggest that FGF6/9 may play a role in cold-, diet-, or exercise-induced thermogenic programs and prompted us to perform in-depth studies to investigate the underlying mechanisms.

Treatment of murine brown preadipocytes with different concentrations of recombinant FGFs for 24 h verified the screen results and demonstrated that FGF6 and FGF9 could robustly induce *Ucp1* expression in brown preadipocytes in a dose-dependent manner (Fig. 1c and Supplementary Fig. 1d). By comparison, conventional stimuli of *Ucp1*, such as norepinephrine (NE) or the β3 adrenoceptor agonist, CL-316,243, were unable to induce *Ucp1* in preadipocytes (Fig. 1c). As early as 24 h after treatment, we observed significant induction of *Ucp1* messenger RNA (mRNA) by FGF6 and FGF9 (Fig. 1d and Supplementary Fig. 1e), and the levels continued to rise at 48 h when FGF-induced UCP1 protein expression was also evident in brown preadipocytes (Fig. 1e).

To determine whether FGF6- and FGF9-induced UCP1 expression was due to adipogenic differentiation, brown preadipocytes were treated with FGF6, FGF9, or adipogenic induction medium (IM) for 8 days (Supplementary Fig. 1f). Cells treated with FGF6 and FGF9 in growth medium expressed 112- and 17-fold higher levels of *Ucp1* mRNA, respectively, compared to the cells differentiated with the conventional IM (Fig. 1f). This occurred despite the fact that the FGF treatment did not induce the expression of other key adipocyte genes such as *Pparg* and *Fabp4*, or the transcriptional regulators that are specific to brown adipocytes such as *Ppargc1a* and *Prdm16* (Fig. 1f). Treatment of brown preadipocytes with FGF6 or FGF9 did, however, significantly increase the expression of two other markers associated with thermogenic adipocytes, *Dio2* and *Cidea*. Consistent with our gene expression data, FGF-treated cells did not display an adipocyte-like morphology and had minimal lipid accumulation compared to cells differentiated by treatment with IM (Fig. 1f, g).

To examine the functional effects of increased UCP1 expression on mitochondrial activity in brown preadipocytes, we measured UCP1-dependent respiration using a specific UCP1 activator, perfluorooctanoic acid (PFOA)^29^. FGF6- or FGF9-treated brown preadipocytes consumed more oxygen in response to PFOA stimulation (Fig. 1h), demonstrating that the UCP1 protein induced by FGFs was indeed functional. Additionally, FGF6 and FGF9 treatment profoundly increased the glycolytic capacity of brown preadipocytes measured by the glycolysis stress test (Fig. 1i). FGF6 treatment also increased glucose uptake (Fig. 1j). This increase in UCP1 and glucose uptake was independent of the growth factor activity of FGF6, because epidermal growth factor (EGF), another potent growth factor and mitogen, was unable to enhance glucose uptake in these cells (Fig. 1j).

We found that FGF6 and FGF9 exerted similar effects in several progenitor cell types with adipogenic potential, including immortalized murine white preadipocytes, primary stromal-vascular fraction (SVF) isolated from BAT and ingWAT of mice, C3H10T1/2 mouse multipotent mesenchymal cells, and mouse embryonic fibroblast (MEF). However, we found no effect in C2C12 mouse myoblast cells (Supplementary Fig. 1g–l). FGF6 and FGF9 also enhanced UCP1 expression in mature murine brown and white adipocytes without increasing the expression of other general or BAT-selective adipocyte markers (Supplementary Fig. 2). When added to adipogenic induction media, FGF6 and FGF9 suppressed adipocyte differentiation (Supplementary Fig. 3). Importantly, FGF6 and FGF9 could also program human white and brown adipose precursors to acquire greater thermogenic capacity upon differentiation (Supplementary Fig. 4).

To determine whether FGF6 and FGF9 are able to induce UCP1 in adipose tissue in vivo, we used adeno-associated virus 2/8 (AAV2/8)-mediated gene transfer to overexpress FGF6, FGF9, or green fluorescent protein (GFP) under the control of the human adiponectin promoter. Compared to the control mice receiving AAV-GFP, mice receiving AAV-FGF9 displayed significantly higher levels of *Ucp1* expression in BAT, ingWAT, and perigonadal WAT (pgWAT), while FGF6 overexpression increased *Ucp1* expression in BAT and pgWAT, but not ingWAT (Fig. 1k and Supplementary Fig. 5). Moreover, using a Ucp1-cre-Rosa26-Luciferase reporter mouse model, we confirmed that FGF9 overexpression activated *Ucp1* promoter in ingWAT, resulting in Luciferase expression (Supplementary Fig. 6a). Additionally, histological analysis of ingWAT showed the presence of UCP1-expressing multilocular cells in mice receiving AAV-FGF9 (Supplementary Fig. 6b).

### FGF6 and FGF9 induce UCP1 expression by activating FGFR3

**Fig. 2:**
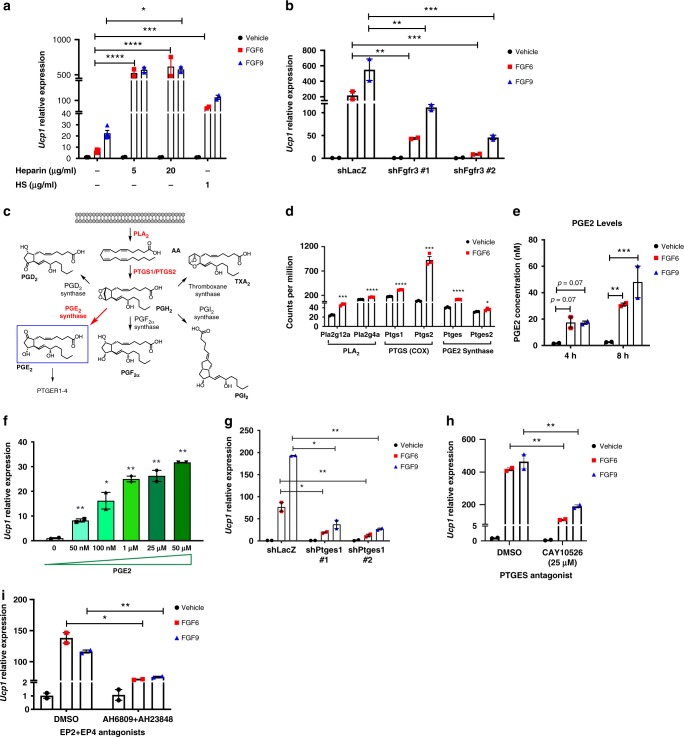
FGF6 and FGF9 induce UCP1 expression by promoting prostaglandin E2 biosynthesis. **a** FGF6/9-induced *Ucp1* expression in the presence or absence of heparin or HS. *N* = 3 per group. **b** *Ucp1* expression in control (shLacZ) and *Fgfr3* knockdown cell lines upon 24 h treatment with vehicle, FGF6, or FGF9. *N* = 3 per group. **c** Prostaglandin biosynthesis pathway. Genes encoding the enzymes marked in red are upregulated by FGF6 treatment. **d** Counts per millions for transcripts encoding PGE2 biosynthesis enzymes from RNA-sequencing of brown preadipocytes treated with FGF6 or vehicle for 24 h. **e** PGE2 concentration in the culture media of brown preadipocytes upon treatment with vehicle, FGF6, or FGF9. *N* = 3 per group. **f** *Ucp1* expression in brown preadipocytes treated with PGE2 for 24 h. *N* = 2 per group. **g** *Ucp1* expression in control (shLacZ) and *Ptges* knockdown cells treated with vehicle, FGF6, or FGF9 for 24 h. *N* = 3 per group. **h** *Ucp1* expression in white preadipocytes treated with vehicle, FGF6, or FGF9 in the presence of PTGES-selective inhibitor (CAY10526) or equimolar concentration of DMSO. *N* = 3 per group. **i** *Ucp1* expression in white preadipocytes treated with vehicle, FGF6, or FGF9 in the presence of both EP2 and EP4 receptor antagonists (AH6809 + AH23848) or equimolar concentration of DMSO. *N* = 3 per group. FGF6 and FGF9 were used at concentrations of 200 and 100 ng/ml, respectively. Data are presented as means ± SEM. Two-way ANOVA. ****p < 0.0001, ***p < 0.001, **p < 0.01, and *p < 0.05. A representative from a total of two to three independent experiments is shown. Source data are provided as a Source Data file.

Paracrine FGFs have limited spatial diffusion in the pericellular space due to their high affinities for heparin/heparan sulfate (HS) proteoglycans (*HSPGs*). The interaction of HSPG with paracrine FGFs and FGF receptors (FGFR) also facilitates and stabilizes the FGF–FGFR interactions^30^. Accordingly, we found that the addition of either heparin or HS augmented FGF6- and FGF9-mediated *Ucp1* induction (Fig. 2a). The ability of FGF6 and FGF9 to induce *Ucp1* expression was completely blocked in the presence of a pan-FGFR antagonist, PD-173074^31^ (Supplementary Fig. 7a). To identify specific FGFR(s) involved in FGF6/9-induced *Ucp1* expression, we systematically knocked down each individual *Fgfr* in mouse brown preadipocytes. Knockdown of *Fgfr3*, but not *Fgfr1*, *2*, or *4*, significantly reduced the ability of both FGFs to induce *Ucp1* expression (Fig. 2b and Supplementary Fig. 7b–f), demonstrating that the effect of these FGFs on *Ucp1* expression is mediated by FGFR3.

### FGF6 and FGF9 induce UCP1 by promoting PGE2 biosynthesis

To identify molecular pathways that mediate the effect of FGFs on UCP1 induction, we performed RNA-sequencing (RNA-seq) on mouse brown preadipocytes treated with FGF6 for 4, 8, or 24 h (Supplementary Data 1). Pathway enrichment analysis revealed that FGF6 upregulated expression of genes involved in cell growth and proliferation, cell–cell and cell–ECM interactions, and arachidonic acid metabolism (Supplementary Fig. 8a).

Arachidonic acid metabolism involves oxidation and transformation of arachidonic acid to families of related metabolites, such as PGEs, leukotrienes, and thromboxanes^32^. We found transcripts encoding enzymes involved in PGE2 biosynthesis to be coordinately upregulated in the FGF6-treated preadipocytes (Fig. 2c). RNA-seq data showed that FGF6 significantly upregulated the expression of transcripts encoding the enzymes in the first (*Pla2g12aA* and *Pla2g4a*), second (*Ptgs1* and *Ptgs2*), and third (*Ptges* and *Ptges2*) steps of PGE2 biosynthesis (Fig. 2d). Similarly, levels of *Ptgs2* and *Ptges* mRNA and protein were markedly induced by FGF9 (Supplementary Fig. 8b–d). Consistent with these findings, elevated levels of PGE2 were detected in culture media of FGF6- or FGF9-treated brown and white preadipocytes (Fig. 2e and Supplementary Fig. 8e). Exogenously added PGE2 also induced *Ucp1* expression in preadipocytes in a dose-dependent manner (Fig. 2f), suggesting that elevated PGE2 biosynthesis is an intermediate event leading to *Ucp1* expression in response to FGF6 or FGF9. To test this hypothesis, we knocked down *Ptges* in brown preadipocytes using two independent *Ptges* short hairpin RNAs (shRNAs) and found a marked reduction in FGF6- and FGF9-induced *Ucp1* expression (Fig. 2g and Supplementary Fig. 8f), indicating that PGE2 biosynthesis is essential for FGF6/9-induced UCP1 expression. Additionally, using three structurally different small molecules that selectively inhibit either PTGES expression (CAY10526) or activity (CAY10678 and PF-9184), we further verified that PTGES expression and enzymatic activity are indispensable for FGF6/9-mediated UCP1 expression (Fig. 2h and Supplementary Fig. 8g, h).

PGE2 acts through four types of G protein-coupled receptors (PTGER1–4 or EP1–4) to activate different downstream signaling pathways^33^. Only dual inhibition of both EP2 and EP4 receptors blunted the response of cells to FGF6 and FGF9 (Fig. 2i and Supplementary Fig. 8i–l), demonstrating that PGE2 can act through both EP2 and EP4 to induce *Ucp1* expression in preadipocytes.

### ERRA and FLII-LRRFIP1 complex regulate UCP1 expression

**Fig. 3:**
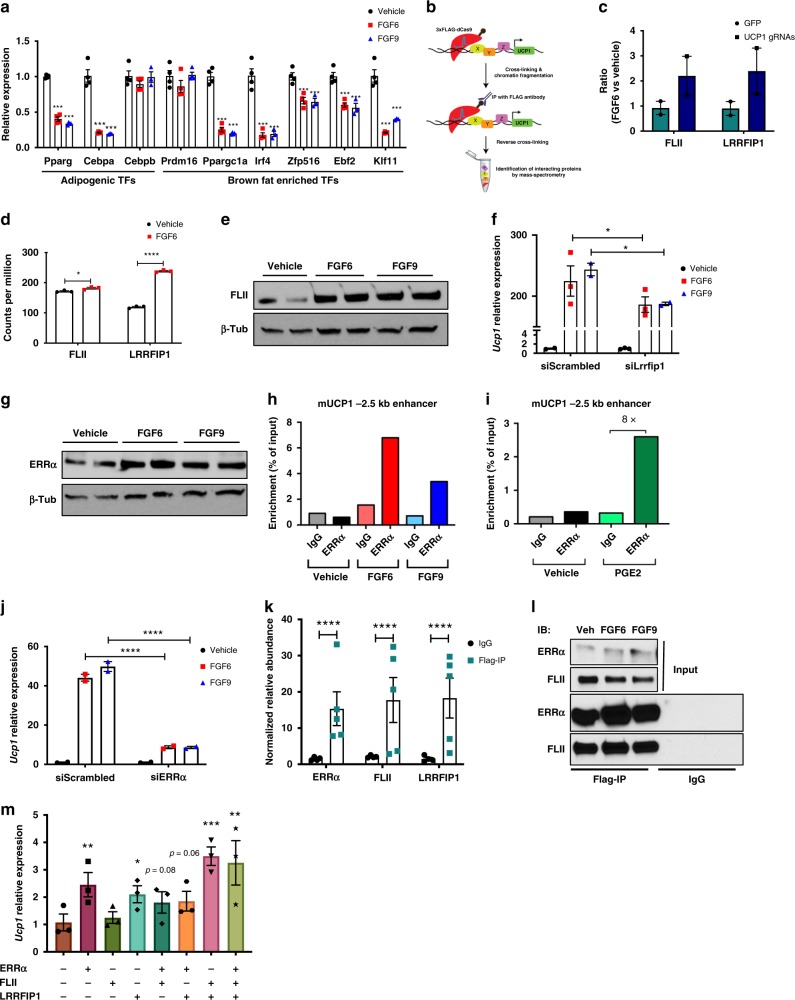
Recruitment of ERRA-FLII-LRRFIP1 complex induces UCP1 expression. **a** Gene expression in brown preadipocytes treated with vehicle, FGF6, or FGF9 for 24 h. **b** Schematic presentation of enChIP experiment. **c** Relative enrichment of FLII and LRFFIP1 proteins in the dCas9-FLAG IP in cells transfected with *Ucp1* gRNAs and GFP control. *N* = 2 per group. **d** Counts per million for *Flii* and *Lrrfip1* transcripts in brown preadipocytes treated with vehicle or FGF6 for 8 h. *N* = 3 per group. **e** FLII protein level in brown preadipocytes treated with vehicle, FGF6, or FGF9 for 48 h. *N* = 2 per group. Thirty micrograms of total protein was used for WB. **f** *Ucp1* expression in brown preadipocytes transfected with scrambled siRNA or si*Lrrfip1*, followed by treatment with vehicle, FGF6, or FGF9 for 24 h. *N* = 3 per group. **g** ERRA protein level in brown preadipocytes treated with vehicle, FGF6, or FGF9 for 72 h. *N* = 2 per group. Thirty micrograms total protein was used for WB. **h** Chromatin immunoprecipitation of ERRA on the *Ucp1* enhancer in brown preadipocytes treated with vehicle, FGF6, or FGF9 for 48 h. **i** Chromatin immunoprecipitation of ERRA on the *Ucp1* enhancer in white preadipocytes treated with vehicle (DMSO) or PGE2 for 24 h. **j** *Ucp1* expression in brown preadipocytes transfected with scrambled siRNA or si*Erra*, followed by treatment with vehicle, FGF6, or FGF9 for 24 h. *N* = 3 per group. **k** Relative abundance of the indicated proteins detected by mass spectrometry in FLAG IP and IgG samples. *N* = 4–5. **l** Western blot for ERRA and FLII in FLAG IP and IgG. FGF6 and FGF9 were used at concentrations of 200 and 100 ng/ml, respectively. **m** *Ucp1* expression in brown preadipocytes transfected with *Erra*, *Flii*, and *Lrrfip1* cDNA. Data are presented as means ± SEM. Two-way ANOVA. ****p < 0.0001, ***p < 0.001, **p < 0.01, and *p < 0.05. A representative from a total of two to three independent experiments is shown. Source data are provided as a Source Data file.

One of the most striking findings regarding the effect of FGF6/9 on UCP1 induction in preadipocytes was that it occurred independently of adipocyte differentiation. Most of the key adipogenic or BAT-enriched transcription factors were unchanged or even downregulated by FGF6 or FGF9 treatment (Fig. 3a and Supplementary Fig. 9a). Additionally, the effect of FGF6/9 on UCP1 expression was not reduced in cells lacking PPARG (Supplementary Fig. 9b). This was an unexpected observation because the currently known transcriptional regulators of *Ucp1* act through PPARG, CEBPB, PPARGC1A, or PRDM16^34^, and suggested that FGF6/9 may utilize a non-canonical transcriptional regulatory mechanism to induce *Ucp1* expression.

To identify factors recruited to the *Ucp1* *cis*-regulatory region in response to FGF6/9 stimulation, we used an unbiased CRISPR-based technique termed enChIP (engineered DNA-binding molecule-mediated ChIP)^35^. The *Ucp1* promoter and enhancer regions were targeted using a FLAG-tagged, catalytically inactive form of Cas9 (dCas9) and five guide RNAs, which recognized up to 3 kb upstream of the *Ucp1* transcription start site (TSS) (Fig. 3b and Supplementary Fig. 10). By immunoprecipitating with an anti-FLAG antibody, we isolated proteins associated with the *Ucp1* regulatory region and subjected them to tandem mass tag (TMT) labeling followed by mass spectrometry. Among the top proteins identified by this approach (Supplementary Data 2), we found that nuclear receptor coactivator FLII and its binding partner, LRRFIP1, were enriched at the *Ucp1* regulatory region upon FGF6 treatment (Fig. 3c). Consistent with the potential role of these factors in FGF6/9-induced *Ucp1* transcription, treatment with FGF6/9 upregulated both *Flii* and *Lrrfip1* expression in brown preadipocytes (Fig. 3d, e). While knockdown of *Flii* alone did not affect *Ucp1* expression (Supplementary Fig. 11a, b), knocking down *Lrrfip1* significantly impaired FGF6/9-induced *Ucp1* expression (Fig. 3f and Supplementary Fig. 11c).

FLII is a transcriptional coactivator that regulates the expression of target genes through interaction with DNA-binding nuclear receptors^36^. We, therefore, hypothesized that recruitment of FLII/LRRFIP1 to the *Ucp1* regulatory region is mediated by a nuclear receptor that binds to the *Ucp1* regulatory region upon FGF6/9 treatment. Several nuclear receptors are known to positively or negatively regulate *Ucp1* expression^37,38^. RNA-seq of brown preadipocytes treated with FGF6 revealed that the expression of *Erra* was significantly induced at 4 and 8 h after FGF treatment, while the expression of many other nuclear receptors were either unchanged or downregulated (Supplementary Fig. 11d). Induction of ERRA by FGFs was also observed at the protein level (Fig. 3g).

Next, we performed ChIP using an anti-ERRA antibody and found that in brown preadipocytes, the binding of ERRA to the −2.5 kb *Ucp1* enhancer was substantially enhanced by treatment with FGF6 or FGF9 (Fig. 3h). Binding of ERRA was specific to this enhancer region, and it was not detected in the −15 kb upstream control region (Supplementary Fig. 11e). Although we did not detect the binding of ERRA to the *Ucp1* locus using enChIP, presumably due to the low abundance of ERRA in preadipocytes, data from direct ChIP demonstrated the direct binding of ERRA to the UCP1 enhancer region in FGF6/9-treated cells. Consistent with the ability of PGE2 to induce *Ucp1* expression, PGE2 treatment also robustly stimulated ERRA binding to the *Ucp1* enhancer (Fig. 3i). Furthermore, knocking down *Erra* significantly impaired FGF6/9-induced *Ucp1*, but not *Ptgs2* and *Ptges* expression (Fig. 3j and Supplementary Fig. 11f–h), demonstrating that ERRA is required for FGF6/9-induced *Ucp1* expression and that it acts downstream of PGE2.

ERRA functions in concert with other transcriptional activators, such as PPARGC1A, to regulate the expression of *Ucp1* and genes involved in mitochondrial biogenesis^39,40^. We found that expression of PPARGC1A was significantly downregulated in FGF6- and FGF9-treated cells (Fig. 3a), and that FGF6/9-induced *Ucp1* expression was unaltered in Ppargc1a-knockout (KO) brown preadipocytes (Supplementary Fig. 11i). These data demonstrate that PPARGC1A does not play a role in the regulation of *Ucp1* expression by FGF6 or FGF9.

Based on our enChIP and RNA-seq data, we hypothesized that the FLII/LRRFIP1 complex could act as ERRA co-activators. To test this hypothesis, we examined whether these proteins could physically interact with each other. We overexpressed FLAG-tagged ERRA in brown preadipocytes, immunoprecipitated proteins associated with ERRA, and then subjected the immunoprecipitate to TMT-based mass spectrometry. Intriguingly, both FLII and LRRFIP1 were found to interact with ERRA (Fig. 3k). Immunoblotting the anti-FLAG-immunoprecipitate with anti-FLII antibody further validated the interaction between ERRA and FLII (Fig. 3l).

To determine the interdependency of these factors in the regulation of UCP1 expression, we overexpressed ERRA, FLII, and LRRFIP1 either alone or in combination in murine brown preadipocytes. Overexpression of ERRA alone or together with FLII, LRRFIP1, or both induced *Ucp1* expression (Fig. 3m). Interestingly, although FLII was unable to induce *Ucp1* expression on its own, co-expression of FLII and LRRFIP1 resulted in significant upregulation of *Ucp1*, supporting the notion that FLII and LRRFIP1 cooperate in the activation of *Ucp1* transcription.

Transcriptional co-activators function as adaptors to facilitate the recruitment of the transcriptional machinery, and/or chromatin remodeling enzymes to the promoter/enhancer regions of target genes^41^. We hypothesized that FGF6/9 would promote *Ucp1* gene transcription by either establishing permissive or removing suppressive histone modifications. H3K4me3 is known to be strongly associated with the TSS of actively transcribed genes. Indeed, FGF6/9 increased the abundance of this active mark on the *Ucp1* TSS (Supplementary Fig. 12a). H3K27ac is associated with higher chromatin accessibility and marks active enhancers. We also observed higher levels of H3K27ac around the *Ucp1* enhancer in FGF6/9-treated preadipocytes (Supplementary Fig. 12b). H3K9me2, on the other hand, is a marker of transcriptional suppression. Consistently, we found a profound reduction in H3K9me2 positioned at the *Ucp1* TSS, proximal promoter, and enhancer (Supplementary Fig. 12c). Such changes in local chromatin accessibility could allow initiation of transcription in the absence of other known adipogenic and thermogenic transcription factors.

### Cold/exercise triggers the FGF6/9-PGE2-ERRA-FLII-UCP1 pathway

**Fig. 4:**
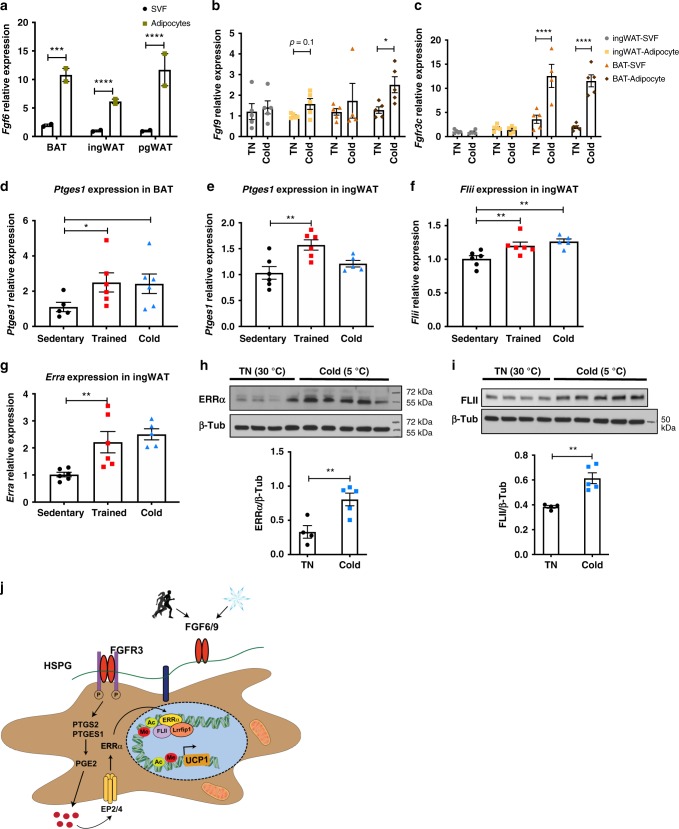
Cold and exercise activate the FGF6/9-PGE2-ERRA-FLII-UCP1 regulatory pathway in BAT and ingWAT. **a** *Fgf6* expression in SVF and adipocytes from BAT, ingWAT, and pgWAT, **b** *Fgf9*, and **c** *Fgfr3* expression in SVF and adipocytes from BAT and ingWAT of male mice housed at either 5 °C (cold) or 30 °C (TN) for 7 days. *N* = 4–5 per group. **d** *Ptges* expression in BAT and **e** ingWAT of mice either placed in cages containing running wheel for 11 days (Trained) or housed at 5 °C temperature for 7 days (cold) compared to the control group housed at room temperature (Sedentary). *N* = 5–6 per group. **f** *Flii* and **g** *Erra* expression in ingWAT (right) of male C57BL6 mice either placed in cages containing running wheel for 11 days (Trained) or housed at 5 °C temperature for 7 days (cold) compared to the control group housed at room temperature (Sedentary). *N* = 5–6 per group. **h** Top: ERRA protein level in ingWAT of male C57BL6 mice housed at either 5 °C (cold) or 30 °C (TN) for 7 days. Down: quantification of ERRA protein relative to β-tubulin. *N* = 4–5 per group. **i** Top: FLII protein level in BAT of male C57BL6 mice housed at either 5 °C (cold) or 30 °C (TN) for 7 days. Down: quantification of FLII protein relative to β-tubulin. *N* = 4–5 per group. Two-sample *t* test. **j** Proposed model for mechanism of FGF6/FGF9-mediated *Ucp1* expression. Sixty micrograms of total protein was used for WBs in **h**, **i**. Data are presented as means ± SEM. Two-way ANOVA in **a**–**c**, one-way ANOVA in **d**–**g**, and two-sample *t* test in **h**, **i**. ****p < 0.0001, ***p < 0.001, **p < 0.01, and *p < 0.05. A representative from a total of two to three independent experiments is shown. Source data are provided as a Source Data file.

Cold and exercise are two well-recognized stimuli that activate UCP1 expression in brown or beige/brite fat^11,42^. As shown above, we identified FGF6 and FGF9 as exercise- and cold-induced adipokines, respectively (Fig. 1a, b). To establish the source and target of these adipokines within the adipose microenvironment, we isolated adipocytes and SVF from BAT, ingWAT, and pgWAT of mice housed at thermoneutral (30 °C) or cold (5 °C) for 7 days. Expression levels of *Fgf6* were significantly higher in the adipocytes than in the SVF in all these fat depots (Fig. 4a). In contrast, *Fgf9* was expressed at a similar level in both adipocytes and SVF of BAT and ingWAT. However, cold exposure increased *Fgf9* expression only in adipocytes (Fig. 4b). *Fgfr3*, the receptor mediating the effect of FGF6/9 on UCP1 expression (Fig. 2b), was expressed at a higher level in BAT compared with ingWAT. Importantly, cold exposure markedly increased *Fgfr3* expression in both adipocytes and SVF cells of BAT (Fig. 4c). The fact that FGFR3 was expressed in both SVF and adipocytes suggests that the in vivo effect of these FGFs could be mediated by cells present in both fractions. These results demonstrated a coordinated regulation of the ligand (FGF9) and its receptor (FGFR3) upon cold stimulation within the adipose niche, which facilitates the paracrine action of this adipokine.

Consistent with the notion that PGE2 is a meditator of FGF6/9-induced UCP1 expression, we found elevated levels of *Ptges* expression in BAT upon cold exposure and exercise training, and in ingWAT only following exercise training (Fig. 4d, e). These data suggest that the FGF6/9-PGE2-UCP1 regulatory pathway exists in physiological conditions that enhance thermogenesis. PGE biosynthesis pathway has previously been implicated in the cold-induced browning of WAT^43,44^. However, the upstream factors regulating PGE synthesis within adipose tissue and the downstream pathways mediating its effect on thermogenic gene expression have not been elucidated. Based on our data, FGF6 and FGF9 function as exercise- and cold-induced adipokines that activate PGE2 biosynthesis to turn on the thermogenic program in both BATs and WATs. Additionally, both cold exposure and exercise training induced *Flii* and *Erra* expression in ingWAT of mice (Fig. 4f, g). ERRA and FLII protein levels were also elevated in ingWAT and BAT of cold-acclimated mice, respectively (Fig. 4h, i). Taken together, these results uncover the signaling and transcriptional cascade that mediates FGF6- and FGF9-induced UCP1 expression (Fig. 4j).

### FGF9 plays a key role in BAT thermogenesis

**Fig. 5:**
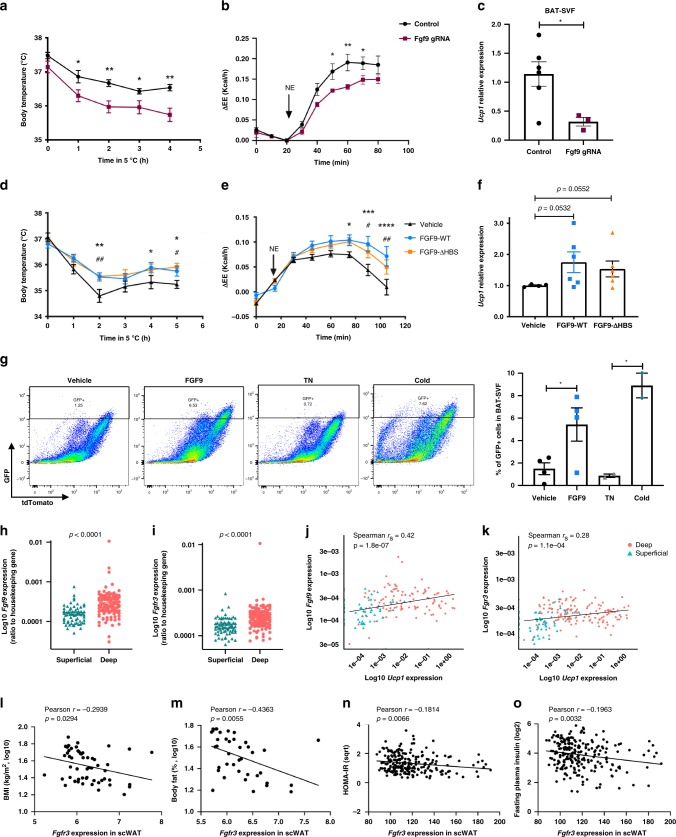
FGF9 function is crucial for BAT thermogenesis. **a** Core body temperature in adiponectin Cre-Cas9 knock-in mice injected with AAV-GFP or AAV-FGF9 gRNA. *N* = 10 per group. **b** Heat production measured by indirect calorimetry stimulated with NE (1 mg/kg) injection in Ucp1-cre-Cas9 knock-in mice injected with AAV-GFP or AAV-FGF9 gRNA. *N* = 3–6 per group. **c** *Ucp1* expression in BAT-SVF isolated from in Ucp1-cre-Cas9 knock-in mice injected with AAV-GFP or AAV-FGF9 gRNA following 3 days cold exposure (5 °C). *N* = 3–6 per group. **d** Core body temperature in mice during 5 h exposure to 5 °C cold after 10 days daily subcutaneous injection of FGF9-WT, FGF9-ΔHBS, or vehicle. *N* = 12–13 per group. **e** Heat production measured by indirect calorimetry stimulated with NE (1 mg/kg) injection after 14 days daily subcutaneous injection of FGF9-WT, FGF9-ΔHBS, or vehicle. *N* = 5–6 per group. **f** *Ucp1* expression in BAT after 17 days of daily subcutaneous injection of FGF9-WT, FGF9-ΔHBS, or vehicle. *N* = 4–6 per group. **g** Left: representative flow cytometry analysis of BAT-SVF derived from Ucp1-cre-Rosa26-mTmG mice treated with FGF9-WT or Vehicle for 7 days. Right: quantification of GFP positive cells in BAT-SVF. FGF9-WT and FGF9-ΔHBS were used at dose of 0.5 mg/kg per day. **h** *Fgf9* and **i** *Fgfr3* expression in human adipose tissue collected from deep and superficial neck fat. **j** Correlation between *Fgf9* (*n* = 162) and **k** *Fgfr3* (*n* = 196) expression and *Ucp1* expression in human adipose tissue collected from deep and superficial neck fat. Spearman’s rank-order correlation coefficient and *p* values are shown. **l** Correlation between *Fgfr3* expression in scWAT and BMI (*n* = 55) and **m** %body fat (*n* = 39) in adult cohort. **n** Correlation between *Fgfr3* expression in scWAT and HOMA-IR (*n* = 223) and **o** fasting plasma insulin (*n* = 223) in childhood cohort. Pearson’s correlation coefficient and *p* values are shown. Data are presented as means ± SEM. Two-way repeated-measure (RM) ANOVA in **a**, **b**, **d**, **e**. Two-sample *t* test in **c**, **g**. Brown–Forsythe and Welch ANOVA test in **f**. Mann–Whitney test in **h**, **i**. **p < 0.01, *p < 0.05 (FGF9-WT vs. Vehicle), ^##^*p* < 0.01, ^#^*p* < 0.05 ^(^FGF9-ΔHBS vs. Vehicle). A representative from a total of two to three independent experiments is shown. Source data are provided as a Source Data file.

To interrogate the role of FGF9 on BAT thermogenic capacity, we used a conditional Cas9 knock-in mouse strain and AAV-mediated FGF9 guide RNA (gRNA) delivery to generate a BAT-specific FGF9 loss-of-function mouse model (Supplementary Fig. 13a, b). These mice exhibited impaired cold tolerance (Fig. 5a and Supplementary Fig. 13a, b), demonstrating that BAT-derived FGF9 plays a crucial role in maintaining body temperature in response to cold exposure. Additionally, loss of FGF9 in BAT significantly reduced maximal thermogenic capacity in response to NE stimulation compared to the control group (Fig. 5b). Although we could not detect a significant change in *Ucp1* expression in the whole BAT from BAT-specific FGF9-deficient mice (Supplementary Fig. 13c), there was a significant reduction of *Ucp1* and *Erra* expression in BAT-SVF in these animals (Fig. 5c and Supplementary Fig. 13d).

To explore the therapeutic potential of FGF9, we tested the effect of recombinant FGF9 protein in vivo. Because FGF9 possesses potential mitogenic activity^45^, we generated a modified FGF9 protein carrying K168Q/R173V/R177Q triple mutations in its HS-binding site (FGF9-ΔHBS). These mutations reduce the molecule’s mitogenic potential without impacting its metabolic function (Mohammadi, personal communication). Similar strategies have previously been applied for the generation of endocrinized FGF1^24,46^. We found that daily subcutaneous administration of either wild-type FGF9 (FGF9-WT) or FGF9-ΔHBS significantly improved cold tolerance when mice were exposed to 5 °C temperature (Fig. 5d). Additionally, mice receiving either FGF9-WT or FGF9-ΔHBS showed a significantly higher thermogenic capacity in response to NE stimulation compared to the vehicle-treated control group (Fig. 5e). Consistent with improved BAT thermogenic function, both FGF9-WT and FGF9-ΔHBS increased the levels of *Ucp1* mRNA in BAT (Fig. 5f). Additionally, there was a significant increase of UCP1 protein in BAT of mice treated with FGF9-ΔHBS compared to the control (Supplementary Fig. 14a). Gene expression analysis of BAT from FGF9-treated mice revealed an upregulation of *Erra*, *Flii*, and *Lrrfip1*, as well as other genes involved in BAT thermogenesis and mitochondrial activity (Supplementary Fig. 14b).

Our in vitro studies demonstrated the ability of FGF6/9 to induce UCP1 expression in non-adipocytes. UCP1 has traditionally been viewed as a marker for mature brown or beige/brite adipocytes. Thus, the induction of UCP1 in preadipocytes by these FGFs was unexpected. We therefore used a Ucp1-cre-Rosa26-mTmG reporter strain to monitor FGF9-induced UCP1 in adipocyte progenitors in vivo. Indeed, flow cytometry analyses showed that treatment with FGF9 protein significantly induced UCP1 expression in SVFs, which resulted in cre recombination and consequently induced GFP expression in a population of cells in the BAT-SVF (Fig. 5g and Supplementary Fig. 15). Consistent with these findings, cold exposure, which markedly induced FGF9 expression in BAT (Fig. 1b), also led to a similar increase in the number of GFP+ cells in BAT-SVF (Fig. 5g). Flow cytometry analysis using Sca-1 as a marker of adipocyte progenitors^47,48^ revealed the emergence of GFP+ Sca-1+ cells in BAT-SVF upon cold exposure (Supplementary Fig. 16a). The expression of two adipocyte markers, *Fasn* and *Adipoq*, was significantly lower in the Ucp1-expressing adipocyte progenitors (GFP+ Sca-1+) than mature adipocytes (GFP+ Sca-1−) (Supplementary Fig. 16b). Taken together, these data provide direct evidence that UCP1 can be induced in the adipocyte progenitors in vivo by a physiological cue, thus giving physiological relevance to FGF-induced UCP1 expression in adipose progenitors.

To address the role of the FGF6/9-FGFR3 pathway in human thermogenic function and obesity, we measured the expression of *FGF6*, *FGF9*, and *FGFR3* in human adipose tissue biopsies. While *FGF6* was undetectable in most samples, both *FGF9* and *FGFR3* were expressed at significantly higher levels in adipose tissue from deep neck, a classic location for BAT in humans, compared to WAT isolated from superficial neck fat (Fig. 5h, i). Importantly, expression of both *FGF9* and *FGFR3* was positively correlated with *UCP1* expression in human neck fat biopsies (Fig. 5j, k). To link the expression of FGF or FGFR with human obesity, insulin resistance, or diabetes, we perform correlation studies in subcutaneous adipose tissue collected from two independent cohorts. We found that *FGFR3* expression in human abdominal scWAT was negatively correlated with body mass index (BMI) and the percentage of body fat in an adult cohort that contain subjects with a wide range of BMI (Fig. 5l, m). Similar patterns of significant correlations were found in another independent childhood obesity cohort^49^ (Supplementary Fig. 17a, b). Interestingly, *FGFR3* expression in scWAT was also negatively correlated with HOMA-IR (homeostasis model assessment of insulin resistance) and fasting plasma insulin concentrations (Fig. 5n, o). The negative correlation with fasting plasma insulin remained significant even after adjustment for BMI (Pearson’s correlation *r* = −0.142, *p* = 0.030), indicating that the relationship between FGFR3 expression and insulin sensitivity could be independent of body weight. Collectively, these data suggest that the FGF9-FGFR3 pathway may play a role in human BAT thermogenesis and have potential impacts on body weight and insulin sensitivity.

## Discussion

The rising prevalence of obesity and its comorbidities is a major global health concern. Considering the formidable capacity of BAT for energy expenditure and its role in fatty acid and glucose metabolism, harnessing BAT activity holds a promising therapeutic potential for tackling obesity. The present study reveals the discovery of two adipokines, FGF6 and FGF9, in the regulation of UCP1 expression in mouse and human adipose systems. In addition to the established β3-adrenergic-induced UCP1 expression in mature brown or beige/brite adipocytes, our findings uncover a non-canonical pathway for the regulation of UCP1 expression in adipose progenitors (Supplementary Fig. 18). First, the binding of FGF6 and FGF9 to FGFR3 induces PGE2 production through induction of the key enzymes involved in its biosynthesis. In the next step, PGE2 molecules act through EP2 and EP4 cell surface receptors to promote the binding of ERRA to the ERR response element in the *Ucp1* enhancer. ERRA binding, together with the recruitment of its transcriptional co-activators FLII/LRRFIP1 to the *Ucp1* locus, enables transcriptional activation. The mechanistic studies presented here reveal that this pathway is completely independent of the canonical signaling events downstream of β3-adrenergic receptor activation by cold^50,51^. It is conceivable that the mechanisms utilized by these adipokines in vivo might be more complex than the model we propose. In particular, because FGFR3, the receptor mediating the effects of FGF6/9, is expressed in both SVF and adipocytes, the in vivo effect of these FGFs is likely to be a collective consequence of their actions in multiple cell types. Nevertheless, the coordinated regulation of FGF ligands, receptors, and downstream transcriptional by cold exposure in mouse adipose tissue, and the strong association between FGF9/FGFR3 and UCP1 expression in human neck fat highlight a significant role for these adipokines and their downstream pathways in thermoregulation and potentially in energy homeostasis in both rodents and humans.

A recent study by Sun et al.^52^ reported that FGF9 inhibits the browning of white adipocytes in vitro. Although at first glance their results seem to contradict our findings, the reduction in thermogenic gene expression in their in vitro differentiated cells is mainly caused by suppression of adipogenesis in the presence of FGF9 in the IM, a result which is also seen in our studies (Supplementary Fig. 3). Additionally, Sun et. al.^52^ have also reported a reduction in *Fgf9* expression in ingWAT by cold exposure. This is in conflict with our findings that cold strongly elevates *Fgf9* expression in BAT and ingWAT. Importantly, we have validated these results in multiple cohorts, and our findings are consistent with five other published papers and publicly available gene expression datasets, which all show an upregulation of *Fgf9* expression in BAT and ingWAT in response to cold challenge.^28,53–56^

In conclusion, this study reveals a previously unknown role for paracrine members of the FGF family in the regulation of BAT thermogenesis and metabolism. Our mechanistic studies uncover a pathway in the regulation of UCP1 expression and energy expenditure. Data from our human studies reveal a possible role of FGF9/FGFR3 in human thermogenic fat and establish a potential link between FGF9/FGFR3 axis and energy metabolism in humans. These findings add important insights into our understanding of molecular aspects of thermoregulation and will ultimately contribute to the development of therapeutic strategies to combat obesity, type 2 diabetes, and cardiovascular disease.

## Methods

### High-throughput screen

A protein library containing more than 5000 mammalian secreted peptides^21^ was used. Murine BAT preadipocytes were partially differentiated using 5 µM dexamethasone, 0.25 mM isobutylmethylxanthine (IBMX), and 0.125 mM indomethacin for 48 h. The cells were then treated with test media containing proteins in Dulbecco’s modified Earle’s medium (DMEM) with 10% fetal bovine serum (FBS) for a further 24 h. *Ucp1* mRNA induction was assayed following the 24-h treatment. *Ucp1* mRNA induction was assayed using a QuantiGene® Plex 2.0 reagent system (Panomics, Fremont, CA).

### Mice

Study approval: All experimental procedures involving animals were performed in compliance with all relevant ethical regulations applied to the use of small rodents, and with approval by the Institutional Animal Care and Use Committees (IACUC) at Joslin Diabetes Center.

C57Bl/6J mice (stock no. 000664) were purchased from The Jackson Laboratory.

For experiments involving cold exposure, mice were housed at 5 °C (cold) or 30 °C (thermoneutral) for 7 days in a controlled environmental diurnal chamber (Caron Products & Services Inc., Marietta, OH) with free access to food and water.

For experiments involving voluntary exercise training, C57BL/6J mice were placed in cages containing running wheel for 11 days and compared to the sedentary group.

### Cell culture

All cell lines used in this study were cultured in high glucose DMEM with 10% FBS. For treatment of both preadipocytes and adipocytes, FGF6 or FGF9 recombinant proteins were added in the growth media (10% FBS in high glucose DMEM).

Mouse brown and white preadipocytes were generated using SV40 T antigen and differentiated. Cells were allowed to reach confluency, and treated with induction media supplemented with 2% FBS, 20 nM insulin, 1 nM triiodothyronine (T3), 0.125 mM indomethacin, 5 µM dexamethasone, and 0.5 mM IBMX for 2 days. Then, cells were maintained in differentiation media supplemented with 2% FBS, 20 nM insulin, and 1 nM T3 for 6 more days. During differentiation, media were changed every other day^57^.

MEF cell line was derived from mouse embryos. C2C12 myoblast and C3H/10T1/2 mouse multipotent mesenchymal progenitor cell lines were purchased from ATCC.

Primary SVF cells were isolated from BAT and ingWAT of 8-week-old male C57BL6 mice. BAT and ingWAT were digested using collagenase I (1.5 mg/ml, LS004196, Worthington Biochemical Corporation), followed by centrifugation to precipitate the SVF. Isolated cells were cultured for 24 h before FGF treatment. The PPARG-KO MEFs were a generous gift from Dr. Evan Rosen’s laboratory. The PPARGC1A-KO brown preadipocytes were a generous gift from Dr. Bruce Spiegelman’s laboratory.

Human brown and white preadipocytes were cultured in growth media containing 10% FBS in DMEM/high glucose^58,59^. In FGF pretreatment experiments, cells were treated with FGF6 and FGF9 in growth media for 6 days before adipogenic induction. For adipocyte differentiation, cells were exposed to adipogenic induction media (DMEM-H with 10% FBS, 33 μM biotin, 0.5 μM human insulin, 17 μM pantothenate, 0.1 μM dexamethasone, 2 nM T3, 500 μM IBMX, and 30 μM indomethacin).

PGE2 synthase and receptor inhibitors were purchased from Cayman Chemical (Ann Arbor, MI). Cells were co-treated with each inhibitor at indicated concentrations throughout FGF treatment.

### Oil Red O staining

Cells were washed twice with phosphate-buffered saline (PBS) and fixed with 10% buffered formalin for 30 min at room temperature. Cells were then stained with a filtered Oil Red O solution (0.5% Oil Red O in isopropyl alcohol) for 2 h at room temperature, washed several times with distilled water, and then visualized.

### Bioenergetic profiling

The Seahorse mitochondrial stress test and glycolysis stress test were performed using a Seahorse Extracellular Flux Analyzer (Seahorse Bioscience Inc., North Billerica, MA). To assess mitochondrial respiration, 0.5 μM oligomycin (EMD Chemicals Inc., Gibbstown, NJ), 1 μM FCCP (carbonyl cyanide-*p*-trifluoromethoxyphenylhydrazone), and 1 μM respiratory chain inhibitor (rotenone) were added through the injection ports.

To measure UCP1-dependent respiration, 600 μM PFOA was injected following oligomycin injection.

To stimulate thermogenesis in human white adipocytes, differentiated adipocytes were treated with forskolin (10 μM) or NE (1 μM) for 4 h prior to the Seahorse experiments.

The glycolysis stress test was performed in glucose-free assay media supplemented with 1 mM Glutamax. To measure basal glycolysis rate, maximum glycolytic capacity, and non-glycolytic acidification, 10 mM glucose, 1 μM oligomycin, and 25 mM 2-deoxy-d-glucose were added, respectively.

### Glucose uptake assay

Mouse preadipocytes were starved in DMEM with high glucose containing 1% bovine serum albumin (BSA) for 2 h prior to the assay. Cells were washed with HEPES buffer and incubated with 2-deoxy-[^3^H]glucose (0.1 mM, 0.5 μCi/ml; PerkinElmer Life and Analytical Science, Waltham, MA) for 5 min. The reaction was stopped with ice-cold PBS, followed by washing the cells twice in ice-cold PBS. Cells were then lysed in 0.1% sodium dodecyl sulfate (SDS), and glucose uptake was measured in 4 ml of scintillant using a Beckman LS6500 scintillation counter (Beckman Coulter, Indianapolis, IN). Specific glucose uptake was assessed by subtracting non-specific uptake in the presence of 20 μM cytochalasin B from total uptake. The protein content was determined using the Pierce BCA Protein Assay Kit (Thermo Fisher Scientific, Waltham, MA).

### Quantitative RT-PCR

Quantitative reverse transcription-PCR (qRT-PCR) assays were performed using an ABI Prism 7900 sequence-detection system using SYBR (Roche Applied Science, Indianapolis, IN). Relative mRNA expression was calculated by the ΔCt method and the values were normalized to the expression of ARBP, TBP, or 18S ribosomal RNA (18S). Absolute quantification of *Ucp1* transcript copy number was performed using Droplet Digital™ PCR (ddPCR™) Technology (Bio-Rad) according to the manufacturer’s instructions. The sequences of primers are provided in Supplementary Data 3.

### Western blotting

Cells or tissues were lysed in RIPA buffer (Boston BioProducts Inc., Ashland, MA) supplemented with protease inhibitor cocktail (cOmplete™, Sigma-Aldrich, Dallas, TX). The following primary antibodies were used: anti-UCP1 (ab10983 for detection of mouse UCP1, 1:2000 and ab155117 for detection of human UCP1, 1:1000), anti-PPARG (ab27649, 1:200), and anti-ERRA (ab16363, 1:1000) were purchased from Abcam (Cambridge, MA). Anti-β-tubulin (2146, 1:2000), anti-FGFR1 (9740, 1:1000), anti-COX2 (12282, 1:1000), and anti-FLII (14189, 1:1000) were purchased from Cell Signaling Technologies (Beverly, MA). Primary antibodies were incubated overnight at 4 °C. HRP-coupled secondary antibodies (Cell Signaling Technologies, Beverly, MA) were used at 1:3000 dilution for 1 h at room temperature. Proteins were detected using the Amersham enhanced chemiluminescence (ECL) prime (GE healthcare, Pittsburgh, PA). All the original uncropped and unprocessed scans are provided in the Source Data file.

### Citrate synthase activity measurement

Citrate synthase activity was measured using the MitoCheck^®^ Citrate Synthase Activity Assay Kit (Caymen Chemical) according to the manufacturer’s instruction.

### Construction of AAV adiponectin-GFP, FGF6, and FGF9 plasmids

The distal enhancer (−2664 to −2507 bp) and the promoter (−540 to +77 bp) of the human adiponectin gene were cloned from genomic DNA using the following primers (backbone in lowercase and insert in upper case):

hAdipoQ-enhancer_forward: cctagatctgaattcggtacCTCTTTCCACATGACGGC,

hAdipoQ-enhancer_reverse: acatgaattcGCTGTAGCTATTGCACAAG.

hAdipoQ-promoter_forward: tagctacagcGAATTCATGTGCAAGGTC,

hAdipoQ-promoter_reverse: attccgcggactagtttttaCCCTCTGGTATGGAATCAG.

The Gibson Assembly Kit (New England Biolabs, Ipswich, MA) was used to replace the CBA promoter in the pAAV-CBA-W backbone with the human Adiponectin enhancer and promoter sequences (pAAV-hAdiponectin-W). Mouse *Fgf6* and *Fgf9* complementary DNAs (cDNAs) were cloned from cDNA clones (Origene). *Gfp* cDNA was derived from pLenti-GFP-Blast (Addgene, plasmid #17445). The pAAV-hAdiponectin-W backbone was linearized using *Spe*I/*Not*I (for *Fgf6*) or *Xho*I/*Not*I (for *Fgf9* and *Gfp*) and ligated with each insert.

### AAV administration to ingWAT

Ten-week-old C57BL6 mice were anesthetized using avertin injection. Two small bilateral incisions were made slightly above the hind limb. AAV2/8 serotype particles were directly injected into ingWAT at a dose of 10^11^ genome copy (GC) to each side.

For in vivo luciferase reporter experiments, Ucp1-cre mice (B6.FVB-Tg(Ucp1-cre)1Evdr/J, stock no. 024670) were bred with Rosa26-Luciferase (FVB.129S6(B6)-Gt(ROSA)26Sortm1(Luc)Kael/J, stock no. 005125), both obtained from The Jackson Laboratory. Offspring carrying the Ucp1-cre allele were injected with AAV2/8 serotype particles encoding FGF9 or GFP as described above. Mice were anesthetized with isoflurane and imaged using the IVIS Spectrum CT In Vivo Imaging System (PerkinElmer). Data were analyzed using the Living Image Software.

### Immunohistochemistry

IngWATs were fixed in 10% formalin and paraffin embedded. Multiple 5 μm sections were prepared and stained for UCP1. Briefly, sections were deparaffinized and rehydrated, followed by an antigen retrieval step in a modified citrate buffer (Dako Target Retrieval Solution, pH 6.1, Agilent). To reduce autofluorescence signal in adipose tissue, sections were then incubated in Sudan Black (0.3% in 70% ethanol). This was followed by blocking in Millipore blocking reagent (EMD Millipore) and then incubating with anti-UCP1 antibody (ab23841, Abcam, 1:250) overnight at 4 °C. The next day, slides were washed in PBS and were incubated with goat anti-rabbit immunoglobulin G (IgG) (H + L) secondary antibody conjugated with Alexa Fluor 594 (Invitrogen, 1:200). Nuclei were stained using DAPI (4′,6-diamidino-2-phenylindole).

### Generation of stable knockdown cell lines

Lentiviral vectors with FGFR1–4, PTGES, and LacZ shRNAs were purchased from GE Dharmacon (Lafayette, CO). Lentiviral particles were generated in HEK 293T cells and used for transduction of immortalized brown preadipocytes. Infected cells were selected with puromycin (1 μg/ml) for 7 days and expanded.

### Transient transfection of ERRA, FLII, and LRRFIP1 siRNAs

Immortalized brown preadipocytes were nucleofected with either ERRA small interfering RNA (siRNA), FLII siRNA, LRRFIP1 siRNA, or scrambled siRNA (Dharmacon GE) using the Lonza 4D-Nucleofector^TM^ X Kit (Basel, Switzerland), according to the manufacturer’s instructions (undifferentiated 3T3-L1 cell program). Cells were allowed to adhere and grow for 24 h before treatment with FGF6/9.

### PGE2 measurement

PGE2 concentrations were measured using the Prostaglandin E_2_ Express EIA Kit (Cayman Chemical, Ann Arbor, MI) according to the manufacturer’s instructions.

### RNA-seq and bioinformatics

Immortalized murine brown preadipocytes were treated with vehicle or FGF6 for 4, 8, and 24 h. Total RNA was isolated using the Direct-zol™ RNA Miniprep Kits (Zymo Research, Irvine, CA). High-throughput sequencing was performed using a HiSeq 4000 Instrument (Illumina) at BGI Americas. Mouse genome (GRCm38.p4) and gene annotation files were downloaded from Genecode. RNA-seq reads were aligned to the reference genome by using STAR and counted with Subread featureCounts. Normalization factors were obtained by using the weighted trimmed mean of *M* values method. Read counts were transformed to log 2 counts per million, their mean-variance relationship was estimated, and their observational-level weights were computed with voom. Differential gene expression was assessed by using linear modeling with limma. *P* values were corrected by using the Benjamini–Hochberg false discovery rate (FDR), and FDR <0.25 was considered statistically significant. Gene sets based on Kyoto Encyclopedia of Genes and Genomes (KEGG) were downloaded from the Molecular Signatures Database and the gene set enrichment was tested by using the limma Roast method.

### Engineered DNA-binding molecule-mediated ChIP

enChIP was performed according to the published protocol^60^ with some modifications. Stable murine brown preadipocytes overexpressing 3×FLAG-dCas9 were generated using the 3×FLAG-dCas9/pMXs-puro construct (Addgene, plasmid #51240, a gift from Dr. Hodaka Fujii’s laboratory). An empty gRNA expression vector (Addgene, plasmid #41824, a gift from Dr. George Church’s laboratory) was used to deliver five gRNAs spaced over the putative *Ucp1* regulatory region. gRNA sequences are as follows: TTCATCTTATATGTTGTGC, CACCCGCGTCCCCGCAGCG, GATAAGAAGTTACGACGGG, ATTCTAGCCTCGGCAGCCC, and TTTGGGAGTGACGCGCGGC.

3xFLAG-dCas9-overexpressing cells were nucleofected with either a mix of all five gRNA expression vectors or pMax-GFP control. A total of eight million cells were used for each condition. Cells were allowed to express gRNAs for 24 h, and then treated with vehicle or FGF6 for 48 h before enChIP experiment.

Cells were treated with 1% formaldehyde (Thermo Fisher Scientific, Waltham, MA) for 10 min to crosslink DNA–protein complexes. Cells were lysed in cell lysis buffer (10 mM Tris-HCl, pH 8.0, 1 mM EDTA, 0.5% IGEPAL CA-630, 1× protease inhibitors) and centrifuged to remove the cytoplasmic fraction in the supernatant. Nuclei were further lysed in nuclear lysis buffer (10 mM Tris-HCl, pH 8.0, 1 mM EDTA, 0.5 M NaCl, 1% Triton X-100, 0.5% sodium deoxycholate, 0.5% lauroylsarcosine sodium salt, 1× protease inhibitors), centrifuged, and resuspended in modified lysis buffer 3 (10 mM Tris-HCl, pH 8.0, 1 mM EDTA, 0.5 mM EGTA, 150 mM NaCl, 0.1% sodium deoxycholate, 0.1% SDS, 1× protease inhibitors), and chromatin was sonicated to 100–500 bp fragments. Sonicated chromatin was then incubated with FLAG-M2-magnetic beads (Sigma-Aldrich, Dallas, TX) overnight at 4 °C. The next day, beads were washed twice with low salt buffer (20 mM Tris-HCl, pH 8.0, 2 mM EDTA, 150 mM NaCl, 1% Triton X-100, 0.1% SDS), twice with high salt buffer (20 mM Tris-HCl, pH 8.0, 2 mM EDTA, 500 mM NaCl, 1% Triton X-100, 0.1% SDS), twice with LiCl buffer (10 mM Tris-HCl, pH 8.0, 1 mM EDTA, 0.25 M LiCl, 0.5% IGEPAL CA-630, 0.5% sodium deoxycholate), and twice with TBS (50 mM Tris-HCl, pH 7.5, 150 mM NaCl) containing 0.1% IGEPAL CA-630. Bound DNA–protein complexes were eluted with 3×FLAG peptide (F4799, Sigma-Aldrich, Dallas, TX) in TBS containing 0.1% IGEPAL CA-630. Eluted proteins were later TCA precipitated, were resuspended in 6 M urea, 50 mM Tris, pH 8.5, reduced with dithiothreitol, and alkylated. Proteins were digested overnight with trypsin, cleaned, and subjected to TMT labeling, followed by liquid chromatography-mass spectrometry (LC-MS) at the Thermo Fisher Scientific Center for Multiplexed Proteomics at Harvard Medical School (Boston, MA).

### Chromatin immunoprecipitation

Cells were treated with 1% formaldehyde (Thermo Fisher Scientific, Waltham, MA) for 10 min to crosslink DNA–protein complexes. Glycine was added to a final concentration of 125 mM for 5 min to quench crosslinking. Cells were washed with PBS, harvested, and centrifuged at 900 × *g* for 3 min. The pellet was resuspended in ChIP lysis buffer (20 mM Tris-HCl, pH = 8, 0.6% SDS, 1% Triton X-100, 150 mM NaCl, 1 mM EDTA), and chromatin was sonicated to 200–1000 bp fragments. Sonicated chromatin was further diluted with ChIP dilution buffer (20 mM Tris-HCl, 1% Triton X-100, 150 mM NaCl, 1 mM EDTA), and incubated with antibodies or IgG control at 4 °C overnight. The following day, Dynabeads Protein G (10003D, Thermo Fisher Scientific, Waltham, MA) were added and incubated at 4 °C for 2–4 h. Beads were washed twice with low salt buffer (20 mM Tris-HCl, pH 8.0, 1 mM EDTA, 150 mM NaCl, 1% Triton X-100, 0.1% SDS, 0.1% sodium deoxycholate), once with high salt buffer (20 mM Tris-HCl, pH 8.0, 1 mM EDTA, 500 mM NaCl, 1% Triton X-100, 0.1% SDS, 0.1% sodium deoxycholate), once with LiCl buffer (20 mM Tris-HCl, pH 8.0, 1 mM EDTA, 0.25 M LiCl, 0.5% NP-40, 0.5% sodium deoxycholate), and finally washed twice in 20 mM Tris pH 8.0, 1 mM EDTA. Samples were then eluted in 200 μL of elution buffer (1 mM EDTA, 1% SDS). Reverse crosslinking was performed with the addition of RNAse A at 65 °C overnight. Samples were then incubated with proteinase K for 2 h at 55 °C, and purified using QIAquick PCR Purification Kit (Qiagen).The following antibodies were used for ChIP experiments (1 μg/ChIP): anti-ERRA (ab16363, Abcam), anti-H3K27ac (ab4729, Abcam), anti-H3K4me3 (07-473, Millipore), and H3K9me (ab1220, Abcam).

Primers used for qPCR of mouse UCP1 regulatory region are as listed:

*Ucp1* TSS: Forward: TTTTGTTCTTGCACTCACGCC,

Reverse: CCCATGGTGGGTTGCACTTC.

*Ucp1* promoter: Forward: TGTGGCCAGGGCTTTGGGAGT,

Reverse: AGATTGCCC GGCACTTCTGCG.

*Ucp1* distal enhancer: Forward: AGCTTGCTGTCACTCCTCTACA,

Reverse: TGAGGAAAGGGTTGACCTTG.

Upstream control region: Forward: GCTTGGGTCCACCTAGAATCAC,

Reverse: CCTCCAGGTCAAACTGATCTAGACA.

### Immunoprecipitation

For ERRA IP-MS experiment, murine brown preadipocytes were nucleofected with a plasmid overexpressing DDK-tagged ERRA (MR225753, Origene). Cells were lysed in Pierce IP Lysis Buffer (Thermo Fisher Scientific, Waltham, MA). ERRA was pulled down using FLAG-M2 magnetic beads (Sigma-Aldrich, Dallas, TX). Immunoprecipitated proteins were subjected to TMT labeling, followed by LC-MS at the Thermo Fisher Scientific Center for Multiplexed Proteomics at Harvard Medical School (Boston, MA).

A fraction of samples were blotted with ERRA and FLII antibodies for detection of interaction. Rabbit IgG was used as a control (2729, Cell Signaling Technologies).

### Overexpression of ERRA, FLII, and LRRFIP1

Immortalized brown preadipocytes were nucleofected with cDNA plasmids encoding Erra, Flii, Lrrfip1, or a combination of them using the Lonza 4D-Nucleofector^TM^ X Kit (Basel, Switzerland), according to the manufacturer’s instructions (undifferentiated 3T3-L1 cell program).

### FGF9 protein expression and purification

An expression construct for human FGF9 protein encompassing residues 35–208 and carrying an monomerizing triple alanine substitution (i.e., D195A/I204A/L205A) has previously been described^61^. An endocrinized version of this construct (termed FGF9^ΔHBS^) was engineered by introducing a triple mutation (i.e., K168Q/R173V/R177Q) into the HS-binding site of FGF9 using a NEB Q5^®^ Site-Directed Mutagenesis Kit. This triple mutation severely diminishes the HS-binding affinity of FGF9, thereby imparting a major reduction in the mutant ligand’s FGFR dimerization ability and mitogenic activity. Competent *Escherichia coli* BL-21 (DE3) cells transformed with FGF9 constructs were cultured in 1 L LB medium containing 30 μg/mL kanamycin in an incubator shaker at 37 °C and 200 r.p.m. At an optical density of 0.8–1.0 at *λ*_600_, recombinant protein expression was induced by the addition of isopropyl-l-thio-β-d-galactopyranoside to 1 mM and further growth at 20 °C overnight. Cells were harvested by centrifugation at 5020 × *g* (Beckman Coulter J6-M1, ROTOR JS-4.2) for 30 min at 4 °C and lysed in 64 ml of 25 mM HEPES, pH 7.5 buffer containing 150 mM NaCl, 5 mM EDTA, and 10% glycerol using an Emulsiflex-C3 (Avestin Inc., Ottawa, ON, Canada) high-volume homogenizer. Soluble lysates containing recombinant FGF9 proteins were clarified by centrifugation at 39,200 × *g* (Beckman Coulter Avanti J-25, ROTOR JA-25.50) for 60 min at 4 °C, filtered using a 0.45 μm membrane (Nalgene, catalog# 295-3345) and loaded onto a 5 ml heparin affinity (GE Healthcare, Heparin Sepharose CL-6B, catalog #17-0467-01) gravity column. Following 5 column volume (CV) wash with 25 mM HEPES, pH 7.5 buffer bound FGF9 proteins were eluted stepwise with 5 mL column × 5 volume of HEPES 25 mM, 1 M NaCl, followed by 5 mL × 5 volume of HEPES 25 mM, 2 M NaCl buffer. Fractions containing FGF9 proteins as determined by analysis via 12% SDS–polyacrylamide gel electrophoresis (SDS-PAGE) were pooled, diluted with 25 mM HEPES, pH 7.5 buffer, to 25 mM NaCl, and loaded onto 5 mL heparin affinity HiTrap column (GE Healthcare, catalog #17-0407-03) equilibrated in 25 mM HEPES, pH 7.5 buffer. FGF9 proteins were eluted with 16 CV linear NaCl gradient (0–2.0 M). Fractions containing FGF9 proteins were pooled, loaded to 20 mL Source 15S ion exchange column (GE Healthcare Life Sciences, Piscataway, NJ, product #17094405) equilibrated in 25 mM HEPES, pH 7.5 buffer. Bound FGF9 proteins were eluted using 13 CV linear NaCl gradient (0–0.5 M). Fractions containing FGF9 proteins were pooled, concentrated to <5 mL and applied to a gel filtration column (Superdex^TM^-75 GE Healthcare, Piscataway, NJ). Proteins were eluted isocratically in 25 mM HEPES, pH 7.5 buffer containing 500 mM NaCl. The purity of the recombinant proteins as judged by SDS-PAGE was estimated to be at least 98%.

### Administration of recombinant FGF9 proteins

Ten-week-old C57BL6 mice received daily subcutaneous injection of FGF9 proteins or vehicle (PBS).

### Cold tolerance test

Mice were exposed to 5 °C in a controlled environmental diurnal chamber (Caron Products & Services Inc., Marietta, OH) with free access to food and water. Core body temperature was determined by rectal probe measurements.

### Indirect calorimetry

Mice were injected intraperitoneally with pentobarbital (65 mg/kg). Basal energy expenditure was measured for 30 min before stimulation with NE (Sigma, 1 mg/kg in 0.9% (w/v) NaCl). Energy expenditure was measured using a Columbus Instruments’ Oxymax-Comprehensive Lab Animal Monitoring System system according to guidelines for measuring energy metabolism in mice^62^.

### Denervation of BAT

Denervation of BAT was performed as described before^63^. Briefly, five branches of the intercostal nerve bundles were isolated and cut. The procedure was performed on the left and right lobes of the interscapular brown fat. The same procedure was performed for sham surgeries, except nerve bundles were not cut. Animals were dissected 11 weeks after the denervation.

### Generation of FGF9-KO mice

Homozygous Rosa26-floxed STOP-Cas9 knock-in mice^64^ (B6;129-Gt(ROSA)^26Sortm1(CAG-cas9*,-EGFP)Fezh/J^, stock no. 024857, The Jackson Laboratory) were crossed with adiponectin cre mice^65^ (B6;FVB-Tg(Adipoq-cre)1Evdr/J, stock no. 010803, The Jackson Laboratory) or Ucp1-cre mice (B6.FVB-Tg(Ucp1-cre)1Evdr/J, stock no. 024670). Two gRNAs targeting the first exon of mouse *Fgf9* gene (*Fgf9* gRNA-1: GGGCCCCGCAGTCACGGACT and *Fgf9* gRNA-2: CTTCCCCAACGGTACTATCC) were cloned into pAAV-U6-BbsI-gRNA-CB-EmGFP plasmid (Addgene, plasmid #89060). The Fgf9 gRNAs or empty vector (GFP) were packaged in AAV2/8 particles and then bilaterally injected into both BAT lobes of 10-week-old Rosa26-Cas9-adiponectin cre or Rosa26-Cas9-ucp1 cre.

### Isolation of adipocytes and SVF from BAT

Interscapular BAT was dissected, minced, and digested with type 1 collagenase 1.5 mg/mL; Worthington Biochemical) in Hanks’ balanced salt’s solution (Lonza) containing 2% fatty acid-free BSA (Gemini Bio-products, West Sacramento, CA) for 45 min at 37 °C with gentle shaking. Dissociated tissue was centrifuged at 300 × *g* for 10 min. Adipocytes were collected from the top layer, filtered through a 200 μm cell strainer, washed with 3% BSA in PBS, and centrifuged at 300 × g for 5 min. The washing step was repeated three times and adipocytes were lysed TriPure RNA Isolation Reagent (Sigma-Aldrich).

After removing the adipocyte layer and supernatant, SVF pellet was washed with 10% FBS in DMEM, filtered through a 100 μm cell strainer, and centrifuged at 300 × *g* for 7 min. Cells were incubated in red blood cell lysis buffer (ACK Lysing Buffer, Lonza) for 5 min at 4 °C. ACK was then diluted by adding 10% FBS in DMEM. The cells were then filtered through a 40 μm cell strainer, centrifuged at 300 × *g* for 5 min, and lysed in TriPure RNA Isolation Reagent (Sigma-Aldrich).

### Flow cytometry

Homozygous Rosa26-mTmG mice (B6.129(Cg)-Gt(ROSA)^26Sortm4(ACTB-tdTomato,-EGFP)Luo^/J, stock no. 007676, The Jackson Laboratory) were crossed with Ucp1-cre mice (B6.FVB-Tg(Ucp1-cre)1Evdr/J, stock no. 024670, The Jackson Laboratory) or Ucp1-creERT2 mice (kindly provided by Dr. Christian Wolfrum). SVF was isolated form BAT as described above. In experiment involving Sca-1 staining, anti-mouse Sca-1 (Ly-6A/E, PerCP-Cy5.5 conjugate, clone E13-161.7, BioLegend) was used at 1:200 dilution. BD FACSAria (Becton Dickinson) was used and data were collected using DIVA (Becton Dickinson) software and analyzed using the FlowJo software (Tree Star Inc.). Debris and dead cells were excluded by forward and side scatter gating.

### Human studies

In this report, we utilize human adipose tissue biopsies collected from three independent cohorts. First cohort—Danish adult neck adipose tissue cohort: adipose tissue biopsies from the superficial (subcutaneous and subplatysmal) neck fat and deep (carotid sheath, longus colli, and prevertebral) neck fat were collected during surgery^66^ (*n* = 75). None of the subjects had diabetes nor were they administered β-adrenergic antagonists. All biopsies were collected during winter and early spring. Each participant had one biopsy taken from subcutaneous adipose tissue and up to three biopsies from deep-neck adipose tissue. This resulted in a total of 75 subcutaneous fat biopsies and 156 deep-neck fat biopsies that were instantly frozen in liquid nitrogen. All study participants gave informed written consent. The study was approved by the Central Denmark Region ethics committee and was performed in accordance with the Declaration of Helsinki. *UCP1*, *FGF6*, *FGF9*, and *FGFR3* mRNA expression was analyzed using qRT-PCR as described above.

Second cohort—Leipzig adult adipose tissue cohort: Abdominal subcutaneous adipose tissue biopsies were collected from 55 individuals with a wide range of BMI (16–75 kg/m^2^), type 2 diabetes (*n* = 18) and normal glucose metabolism (*n* = 37). Adipose tissue biopsies were taken during elective sleeve gastrectomy, Roux-en-Y gastric bypass, hernia, or cholecystectomy surgeries and processed^67^. Written informed consent was obtained from all parents. The study was approved by the local ethics committee (University of Leipzig). *FGF6*, *FGF9*, and *FGFR3* mRNA expression was analyzed using Illumina human HT-12 expression chips. RNA integrity and concentration were examined using Agilent 2100 Bioanalyzer (Agilent Technologies, Palo Alto, CA, USA).

Third cohort—Leipzig childhood adipose tissue cohort: Subcutaneous adipose tissue samples were obtained from 301 Caucasian children (aged 0–18 years) undergoing elective surgery (e.g., orthopedic surgery, herniotomy/orchidopexy). Children were free of severe disease and medication potentially influencing adipose tissue biology. Exclusion criteria included diabetes, generalized inflammation, cardiovascular or peripheral artery disease, malignant disease, genetic syndromesr, and permanent immobility. Written informed consent was obtained from all parents. The study was approved by the local ethics committee (Reg. No.: 265-08, 265-08-ff, University of Leipzig) and is registered in the National Clinical Trials database (NCT02208141 [https://clinicaltrials.gov/ct2/show/NCT03690193?term=NCT02208141]). The Leipzig Childhood Obesity study is a completely observational study without any interventions or treatments. BMI data were standardized to age- and sex-specific German reference data and are given as BMI standard deviation score^68^. Insulin and glucose levels were measured in the fasted state by a certified laboratory. HOMA-IR index was calculated as a parameter to evaluate insulin resistance^69^. Gene expression was quantified by ILLUMINA HT12v4 arrays and data were background corrected and quantile normalized.

### Statistical analysis

All statistics were calculated using Microsoft Excel, Graphpad Prism, and RStudio using the LIMMA package. The data are presented as means ± SEM. Statistical significance was determined by Student’s *t* test, one- or two-way ANOVA (analysis of variance). Multiple comparisons were corrected for by controlling the FDR using two-stage step-up method of Benjamini, Krieger, and Yekutueli in PRISM. In qPCR experiments, normalized Ct (ΔCt) values were used for statistical analysis^70^. No statistical method was used to determine sample size. All experiments were not blinded.

### Reporting summary

Further information on research design is available in the Nature Research Reporting Summary linked to this article.

## Supplementary information

Supplementary Information

Description of Additional Supplementary Information

Supplementary Dataset 1

Supplementary Dataset 2

Supplementary Dataset 3

Supplementary Dataset 4

Reporting Summary

## Data Availability

The authors declare that the data supporting the findings of this study are available within the paper and its supplementary information files. The source data underlying the figures are provided as a Source Data file. RNA-sequencing data have been deposited in the Gene Expression Omnibus (GEO accession #GSE144061).
