# Supplementary material for: FGF6 and FGF9 regulate UCP1 expression independent of brown adipogenesis
Source: Nat Commun. 2020 Mar 17;11:1421. doi: 10.1038/s41467-020-15055-9 (PMC7078224; doi:10.1038/s41467-020-15055-9)
Supplement: Supplementary file 1 — Supplementary Information [file 41467_2020_15055_MOESM1_ESM.pdf]

Supplementary Information

FGF6 and FGF9 regulate UCP1 expression independent of brown adipogenesis

Shamsi et al.

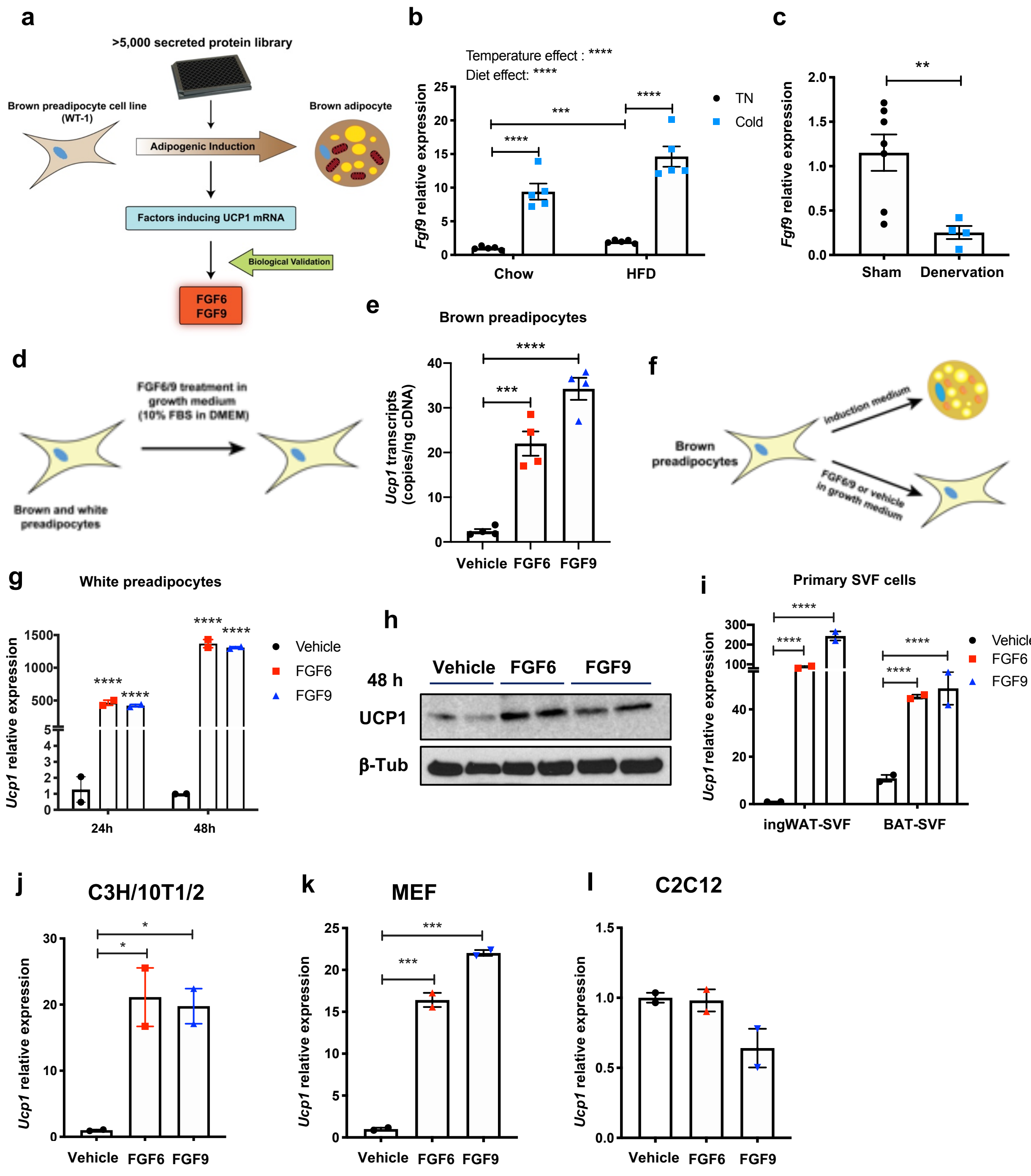

Supplementary Figure 1. FGF6 and FGF9 induce *Ucp1* expression in preadipocytes and uncommitted progenitor cells, related to Figure 1. (a) Scheme of the high throughput screen using secreted protein library. (b) *Fgf9* mRNA expression in mice fed with chow or HFD and housed at either 5°C (cold) or 30°C for 7 days (TN). (c) *Fgf9* mRNA expression in BAT from sham-operated or denervated BAT

upon 10 days injection with CL-316,243. (d) Scheme of FGF6/9 treatment in murine preadipocytes. (e) Absolute quantification of *Ucp1* transcripts in murine brown preadipocytes treated with vehicle, FGF6, or FGF9 for 24 hours using Droplet Digital™ PCR Technology. N=4 per group. (f) Scheme of treatment groups used in figure 1f-g. (g) *Ucp1* mRNA and (h) protein in murine white preadipocytes treated with vehicle, FGF6, or FGF9 for 24 and 48 hours, respectively. N=3 per group. 30 μg total protein was used for WB. (i) *Ucp1* gene expression in primary brown and white SVF cells treated with vehicle, FGF6, or FGF9 for 3 days. N=3 per group. (j) *Ucp1* gene expression in C3H/10T1/2 cells treated for 24 hours, (k) MEF, and (l) C2C12 treated for 3 days with vehicle, FGF6, or FGF9. N=3 per group. FGF6 and FGF9 were used at concentration of 200 ng/ml and 100 ng/ml, respectively. Data are presented as Means ± SEM. Two -way ANOVA in b, g, and i. Two-sample t-test in c. One -way ANOVA in e, j, and k. ****$p < 0.0001$, ***$p < 0.001$, **$p < 0.01$, *$p < 0.05$. A representative from a total of 2-3 independent experiments is shown. Source data are provided as a Source Data file.

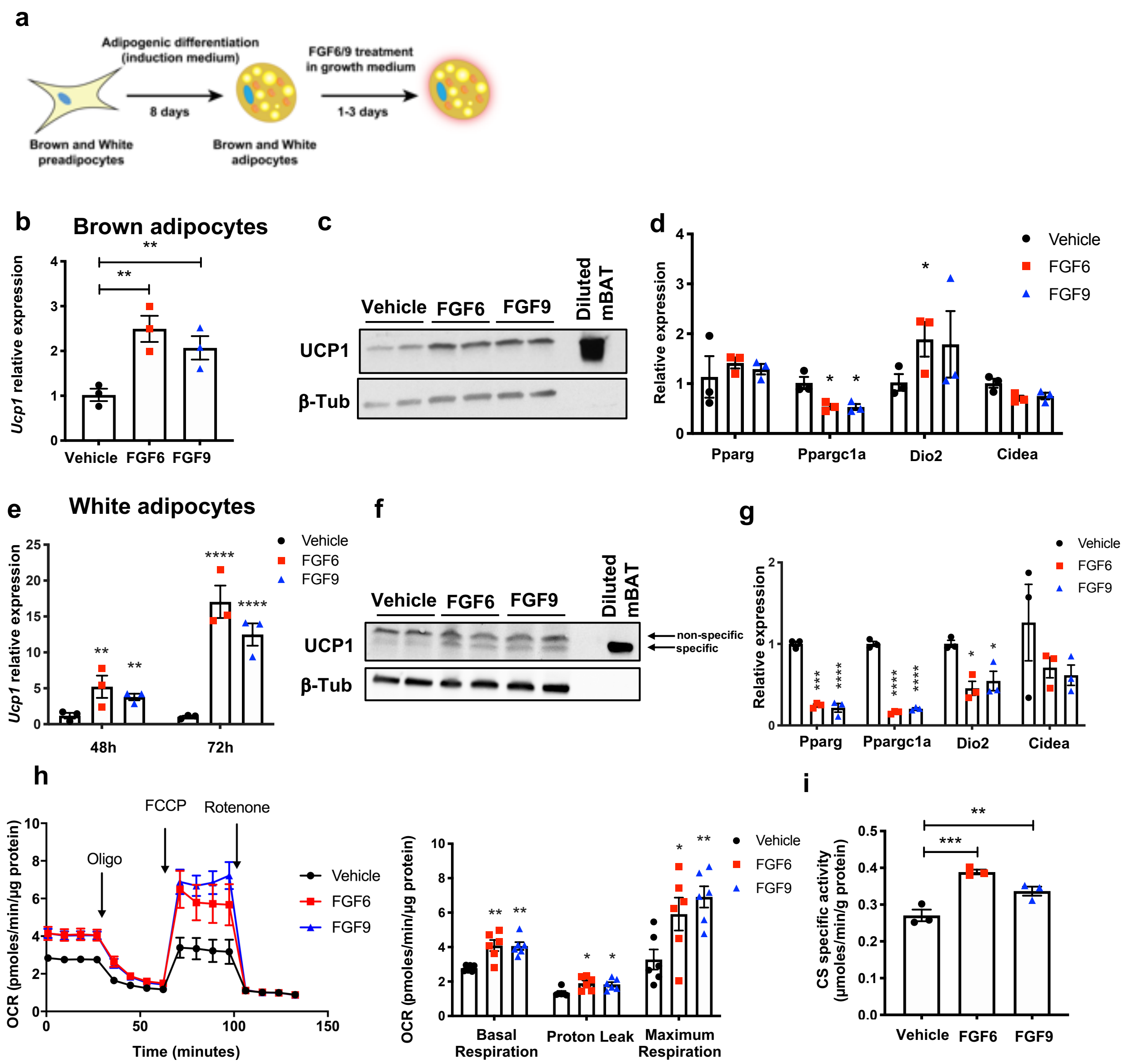

Supplementary Figure 2. FGF6 and FGF9 induce UCP1 expression in brown and white adipocytes, related to Figure 1. (a) Scheme of FGF6/9 treatment in differentiated murine adipocytes. Murine brown and white preadipocytes were differentiated using the conventional adipogenic induction media for 8 days, and then exposed to FGF6 or FGF9 in growth medium. Expression of (b) *Ucp1* mRNA, (c) protein (using 50 µg total protein from cells and 5 µg from mouse BAT), and (d) other brown adipocyte genes in differentiated brown adipocytes treated with vehicle, FGF6, or FGF9 for 24 hours. N=3 per group. Expression of (e) *Ucp1* mRNA, (f) protein (using 45 µg total protein from cells and 0.25 µg from mouse BAT), and (g) other brown adipocyte genes in differentiated white adipocytes treated with vehicle, FGF6,

or FGF9 for 48 hours. N=3 per group. (h) Left: Oxygen consumption in differentiated white adipocytes treated with vehicle, FGF6, or FGF9 for 48 hours. Right: quantifications of specific cellular respiration. N=6-7 per group. (i) CS specific activity in differentiated white adipocytes treated with vehicle, FGF6, or FGF9 for 48 hours. N=3 per group. FGF6 and FGF9 were used at concentration of 200 ng/ml and 100 ng/ml, respectively. Data are presented as Means ± SEM. One-way ANOVA in b and i. Two- way ANOVA in d-h. ****$p < 0.0001$, ***$p < 0.001$, **$p < 0.01$, *$p < 0.05$. A representative from a total of 2-3 independent experiments is shown. Source data are provided as a Source Data file.

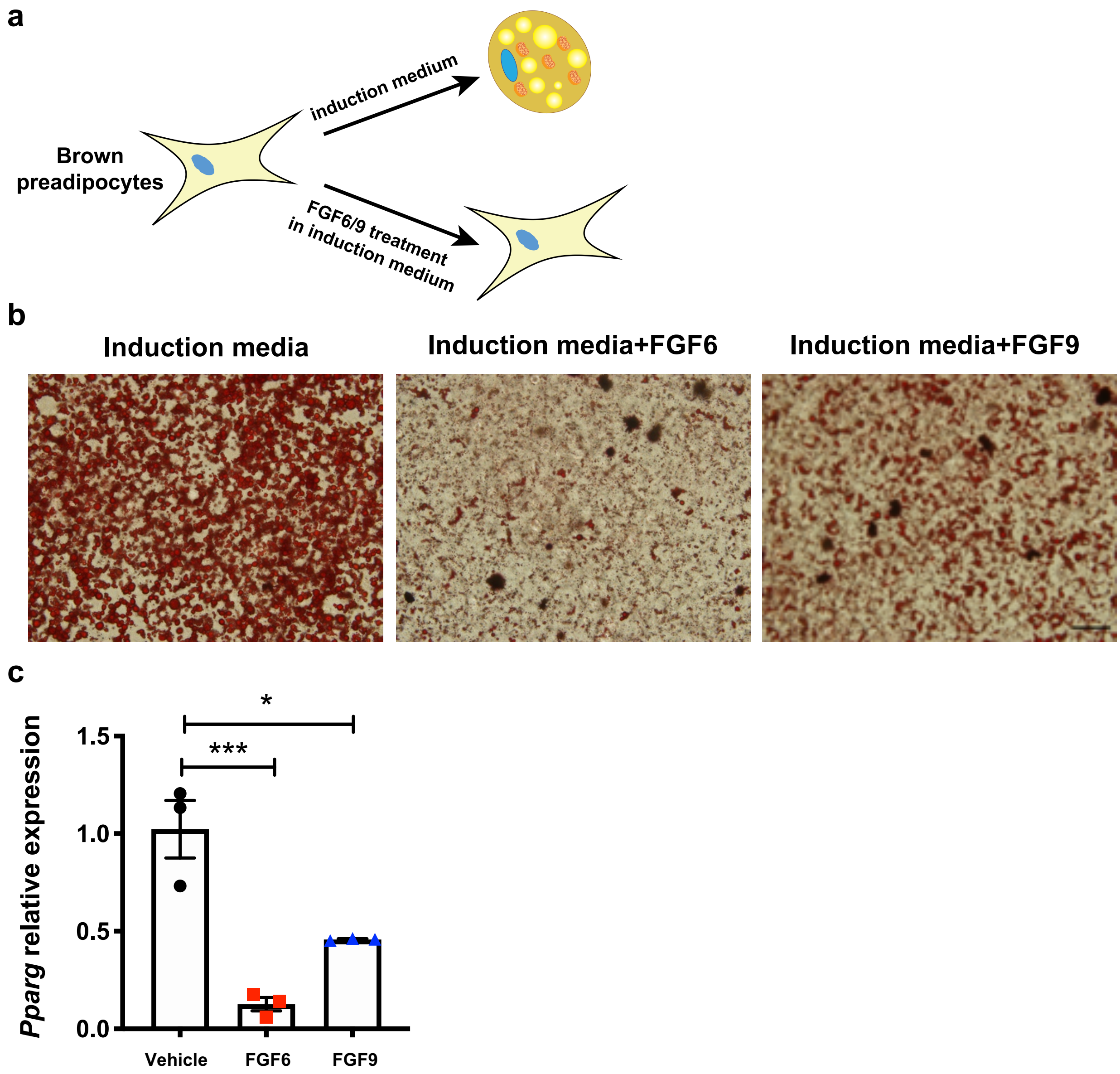

Supplementary Figure 3. FGF6 and FGF9 suppress adipocyte differentiation *in vitro*, related to Figure 1. (a) Scheme of FGF6/9 treatment during the adipogenic differentiation of murine brown preadipocytes. (b) Oil-red O staining and (c) *Pparg* expression in brown preadipocytes treated with adipogenic induction media supplemented with vehicle, FGF6, or FGF9 for 8 days. Scale bar = 200 μm. Data are presented as Means ± SEM. One-way ANOVA. ***p < 0.001, *p < 0.05. A representative from a total of 2 independent experiments is shown. Source data are provided as a Source Data file.

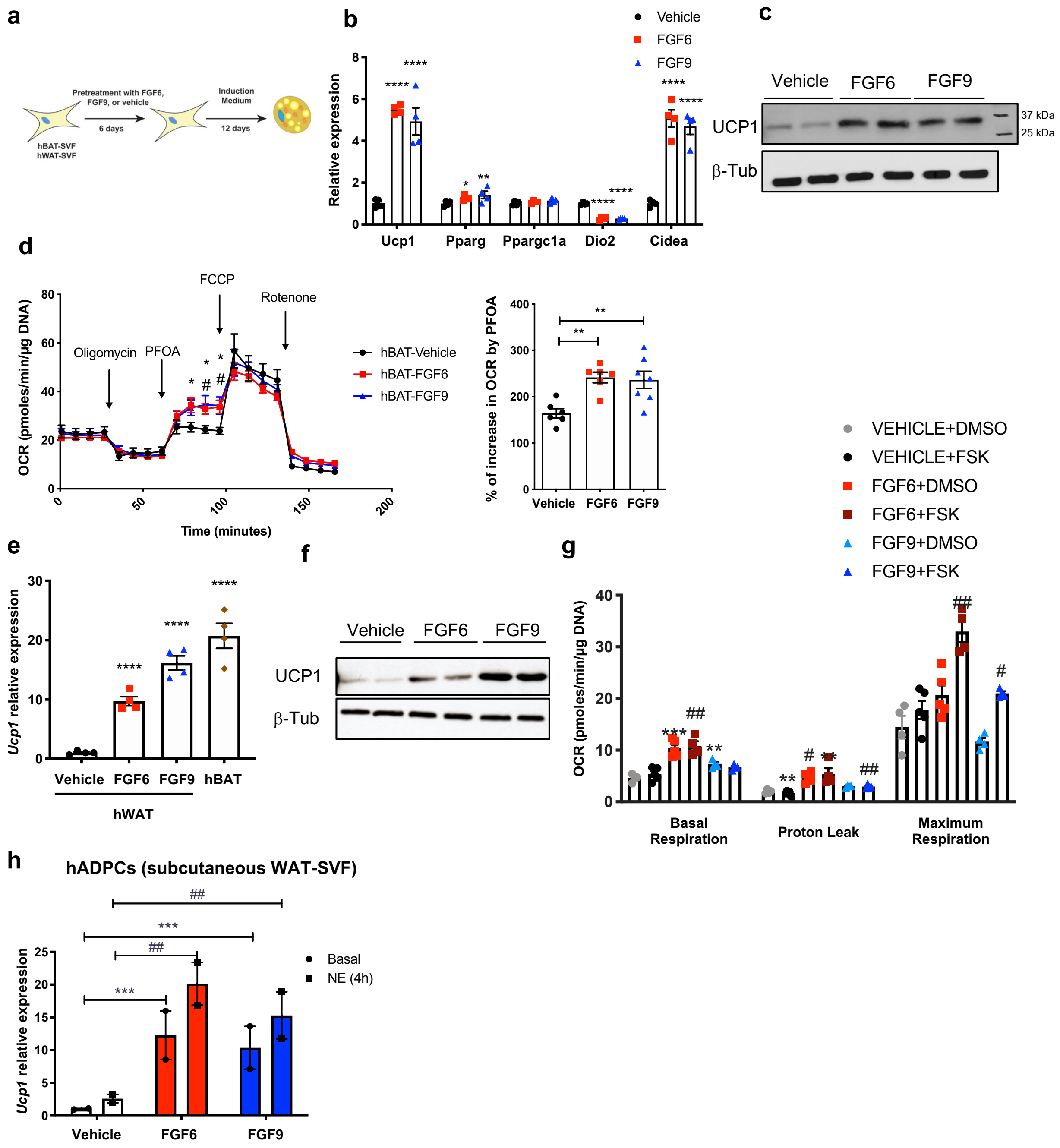

Supplementary Figure 4. FGF6 and FGF9 prime human preadipocytes to acquire higher thermogenic capacity, related to Figure 1. Immortalized human brown or white preadipocytes of neck origin were treated with FGF6 and FGF9, and then differentiated using an adipogenic differentiation cocktail. (a) Scheme of FGF pretreatment in human preadipocytes followed by adipogenic differentiation. (b) Gene expression (c) UCP1 protein (using 80 μg total protein), and (d) left: oxygen consumption measured by mitochondrial stress test (FGF6 vs Vehicle, #: FGF9 vs Vehicle). right: quantifications of

specific cellular respiration in human brown adipocytes pretreated with vehicle, FGF6, or FGF9. N=3-7 per group. (e) *UCP1* mRNA in human white adipocytes pretreated with vehicle, FGF6, or FGF9, compared to human brown adipocytes. N=3 per group (f) UCP1 protein (using 80 μg total protein) in human white adipocytes pretreated with vehicle, FGF6, or FGF9. N=2 per group. (g) Quantification of specific cellular respiration in human white adipocytes pretreated with vehicle, FGF6, or FGF9, followed by 4 hours Forskolin (FSK) stimulation. N=6-7 per group. (h) UCP1 expression in human primary white adipocytes pretreated with vehicle, FGF6, or FGF9, followed by 4 hours Norepinephrine (NE) stimulation. N=3 per group. FGF6 and FGF9 were used at concentration of 200 ng/ml and 100 ng/ml, respectively. Data are presented as Means ± SEM. Two-way ANOVA in b, d (left), g, and h. One-way ANOVA in d (right) and e. ****$p < 0.0001$, ***$p < 0.001$, **$p < 0.01$. *: relative to Veh-DMSO, #: relative to Veh-FSK. A representative from a total of 2-3 independent experiments is shown. Source data are provided as a Source Data file.

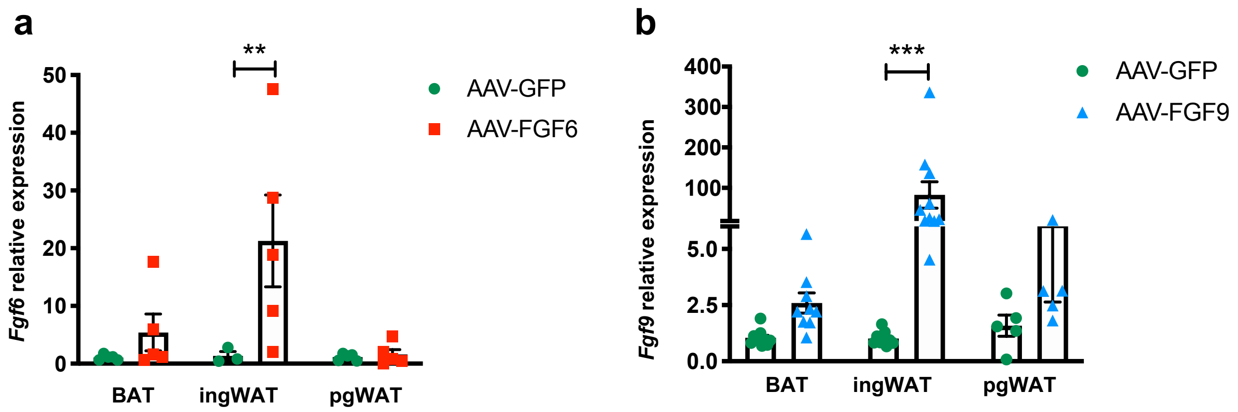

Supplementary Figure 5. FGF6 and FGF9 overexpression in adipose tissue, related to Figure 1. (a) *Fgf6* and (b) *Fgf9* expression in BAT, ingWAT, and pgWAT of mice injected with AAV-GFP, AAV-FGF6, or AAV-FGF9. N=5-6 per group. Two-way ANOVA. *$p < 0.05$. A representative from a total of 3 independent experiments is shown. Source data are provided as a Source Data file.

a

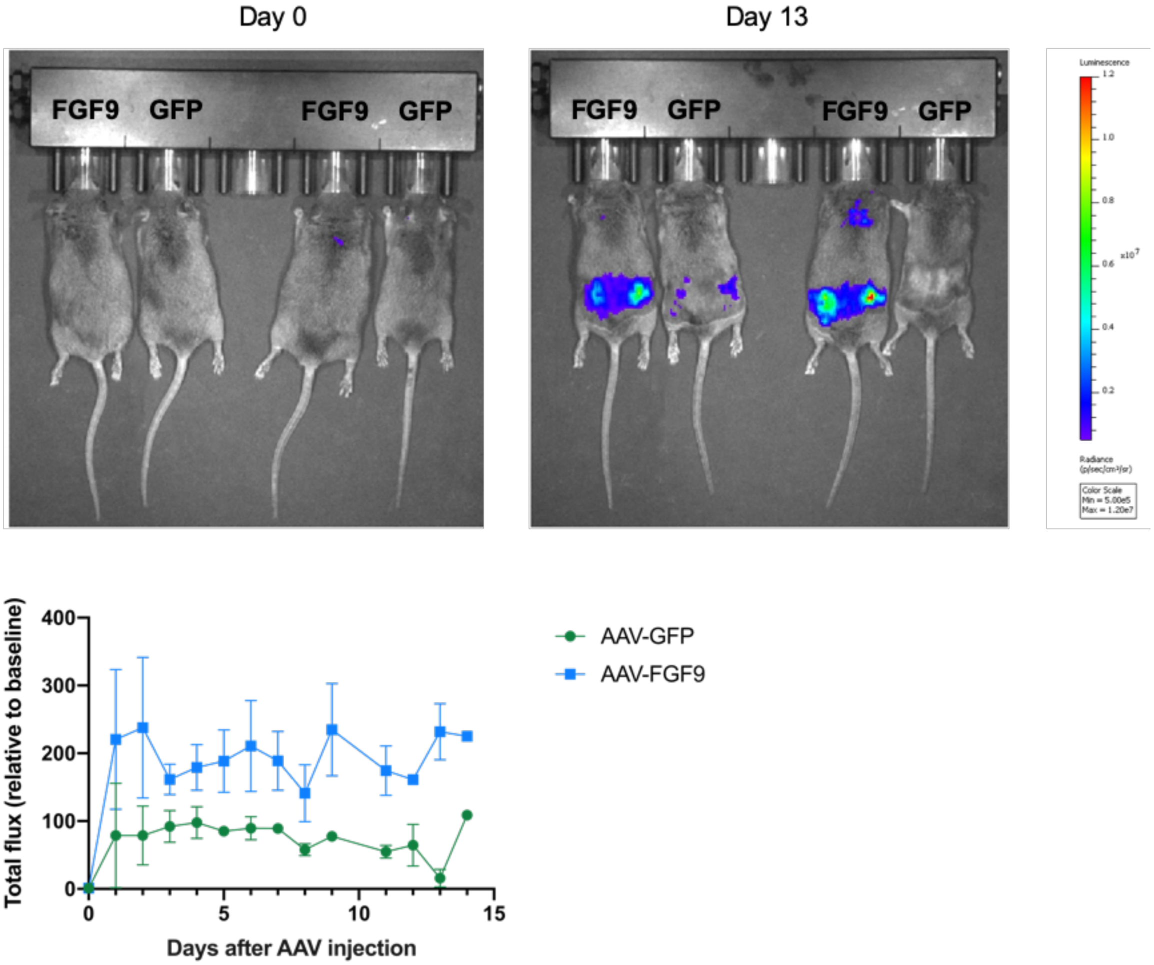
Day 0
Day 13
FGF9 GFP FGF9 GFP
FGF9 GFP FGF9 GFP
Total flux (relative to baseline)
Days after AAV injection
AAV-GFP
AAV-FGF9

b

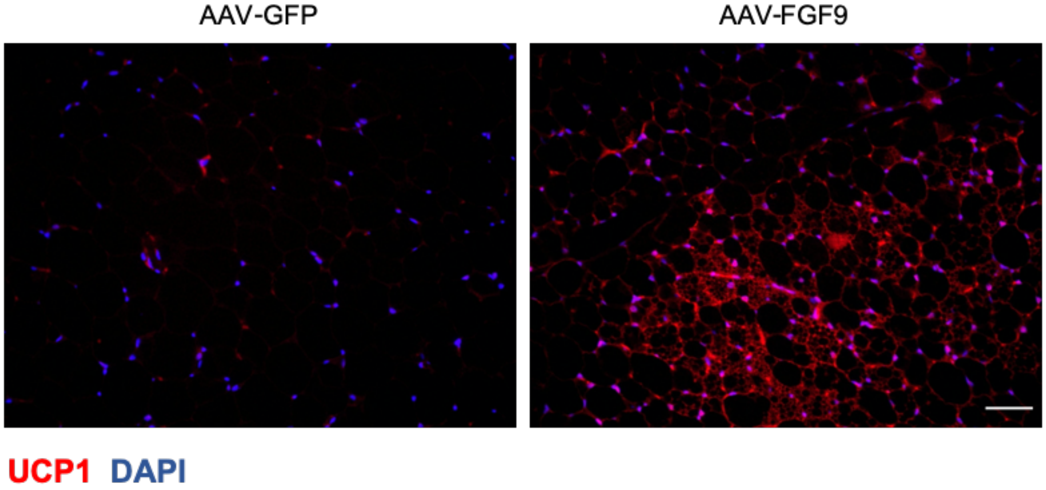
AAV-GFP
AAV-FGF9
UCP1 DAPI

Supplementary Figure 6. Overexpression of FGF9 induces *Ucp1* expression in ingWAT, related to Figure 1. (a) Representative images (top) and quantification (bottom) of Luciferase signal by IVIS in Ucp1-cre Rosa26-Luciferase reporter mice injected with either AAV-FGF9 or AAV-GFP. Quantification presents the changes in total flux [photon/s] in the same selected region of interest (ROI) relative to the baseline (b) UCP1 immunohistochemistry on ingWAT of Ucp1-cre Rosa26-Luciferase reporter mice injected with either AAV-FGF9 or AAV-GFP. Scale bar = 200 μm.

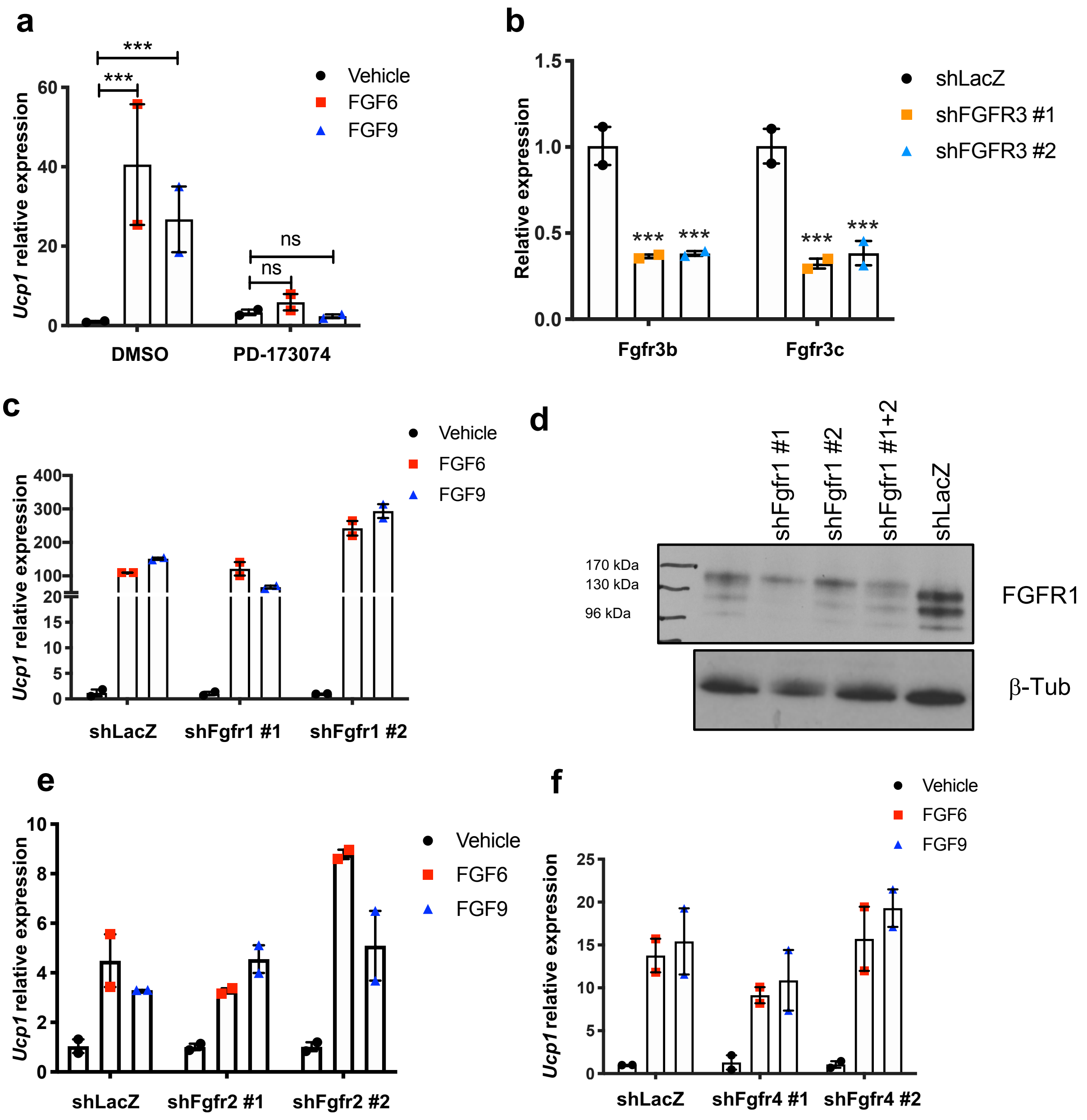

Supplementary Figure 7. FGF6 and FGF9 induce UCP1 expression through binding to FGFR3, related to Figure 2. (a) *Ucp1* expression in white preadipocytes treated with vehicle, FGF6, or FGF9 in the presence of pan-FGFR antagonist, PD-173074, or equimolar concentration of DMSO. N=3 per group. (b) *Fgfr3b* and *Fgfr3c* expression in control and *Fgfr3* knockdown cells. N=3 per group**.** (c) *Ucp1* expression in control (shLacZ) and *Fgfr1* knockdown cells treated with vehicle, FGF6, or FGF9 for 24 hours. N=3 per group. (d) FGFR1 protein level in control (shLacZ) and *Fgfr1* knockdown cells. 50 μg total protein was used. (e) *Ucp1* expression in control (shLacZ) and *Fgfr2* knockdown cells treated with vehicle, FGF6, or FGF9 for

24 hours. N=3 per group. (f) *Ucp1* expression in control (shLacZ) and *Fgfr4* knockdown cells treated with vehicle, FGF6, or FGF9 for 24 hours. N=3 per group. FGF6 and FGF9 were used at concentration of 200 ng/ml and 100 ng/ml, respectively. Data are presented as Means ± SEM. Two-way ANOVA. ***p < 0.001. A representative from a total of 2-3 independent experiments is shown. Source data are provided as a Source Data file.

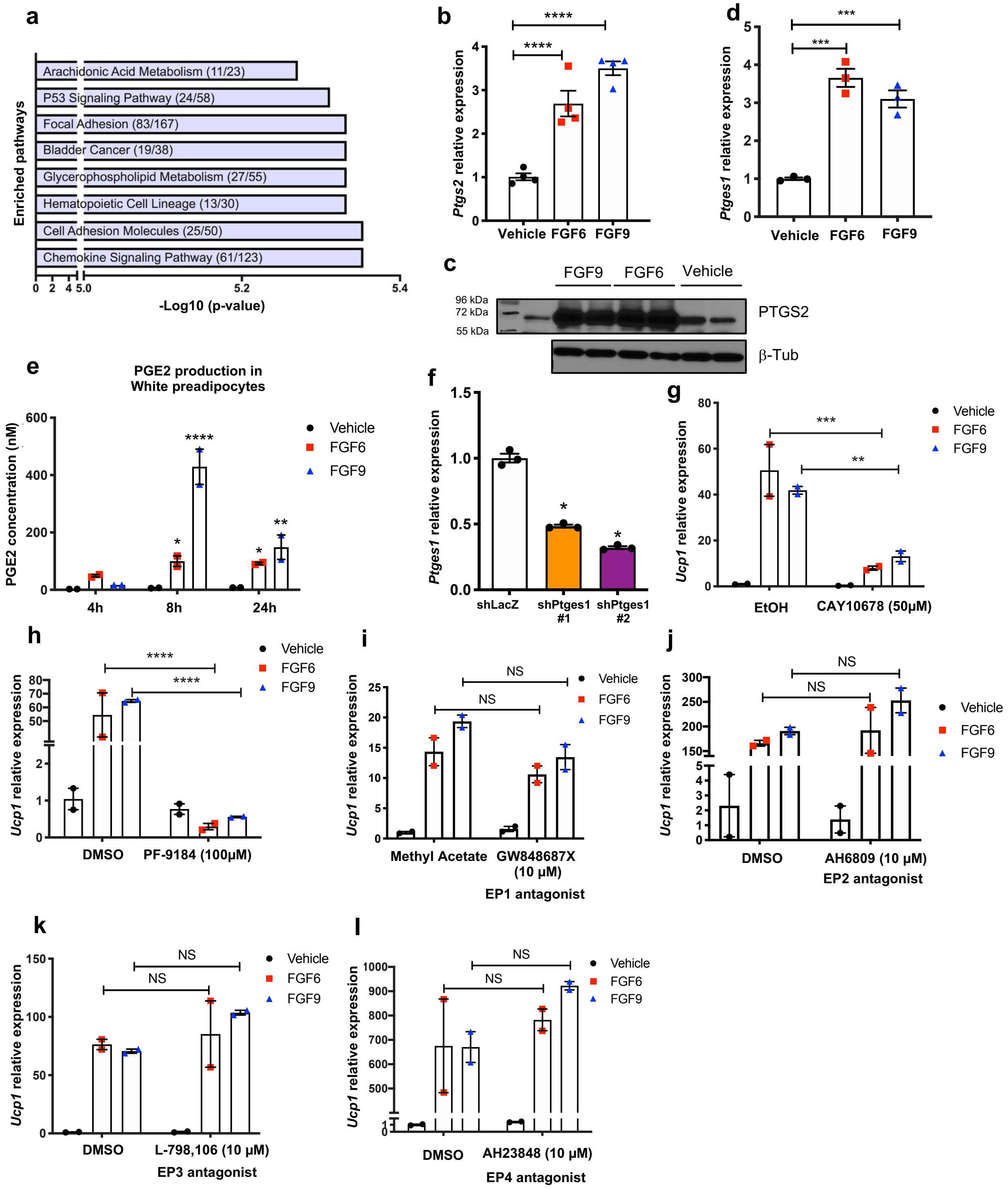

Supplementary Figure 8. FGF6- and FGF9-mediated UCP1 expression requires activation of PGE2 biosynthesis, related to Figure 2. (a) Selected top biological pathways enriched in brown preadipocytes after 24 hours treatment with FGF6. (b) *Ptgs2* mRNA and (c) protein in brown preadipocytes treated with

vehicle, FGF6, or FGF9 for 48h. N=3 per group. 50 μg total protein was used for WB. (d) *Ptges* expression in brown preadipocytes treated with vehicle, FGF6, or FGF9 for 24 hours. N=3 per group. (e) PGE2 concentration in the culture media of white preadipocytes upon treatment with vehicle, FGF6, or FGF9. N=3 per group. (f) *Ptges* expression in control (shLacZ) and *Ptges* knockdown cells. N=3 per group. (g) *Ucp1* expression in white preadipocytes treated with vehicle, FGF6, or FGF9 in the presence of PTGES selective antagonist, CAY10678 (50 μM), (h) PF-9184 (100 μM), (i) EP1 receptor antagonist, GW848687X (1 μM), (j) EP2 receptor antagonist, AH6809 (1 μM), (k) EP3 receptor antagonist, L-798,106 (10 μM), (l) EP4 receptor antagonist, AH23848 (10 μM), or equimolar concentration of the respective solvent (DMSO or Ethanol). N=3 per group. FGF6 and FGF9 were used at concentration of 200 ng/ml and 100 ng/ml, respectively. Data are presented as Means ± SEM. One-way ANOVA in b, d, and f. Two-way ANOVA in e, g-l. ****$p < 0.0001$, ***$p < 0.001$, **$p < 0.01$, *$p < 0.05$. A representative from a total of 2-3 independent experiments is shown. Source data are provided as a Source Data file.

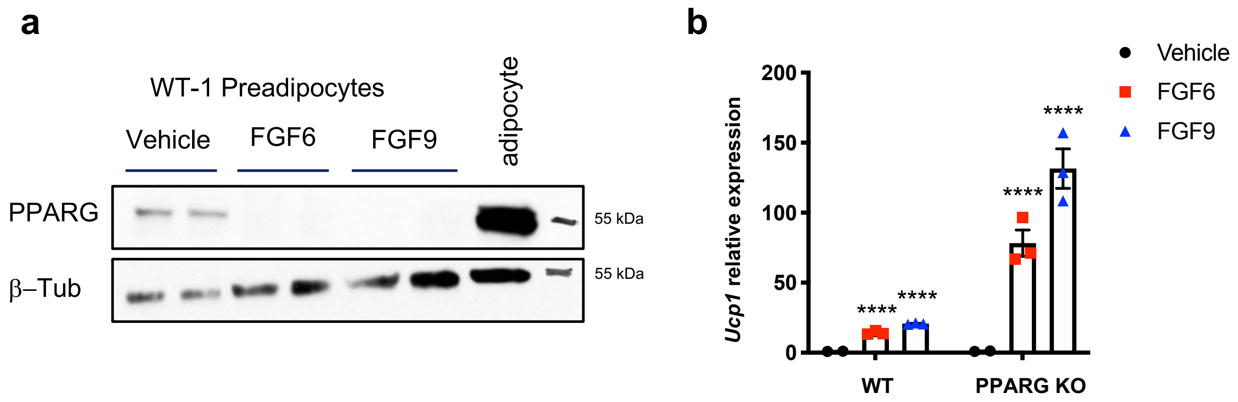

Supplementary Figure 9. FGF6/9 mediated induction of UCP1 is independent of PPARG, related to Figure 3. (a) PPARG protein level in brown preadipocytes treated with vehicle, FGF6, or FGF9 for 24 hours or differentiated adipocytes. 35 μg total protein was used for WB. (b) *Ucp1* expression in wildtype and Pparg KO brown MEFs treated with vehicle, FGF6, or FGF9 for 24 hours. Data are presented as Means ± SEM. Two-way ANOVA. ****p < 0.0001, ***p < 0.001, **p < 0.01. A representative from a total of 2 independent experiments is shown. Source data are provided as a Source Data file.

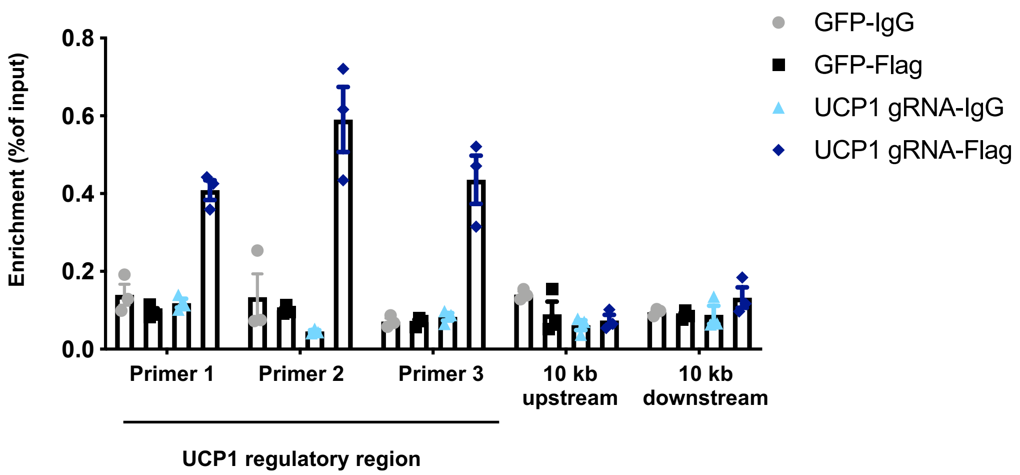

Supplementary Figure 10. Specific isolation of *Ucp1* regulatory region using enChIP, related to Figure 3. Enrichment of *Ucp1* regulatory region, but not upstream or downstream control regions by FLAG immunoprecipitation in cells transfected with UCP1 gRNAs. Source data are provided as a Source Data file.

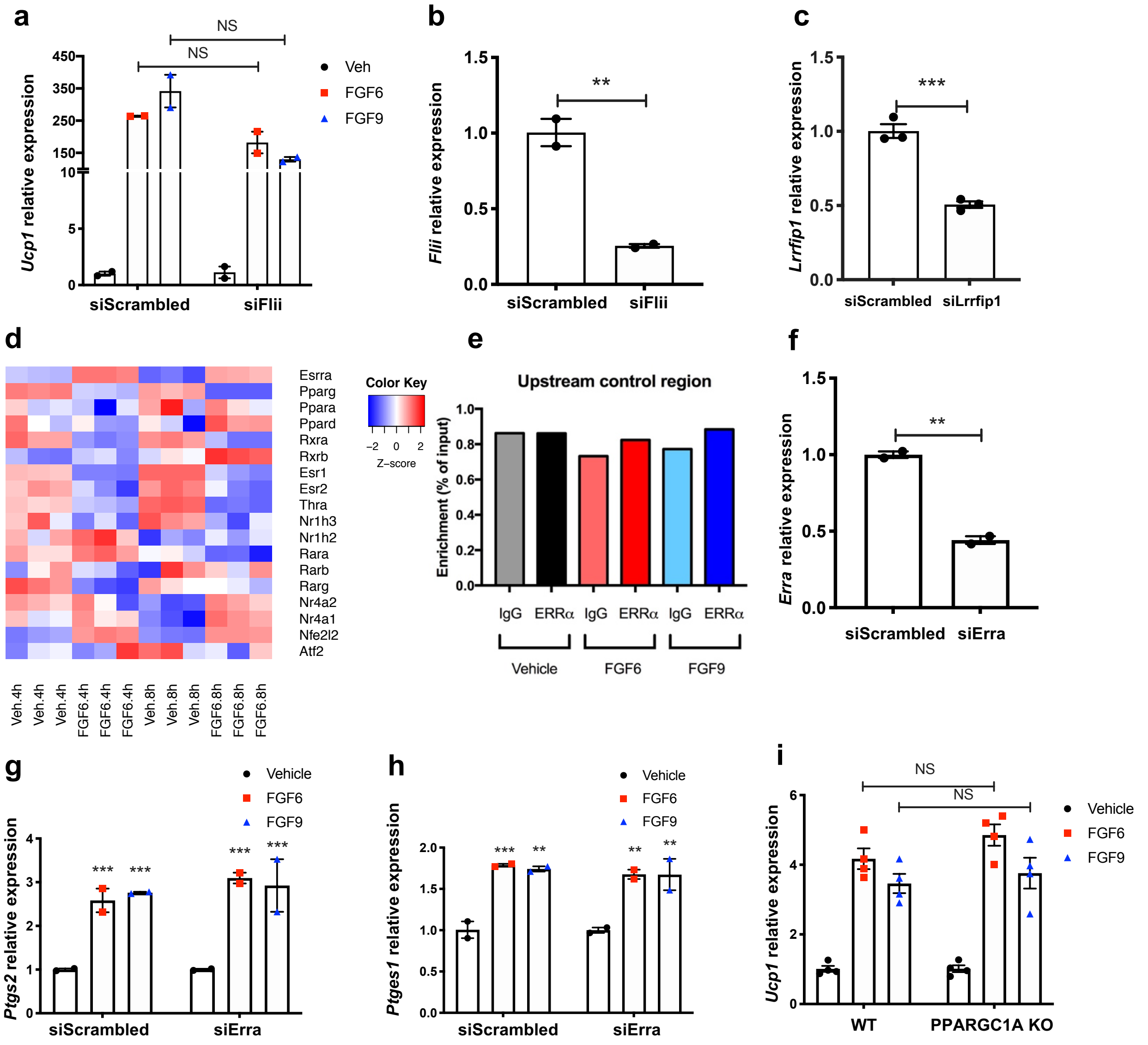

Supplementary Figure 11. Transcriptional regulation of UCP1 expression by ERRA/FLII/LRRFIP1 complex, related to Figure 3. (a) *Ucp1* expression in brown preadipocytes transfected with scrambled siRNA or si*Flii*, followed by treatment with vehicle, FGF6, or FGF9 for 24 hours. N=3 per group. (b) *Flii* mRNA expression in brown preadipocytes transfected with scrambled siRNA or si*Flii*. N=3 per group. (c) *Lrrfip1* mRNA expression in brown preadipocytes transfected with scrambled siRNA or si*Lrrfip1*. N=3 per group. (d) Heatmap showing the expression of nuclear receptors in brown preadipocytes treated with vehicle or FGF6 for 4 or 8 hours. (e) Chromatin immunoprecipitation of ERRA on a control region upstream of the *Ucp1* locus in brown preadipocytes treated with vehicle, FGF6, or FGF9 for 48 hours. (f) *Erra* expression in brown preadipocytes transfected with scrambled siRNA or si*Erra*. N=3 per group. (g) *Ptgs2*

and (h) *Ptges* expression in brown preadipocytes transfected with scrambled siRNA or si*Erra*, followed by treatment with vehicle, FGF6, or FGF9 for 24 hours. N=3 per group. (i) *Ucp1* expression in wildtype and *Ppargc1a* KO brown preadipocytes treated with vehicle, FGF6, or FGF9 for 24 hours. N=3 per group. N=3 per group. FGF6 and FGF9 were used at concentration of 200 ng/ml and 100 ng/ml, respectively. Data are presented as Means ± SEM. Two-way ANOVA in a and g-h, Two sample t-test in b-f ***p < 0.001, **p < 0.01, *p < 0.05. A representative from a total of 2-3 independent experiments is shown. Source data are provided as a Source Data file.

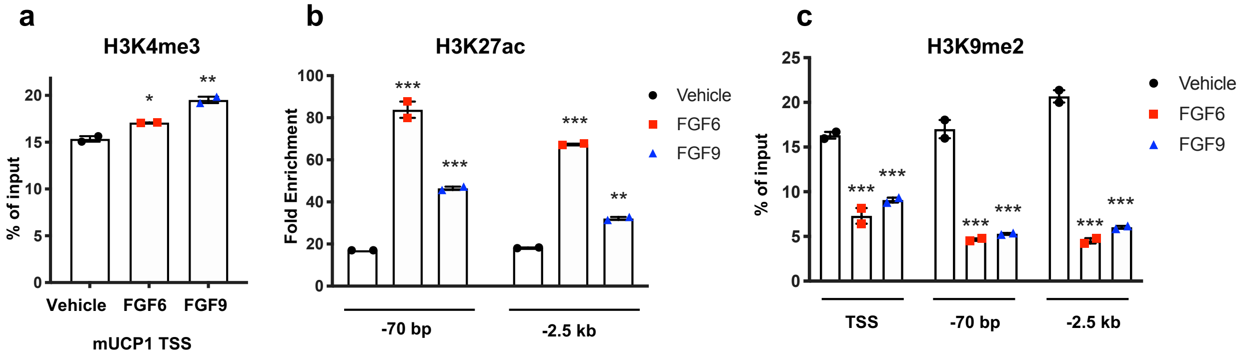

Supplementary Figure 12. FGF6 and FGF9 treatment results in epigenetic changes associated with transcriptional activation of *Ucp1,* related to Figure 3 (a) Chromatin immunoprecipitation for H3K4me3, (b) H3K27ac, and (c) H3K9me2 at the indicated sites in brown preadipocytes treated with vehicle, FGF6, or FGF9 for 24 hours. N=2 per group. Data are presented as Means ± SEM. One-way ANOVA in a and Two-way ANOVA in b-c. ***$p < 0.001$, **$p < 0.01$, *$p < 0.05$. A representative from a total of 2-3 independent experiments is shown. Source data are provided as a Source Data file.

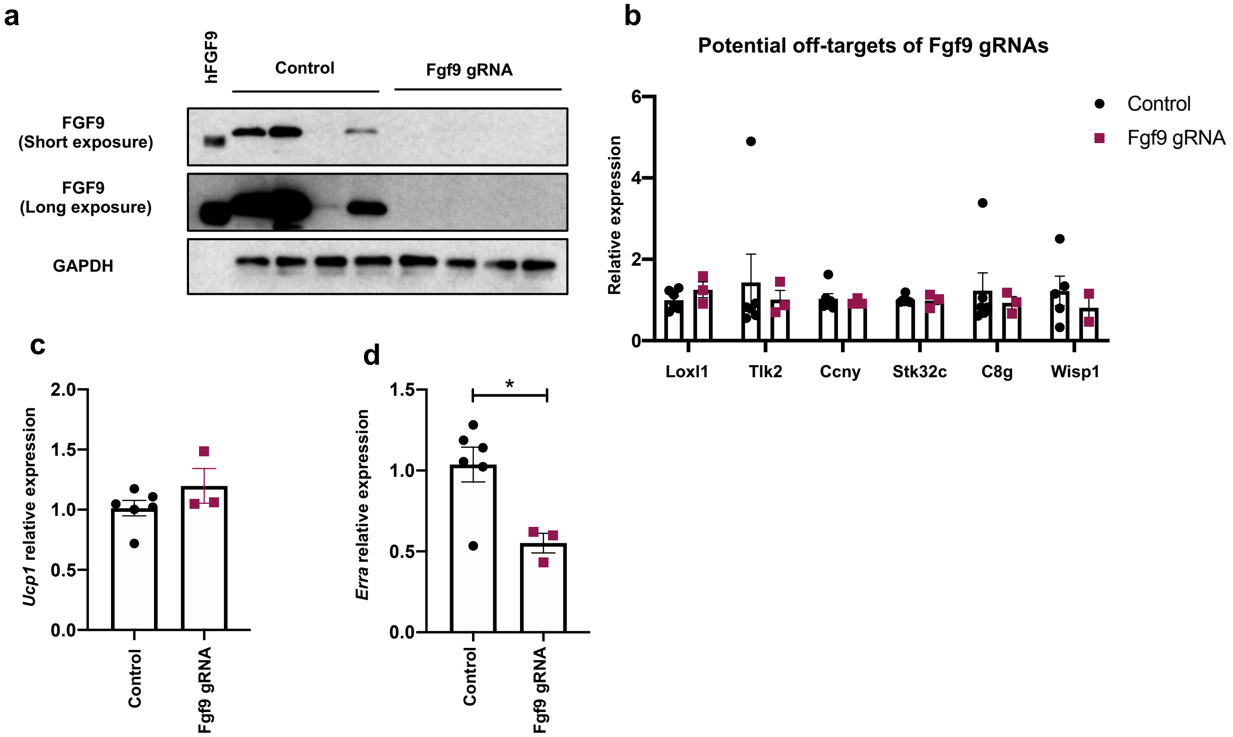

Supplementary Figure 13. FGF9 loss of function in BAT, related to Figure 5. (a) FGF9 protein in BAT of adiponectin-Cre Cas9 knock-in mice (Rosa26-floxed STOP-Cas9 knock-in) injected with control or AAV-FGF9 gRNA. 15 μg total protein was used for WB. (b) Gene expression of the predicted gRNA off-targets. N=3-6 per group. (c) *Ucp1* expression in BAT of Ucp1-Cre Cas9 knock-in mice injected with control or AAV-FGF9 gRNA upon 3 days cold exposure. (d) *Erra* expression in BAT-SVF derived from Ucp1-Cre Cas9 knock-in mice injected with control or AAV-FGF9 gRNA upon 3 days cold exposure. Data are presented as Means ± SEM. Two-way ANOVA in b and Two sample t-test in c and d. *p < 0.05. Source data are provided as a Source Data file.

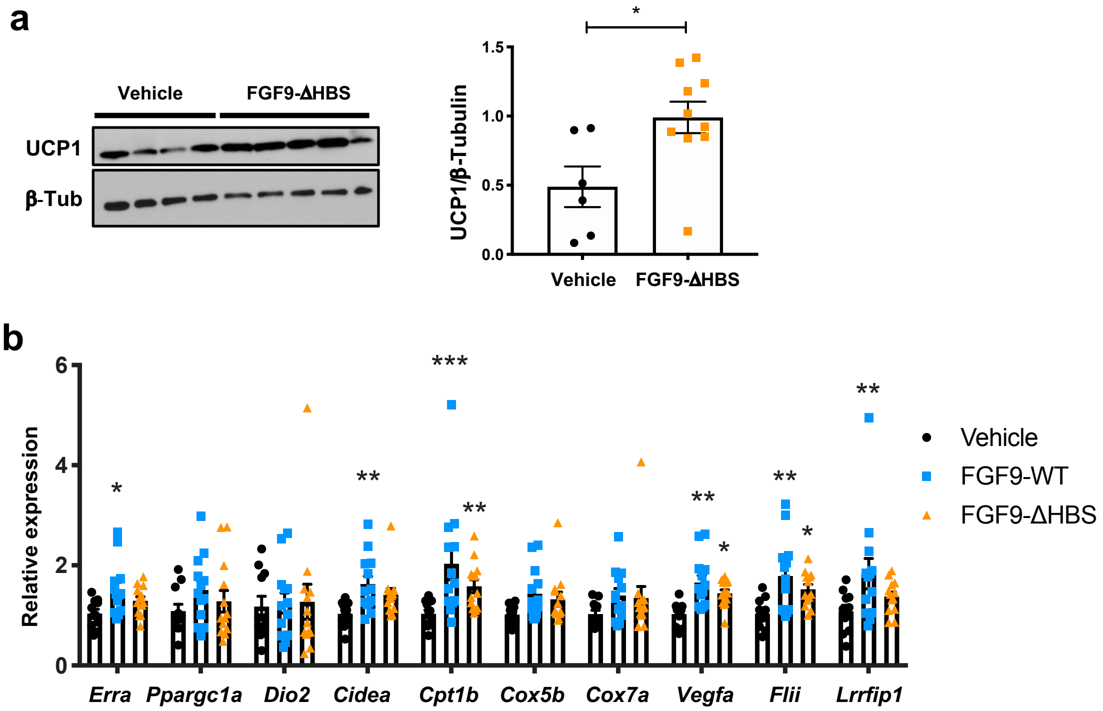

Supplementary Figure 14. FGF9 protein induces UCP1 and other thermogenic genes in BAT, related to Figure 5. (a) UCP1 protein levels in BAT after 17 days of daily subcutaneous injection of FGF9-ΔHBS or vehicle. N=6-10 per group. 12.5 μg total protein was used for WB. (b) Gene expression in BAT after 17 days of daily subcutaneous injection of FGF9-WT, FGF9-ΔHBS, or vehicle. N=12-13 per group. Data are presented as Means ± SEM. Two sample t-test in a and Two-way ANOVA in b. ***$p < 0.001$, **$p < 0.01$, *$p < 0.05$. A representative from a total of 2-3 independent experiments is shown. Source data are provided as a Source Data file.

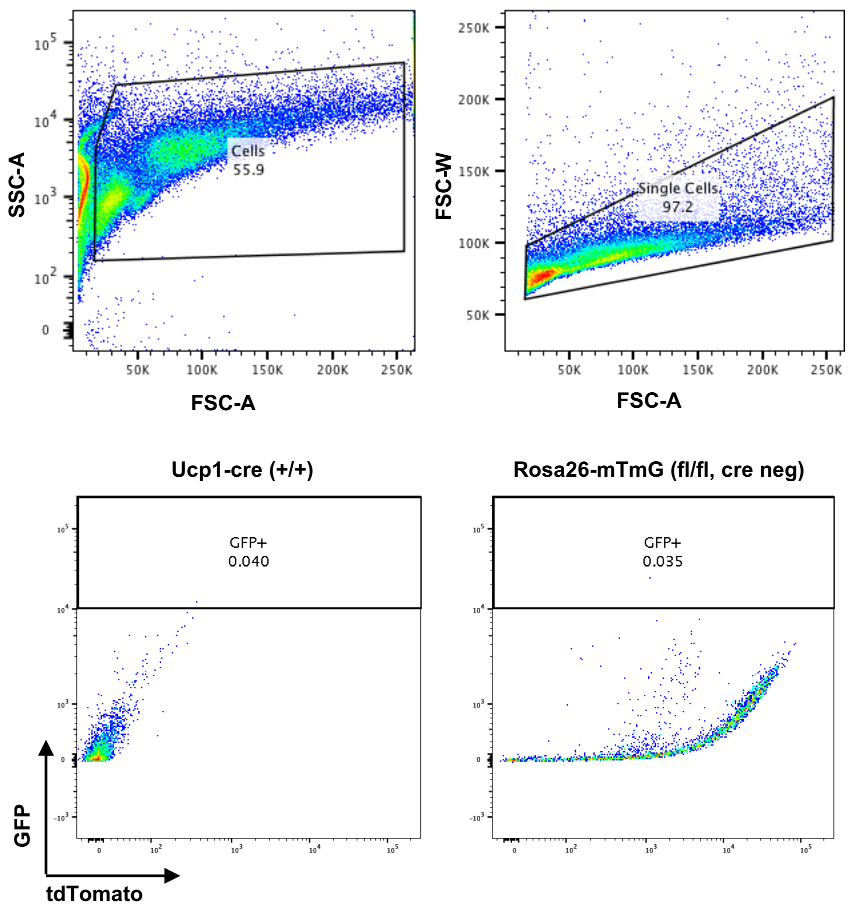

Supplementary Figure 15. Gating strategy in flow cytometry analysis in BAT-SVF of Ucp1-cre Rosa26-mTmG, related to Figure 5. Top: Debris and doublets were excluded based on forward and side scatter profiles. Bottom: tdTomato and GFP labeling in samples with neither tdTomato or GFP labeling (Ucp1-cre only) on the left and tdTomato labeling (Rosa26-mTmG only) on the right.

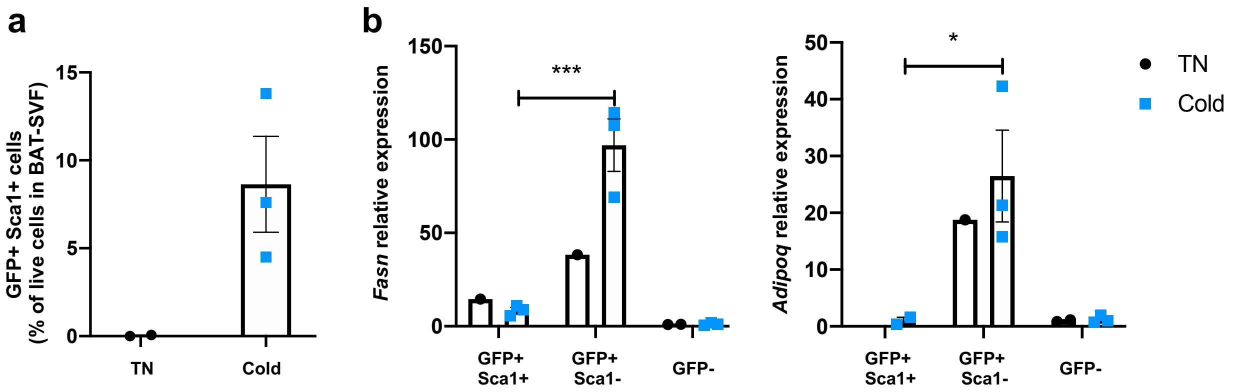

Supplementary Figure 16. Characterization of GFP+ Sca-1+ cells in BAT-SVF, related to Figure 5. (a) Frequency of GFP+ Sca-1+ cells and (b) *Fasn* and *Adipoq* expression in GFP+ Sca-1+, GFP+ Sca-1-, and GFP- cells isolated from the BAT-SVF of Ucp1-cre Rosa26-mTmG mice housed at TN or cold for 7 days. N=2-3 per group. Data are presented as Means ± SEM. Two sample t-test in b. ***$p < 0.001$, *$p < 0.05$. A representative from a total of 2 independent experiments is shown. Source data are provided as a Source Data file.

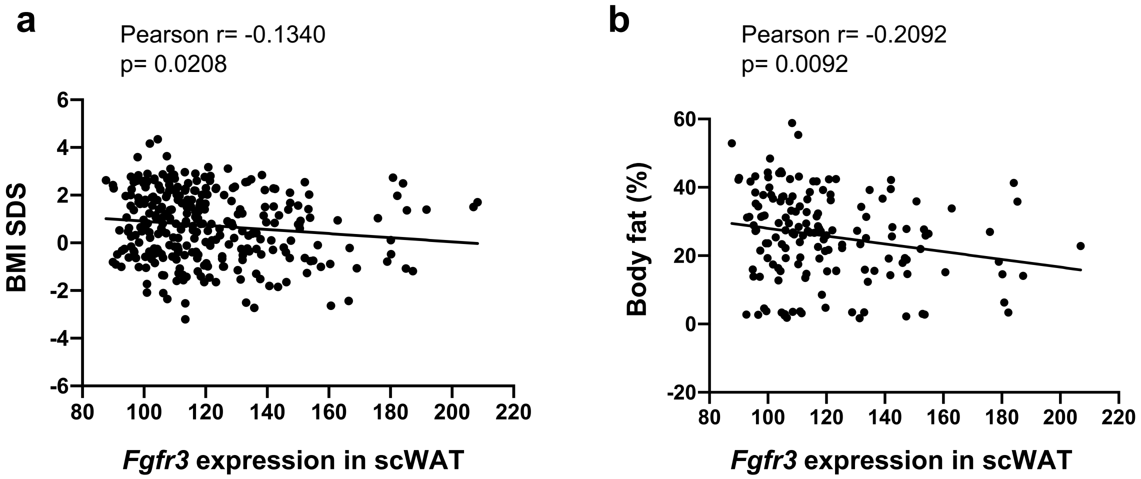

Supplementary Figure 17. Correlations in childhood cohort, related to Figure 5. Correlation between *Fgfr3* expression in scWAT and (a) BMI (n=297) and (b) percentage of body fat (n= 154) in childhood cohort. Pearson correlation coefficient and p-values are shown. Source data are provided as a Source Data file.

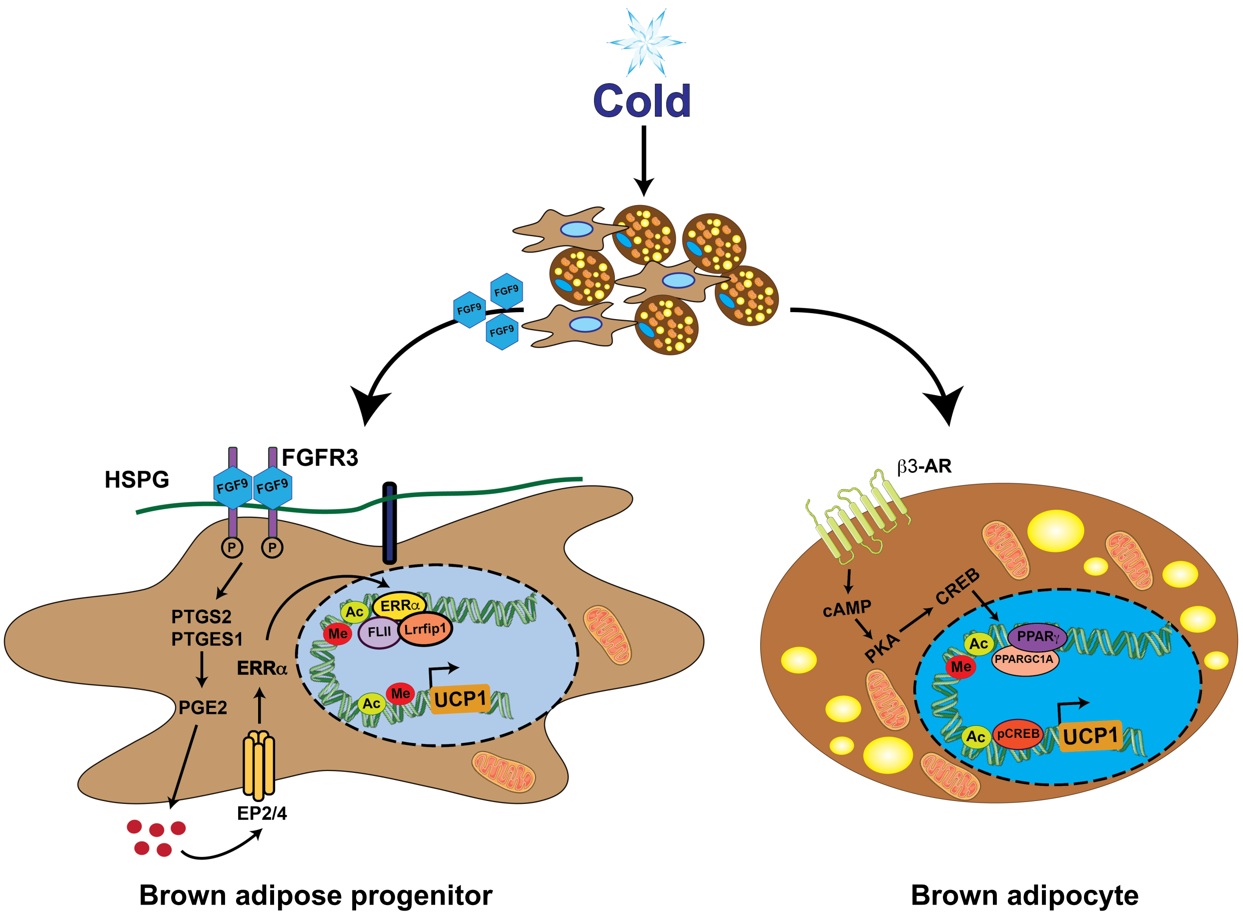

Supplementary Figure 18. Regulation of UCP1 expression by canonical and non-canonical pathways. Cold exposure triggers production of catecholamines which activate brown adipocytes mainly through activation of the β3-adrenergic receptor (β3-AR). Activation of β3-AR results in rise of cAMP. cAMP-dependent protein kinase (PKA) transduces the cAMP signal to its downstream targets including cAMP response element-binding protein, CREB. Phosphorylated CREB activates Ucp1 expression directly (through binding to CRE elements in Ucp1 proximal promoter) and indirectly (through inducing the expression of peroxisome proliferator-activated receptor gamma coactivator 1-alpha (PPARGC1A). Cold exposure also elevates FGF9 expression in BAT. Binding of FGF9 to FGFR3 induces PGE2 production through induction of the key enzymes involved in its biosynthesis. In the next step, PGE2 molecules act through EP2 and EP4 cell surface receptors to promote the binding of ERRA to ERR response element in the *Ucp1* enhancer. ERRA binding, together with the recruitment of its transcriptional co-activators FLII/LRRFIP1 to the *Ucp1* locus enables transcriptional activation.
