# Supplementary material for: FGF6 and FGF9 regulate UCP1 expression independent of brown adipogenesis
Source: Nat Commun. 2020 Mar 17;11:1421. doi: 10.1038/s41467-020-15055-9 (PMC7078224; doi:10.1038/s41467-020-15055-9)
Supplement: Supplementary file 2 — Description of Additional Supplementary Information [file 41467_2020_15055_MOESM2_ESM.pdf]

Description of Additional Supplementary Files

File Name: Supplementary Data 1
Description: The Supplementary Data 1 contains the analyzed RNA-sequencing data of mouse brown preadipocytes treated with FGF6 (200 ng/ml) or vehicle for 4, 8, and 24 hours.

File Name: Supplementary Data 2
Description: The Supplementary Data 2 contains the list of proteins identified in the enChIP experiment.

File Name: Supplementary Data 3
Description: The Supplementary Data 3 contains the sequences of primers used in the study.

File Name: Supplementary Data 4
Description: The Supplementary Data 4 contains the original uncropped scans of the western blots.
