# Supplementary material for: FGF6 and FGF9 regulate UCP1 expression independent of brown adipogenesis
Source: Nat Commun. 2020 Mar 17;11:1421. doi: 10.1038/s41467-020-15055-9 (PMC7078224; doi:10.1038/s41467-020-15055-9)

Original blots related to Figure 1e

UCP1

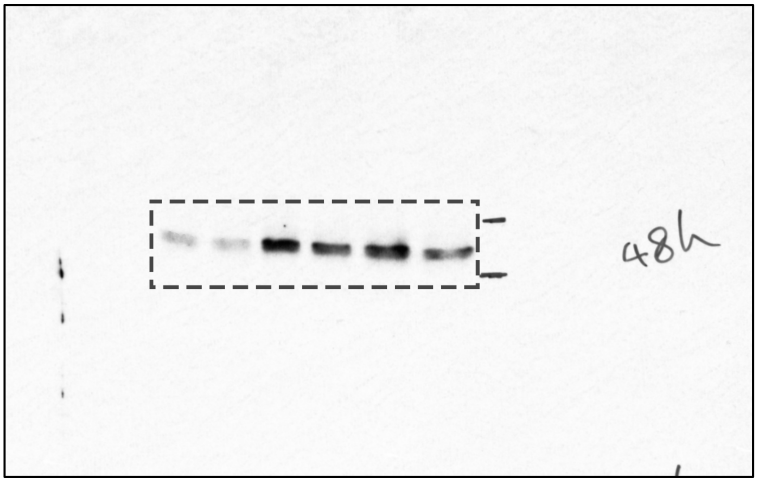

β-Tubulin

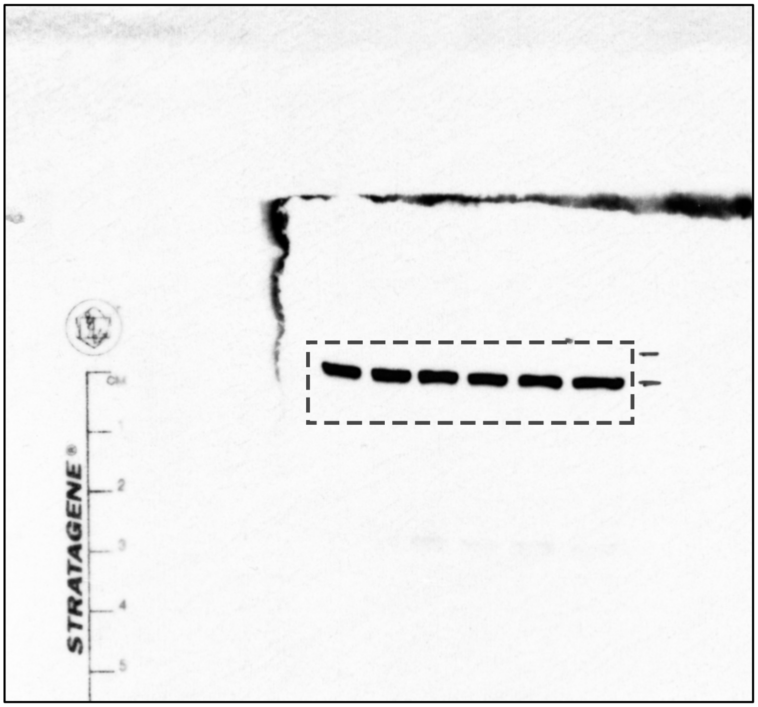

Original blots related to Figure 3e

FLII

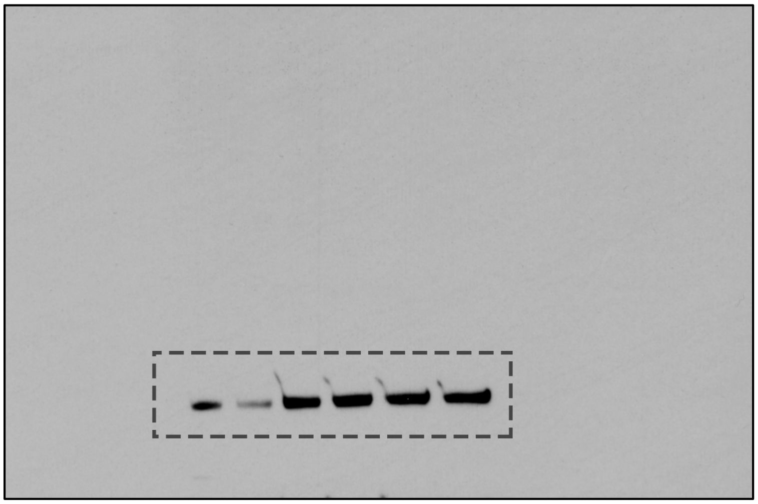

β-Tubulin

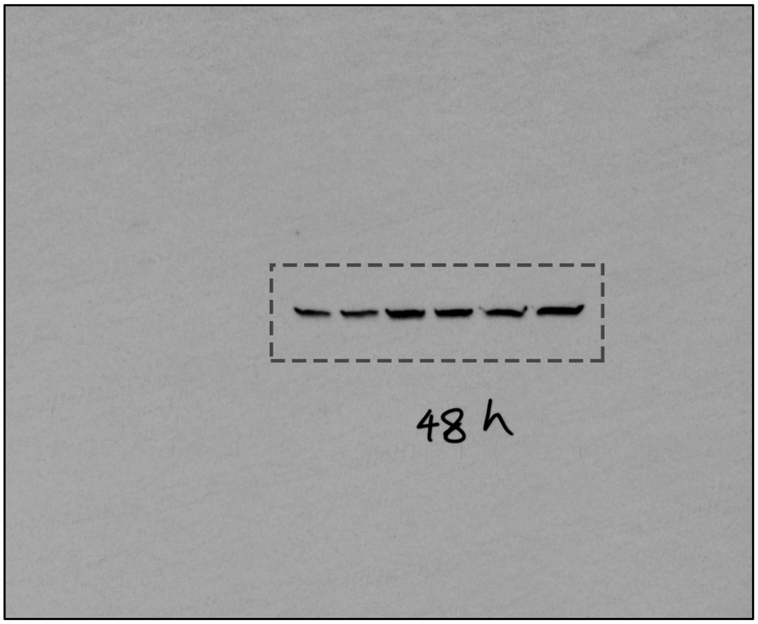

Original blots related to Figure 3g

ERRA

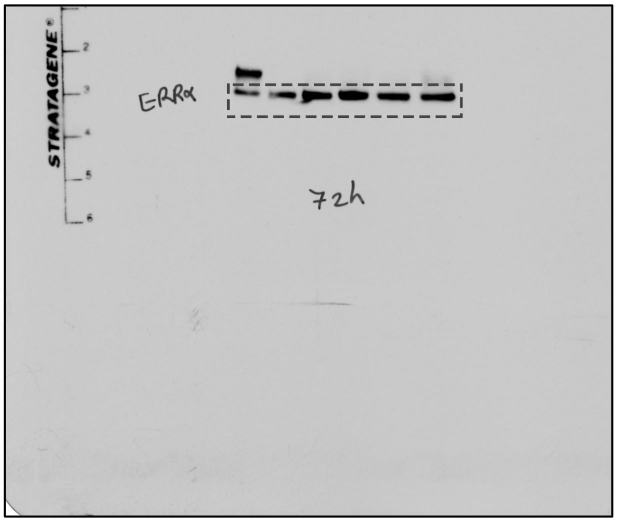

β-Tubulin

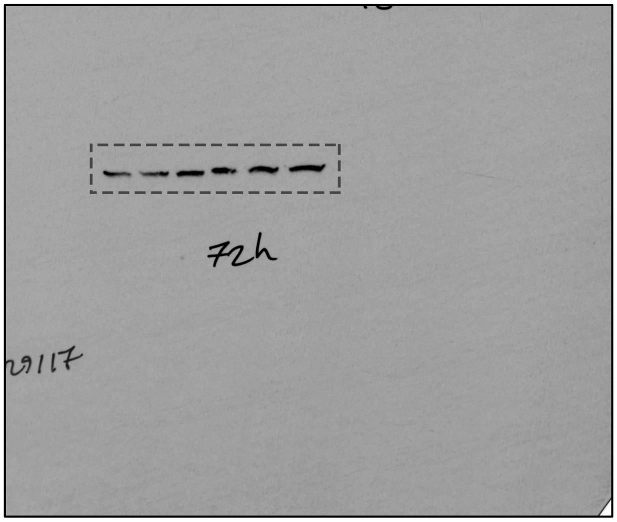

Original blots related to Figure 3I

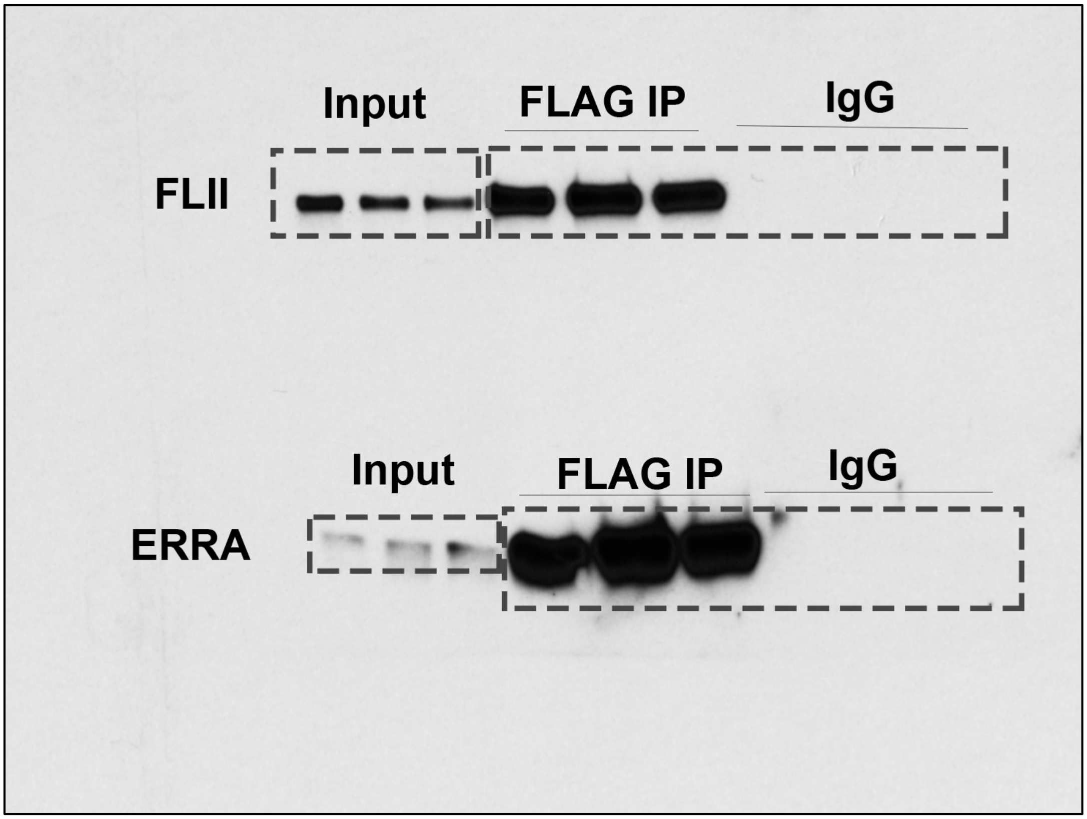

Original blots related to Figure 4h

ERRA

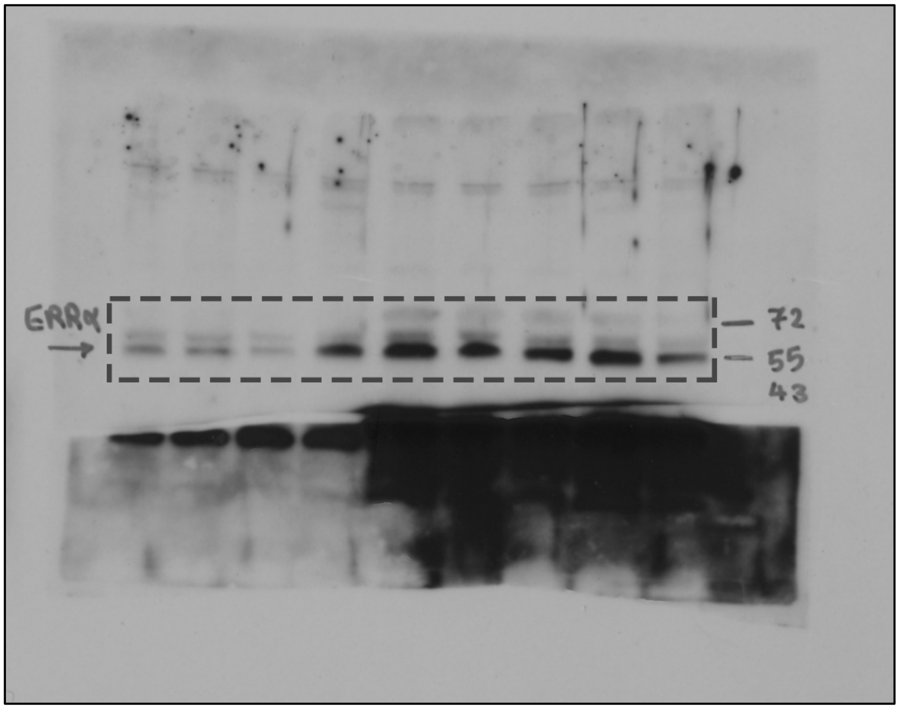

β-Tubulin

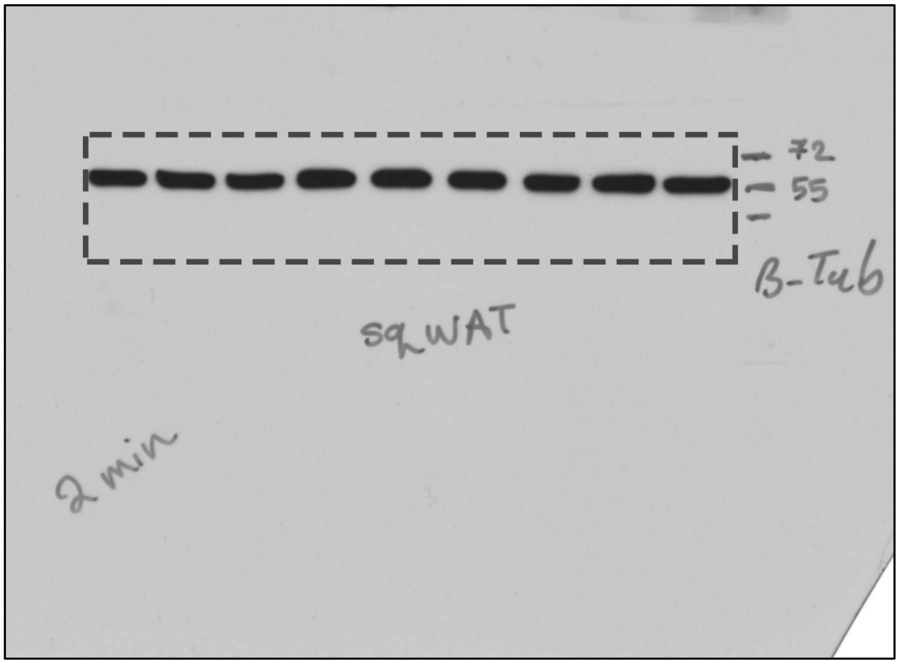

Original blots related to Figure 4i

FLII

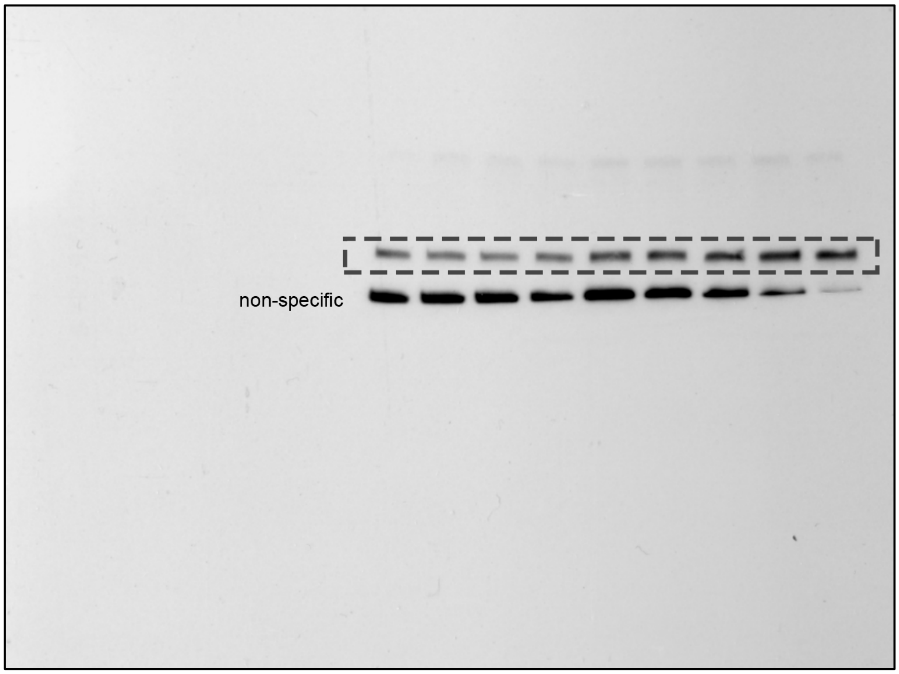

β-Tubulin

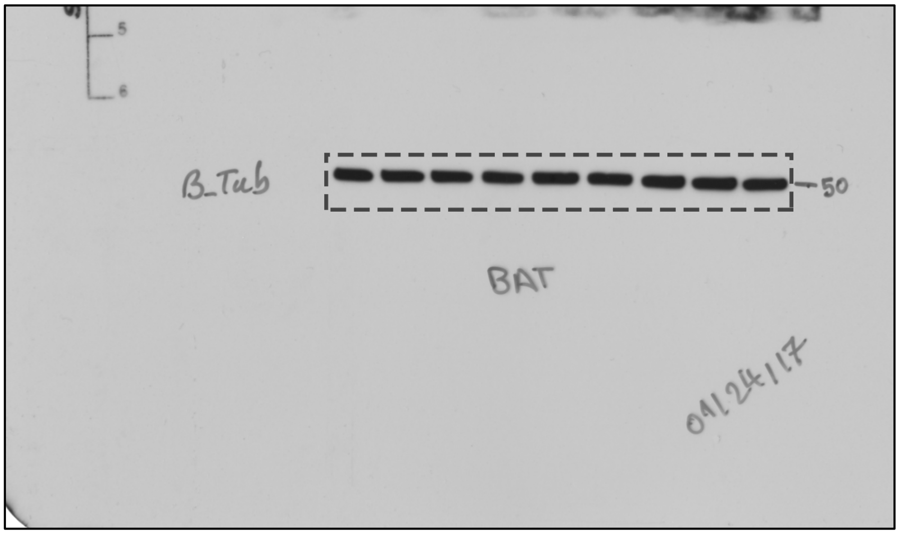

Original blots related to Extended Data Figure 1h

UCP1

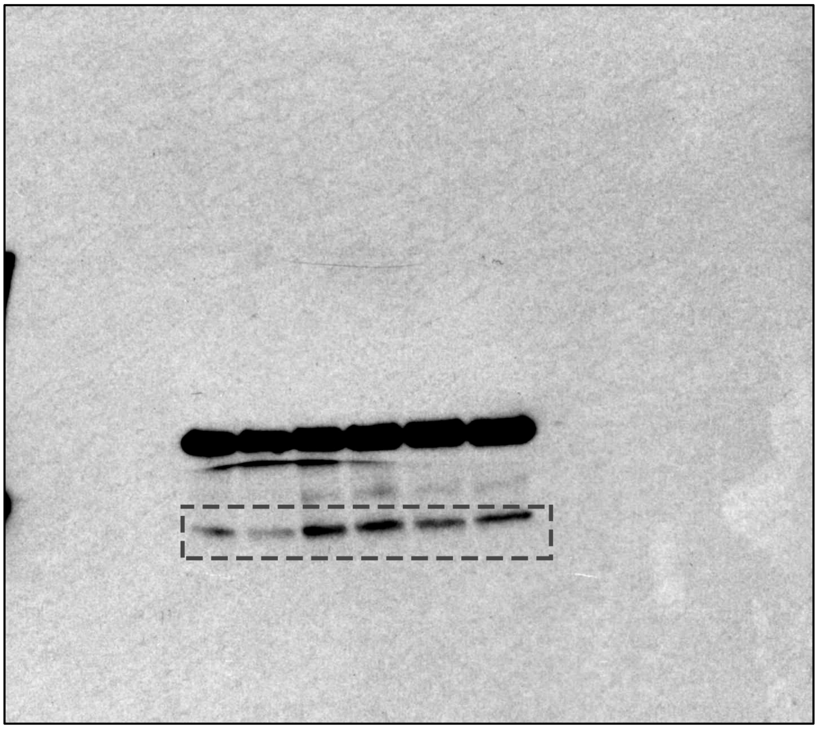

β-Tubulin

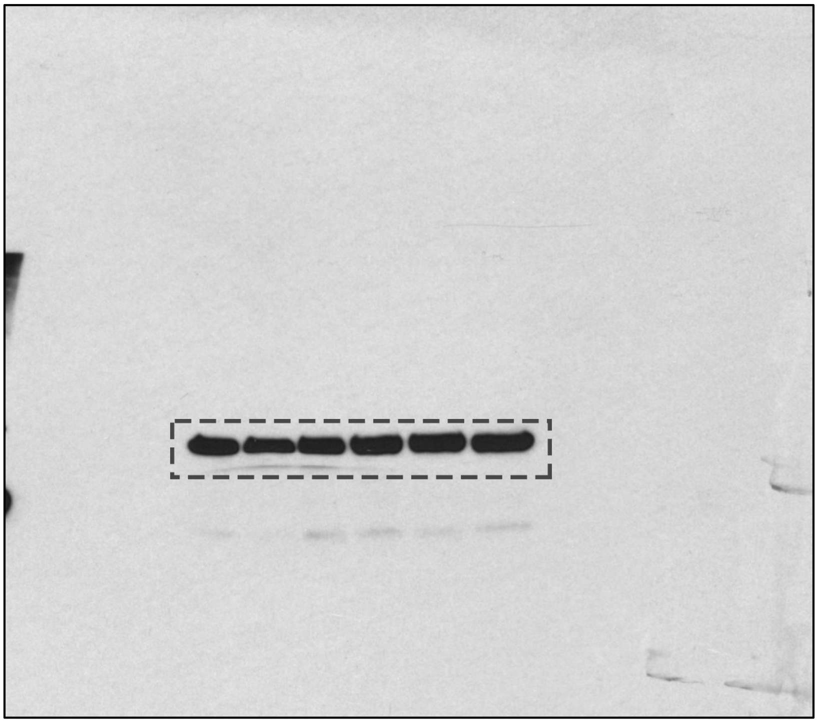

Original blots related to Figure 2c

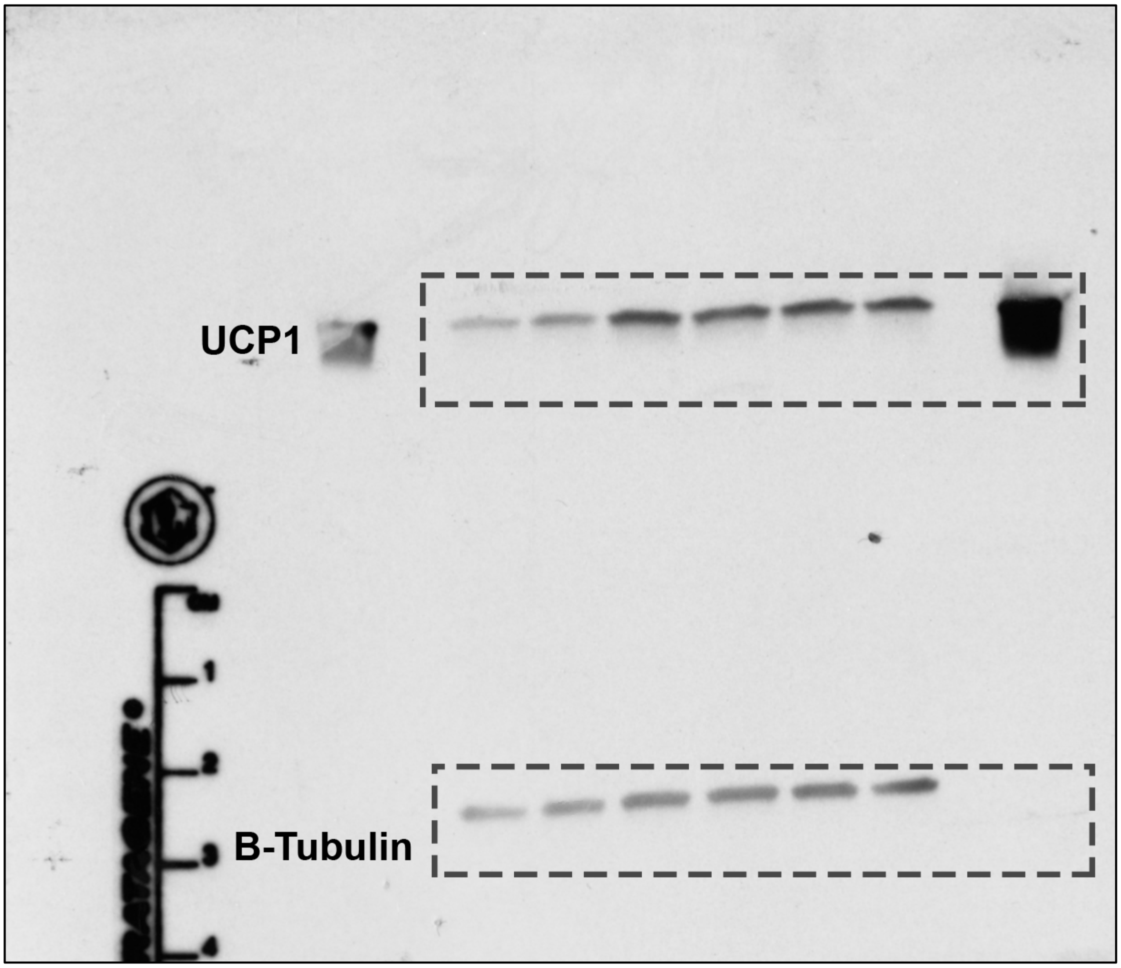

Original blots related to Extended Data Figure 2f

UCP1

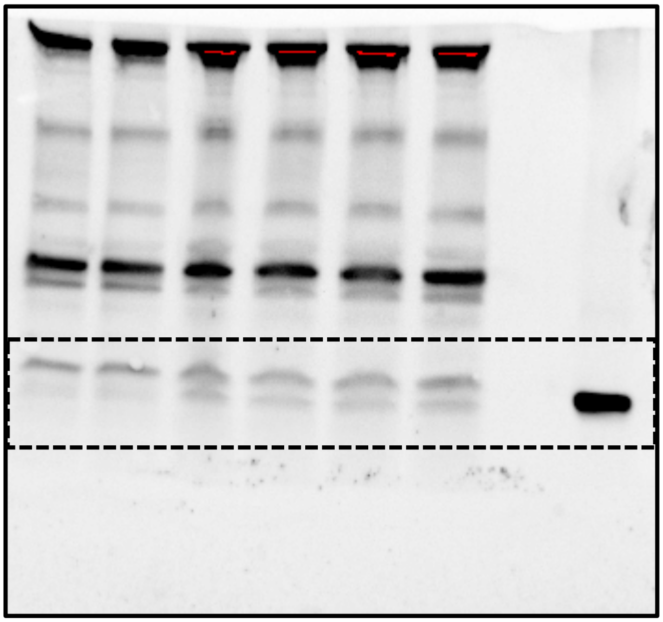

β-Tubulin

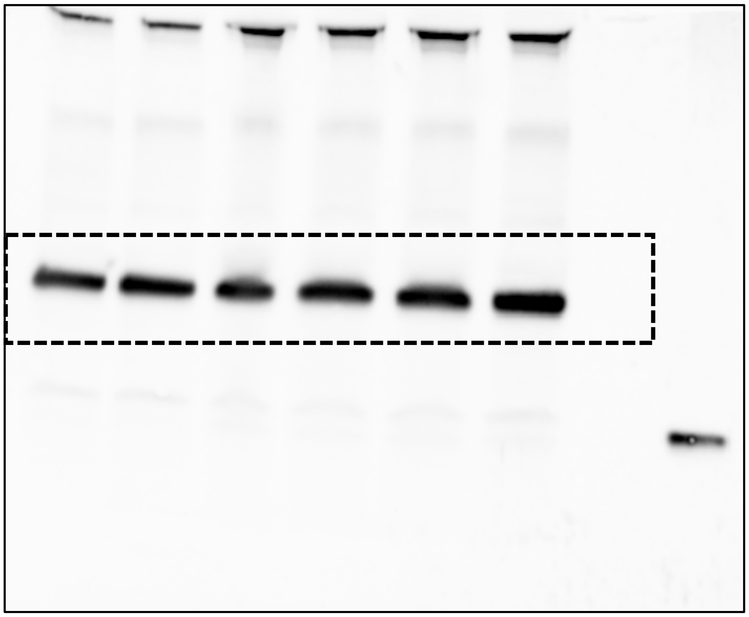

Original blots related to Extended Data Figure 4c

UCP1

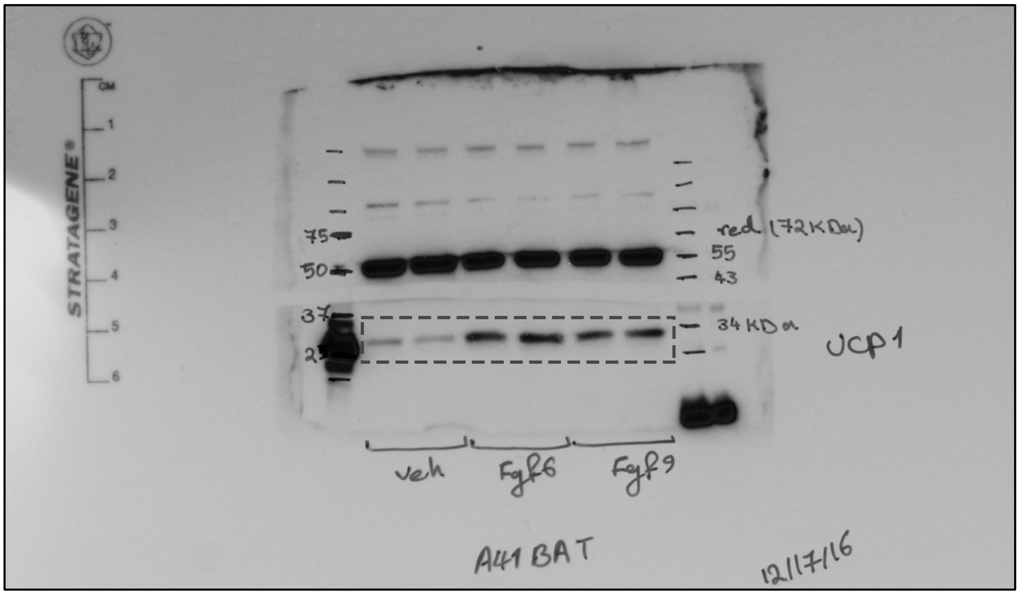

β-Tubulin

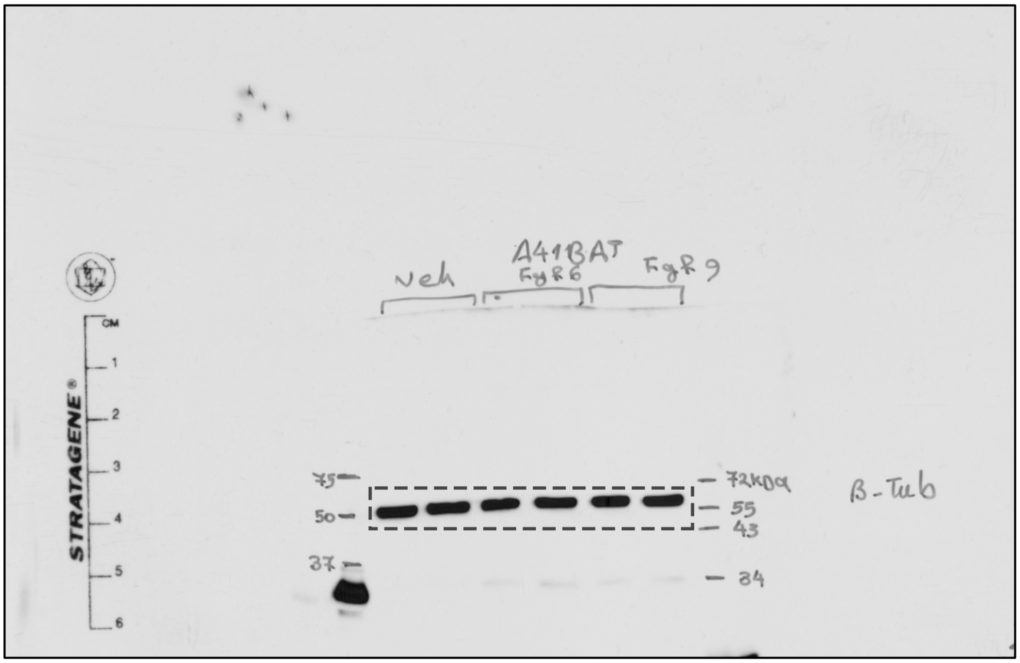

Original blots related to Extended Data Figure 4f

UCP1

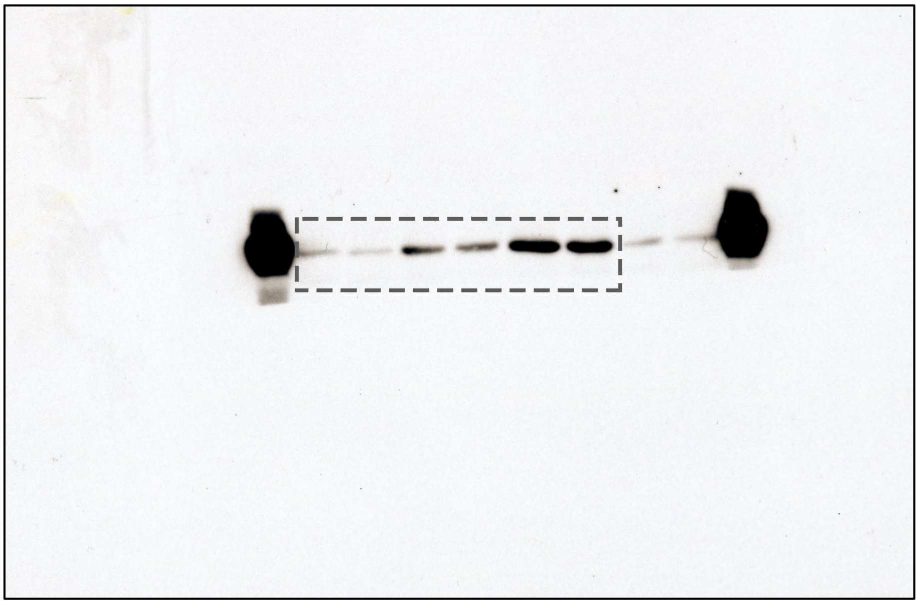

β-Tubulin

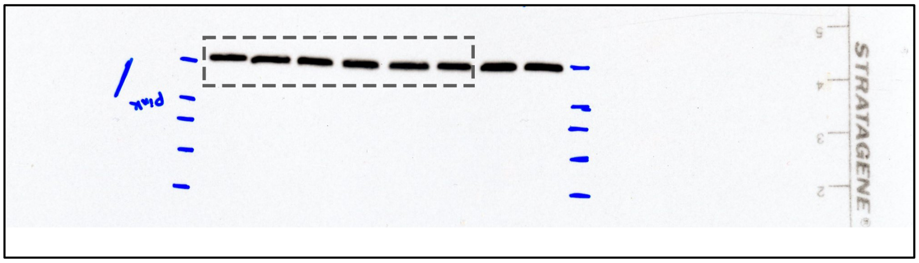

Original blots related to Extended Data Figure 7d

FGFR1

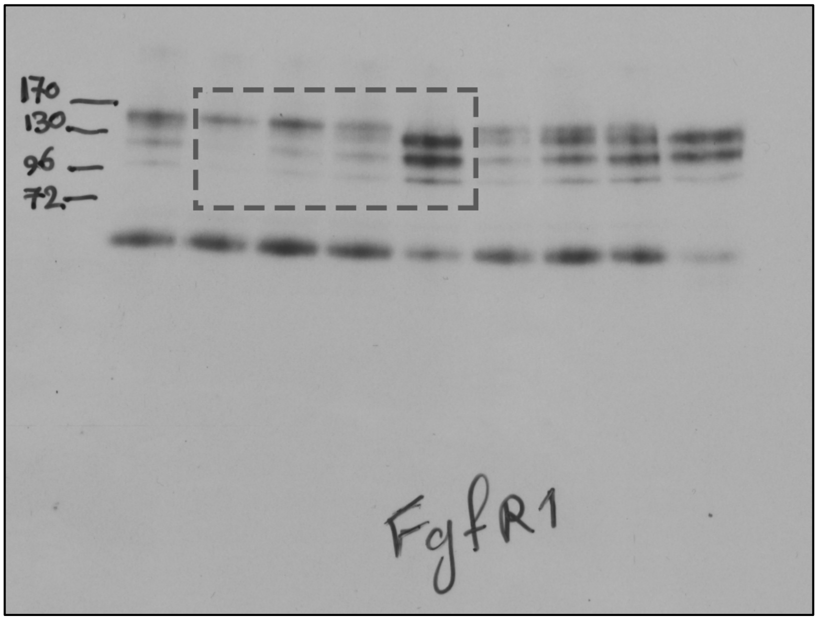

β-Tubulin

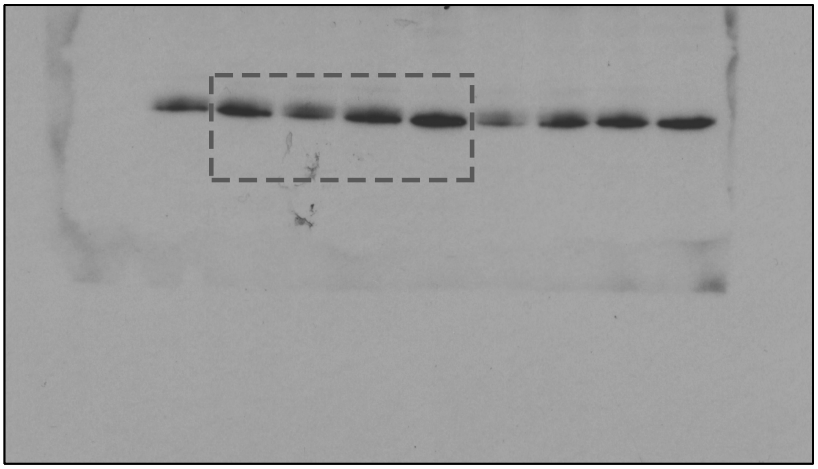

Original blots related to Extended Data Figure 8c

PTGS2

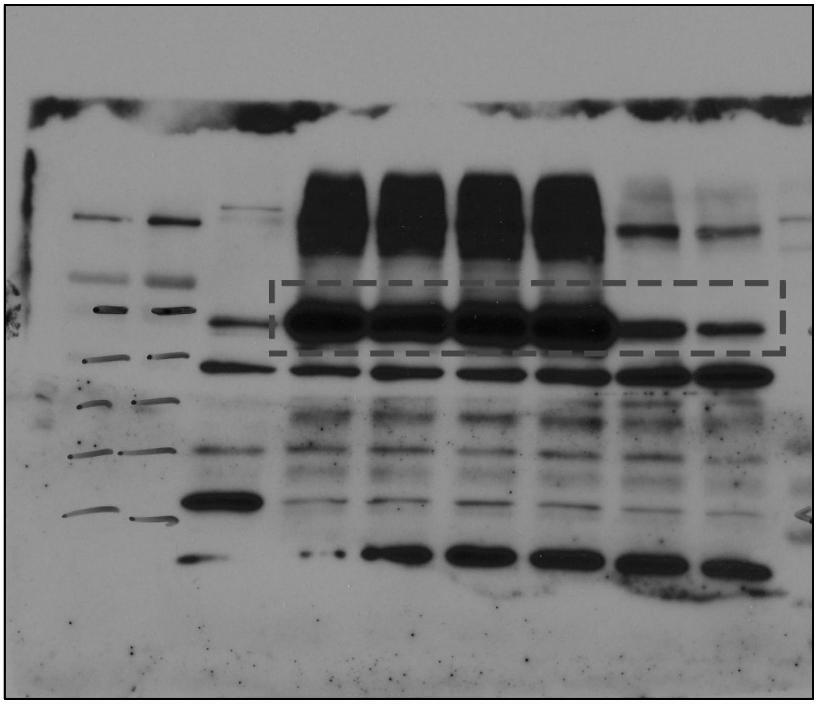

β-Tubulin

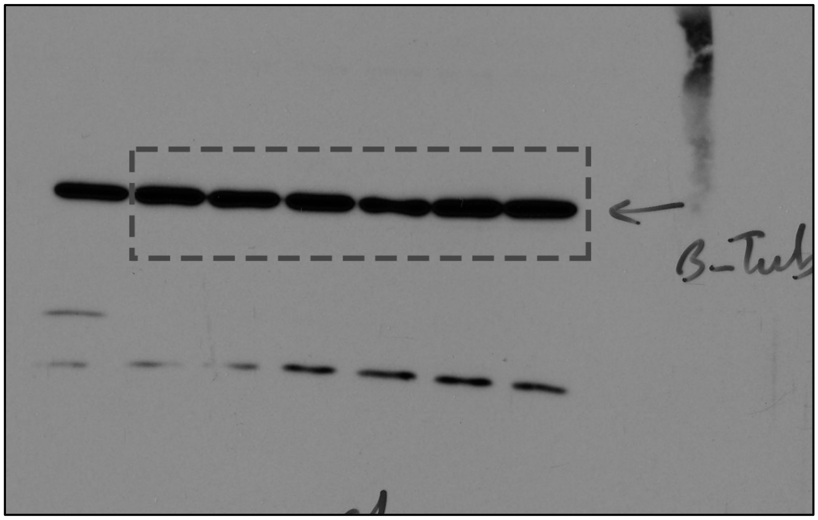

Original blots related to Extended Data Figure 9a

PPARG

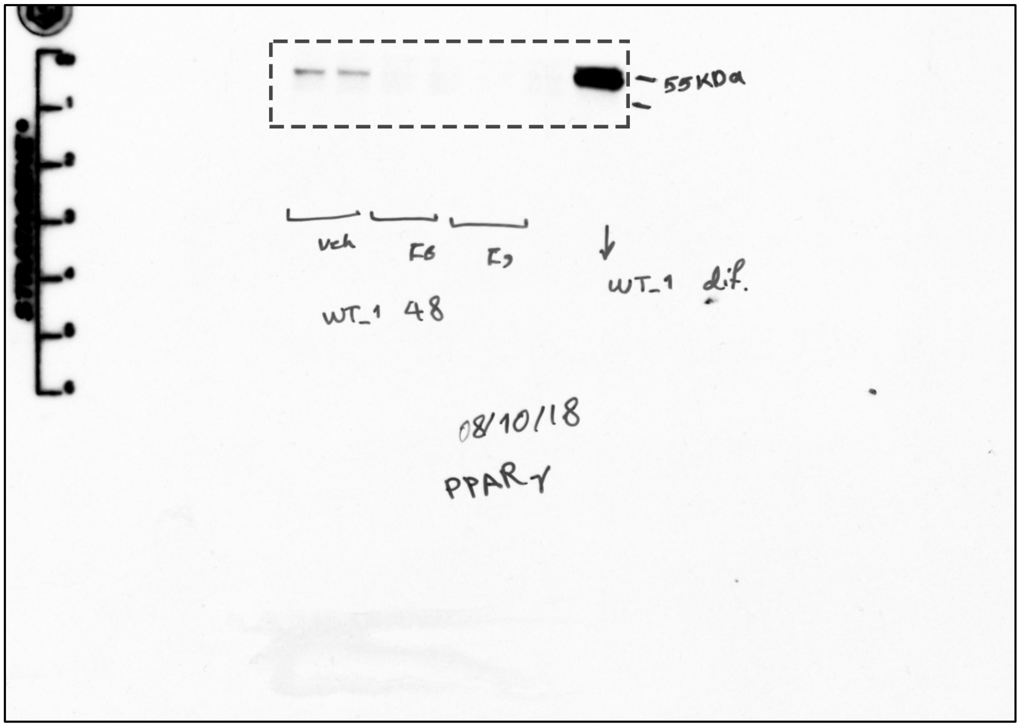

β-Tubulin

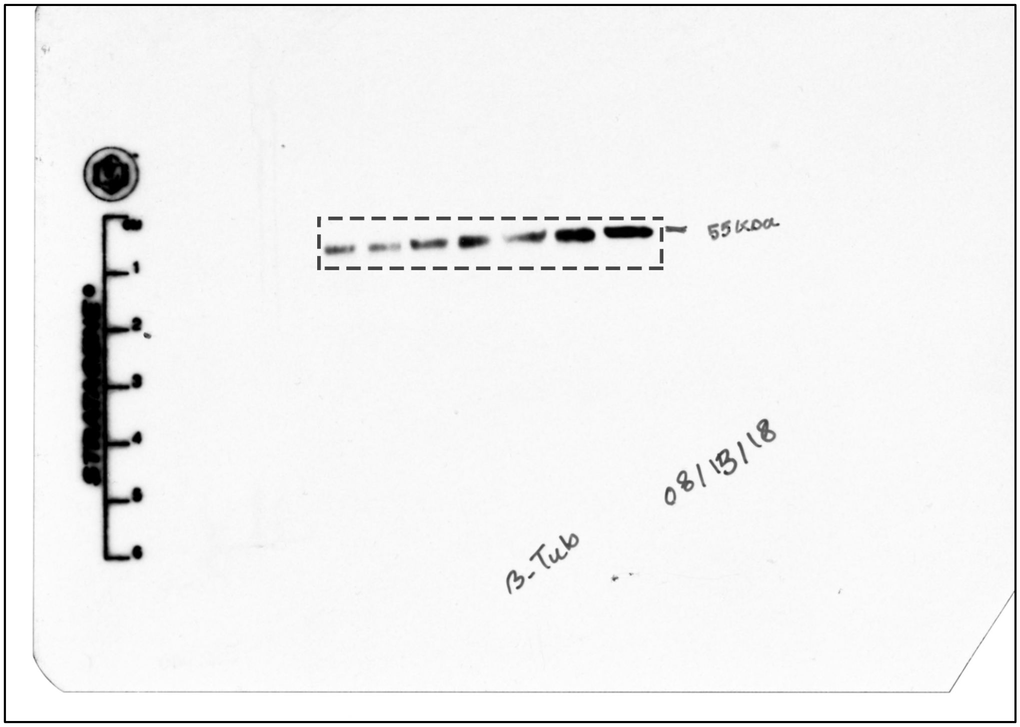

Original blots related to Extended Data Figure 13a

FGF9 (short exposure)

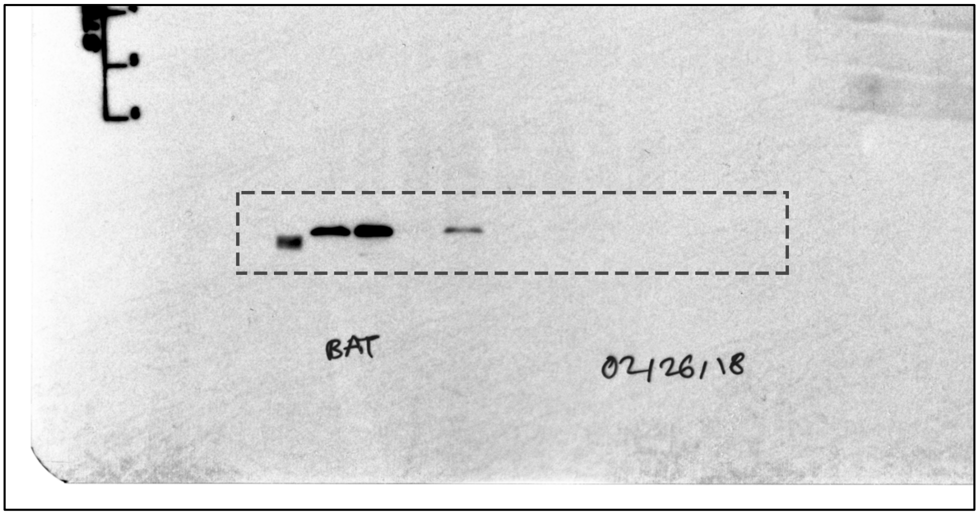

FGF9 (long exposure)

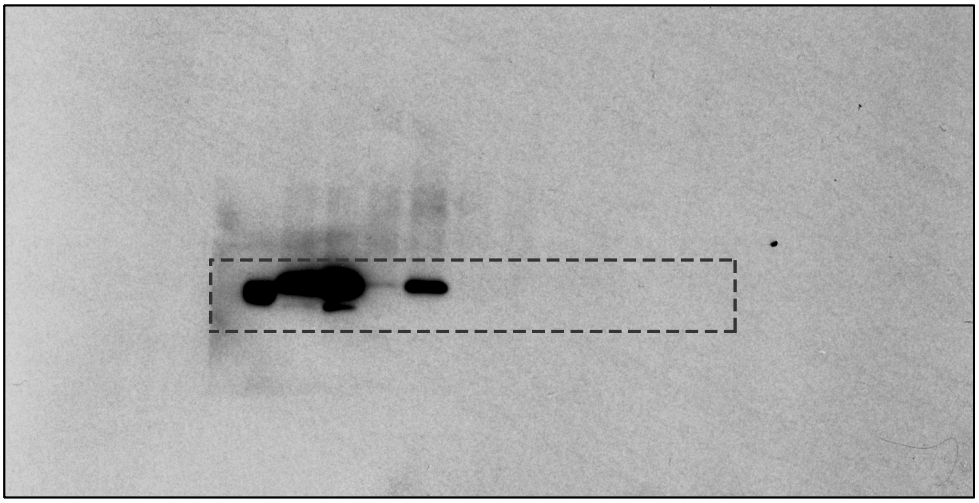

GAPDH

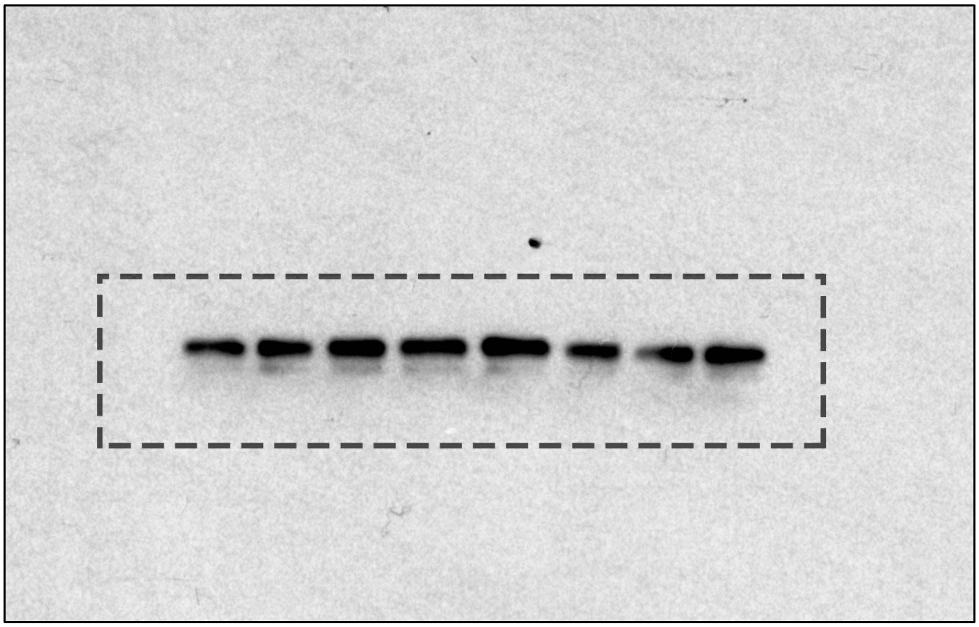

Original blots related to Figure 14a

UCP1

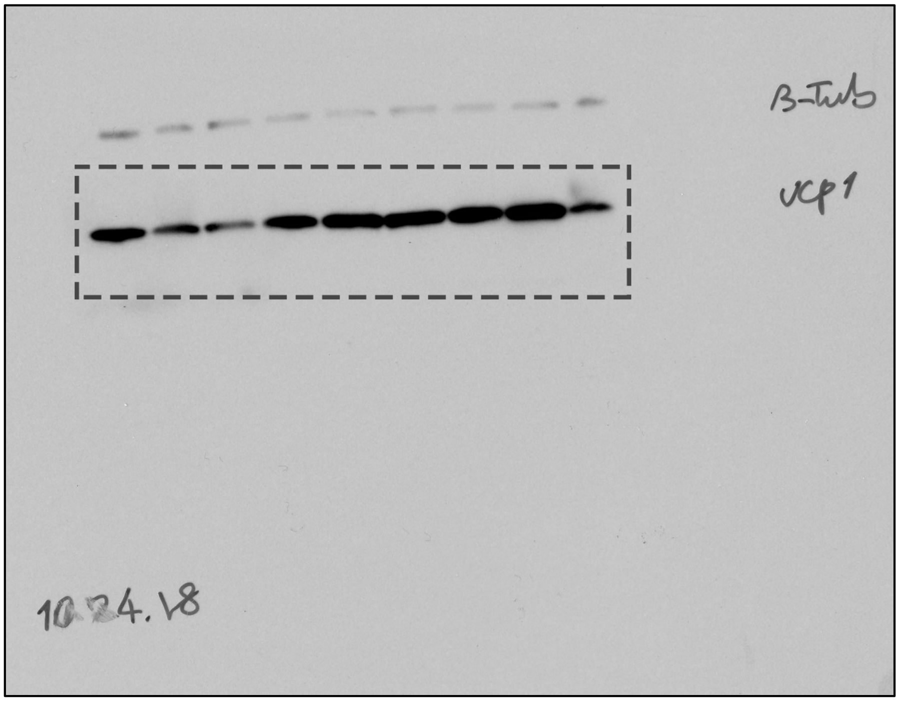

β-Tubulin

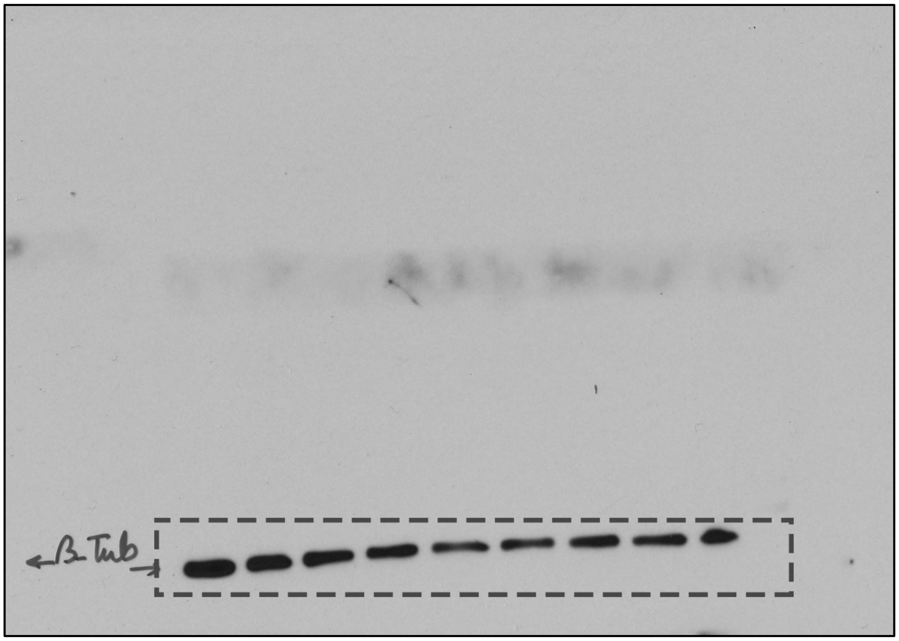

Supplement: Supplementary file 6 — Supplementary Dataset 4 [file 41467_2020_15055_MOESM6_ESM.pdf]
