# Supplementary material for: FGF6 and FGF9 regulate UCP1 expression independent of brown adipogenesis
Source: Nat Commun. 2020 Mar 17;11:1421. doi: 10.1038/s41467-020-15055-9 (PMC7078224; doi:10.1038/s41467-020-15055-9)
Supplement: Supplementary file 7 — Reporting Summary [file 41467_2020_15055_MOESM7_ESM.pdf]

nature research

Corresponding author(s): Yu-Hua Tseng

Last updated by author(s): 01/24/2020

Reporting Summary

Nature Research wishes to improve the reproducibility of the work that we publish. This form provides structure for consistency and transparency in reporting. For further information on Nature Research policies, see Authors & Referees and the Editorial Policy Checklist .

Statistics

For all statistical analyses, confirm that the following items are present in the figure legend, table legend, main text, or Methods section.

n/a	Confirmed	
☐	☒	The exact sample size (*n*) for each experimental group/condition, given as a discrete number and unit of measurement
☐	☒	A statement on whether measurements were taken from distinct samples or whether the same sample was measured repeatedly
☐	☒	The statistical test(s) used AND whether they are one- or two-sided<br>*Only common tests should be described solely by name; describe more complex techniques in the Methods section.*
☐	☒	A description of all covariates tested
☐	☒	A description of any assumptions or corrections, such as tests of normality and adjustment for multiple comparisons
☐	☒	A full description of the statistical parameters including central tendency (e.g. means) or other basic estimates (e.g. regression coefficient) AND variation (e.g. standard deviation) or associated estimates of uncertainty (e.g. confidence intervals)
☐	☒	For null hypothesis testing, the test statistic (e.g. *F*, *t*, *r*) with confidence intervals, effect sizes, degrees of freedom and *P* value noted<br>*Give P values as exact values whenever suitable.*
☒	☐	For Bayesian analysis, information on the choice of priors and Markov chain Monte Carlo settings
☒	☐	For hierarchical and complex designs, identification of the appropriate level for tests and full reporting of outcomes
☒	☐	Estimates of effect sizes (e.g. Cohen's *d*, Pearson's *r*), indicating how they were calculated

Our web collection on statistics for biologists contains articles on many of the points above.

Software and code

Policy information about availability of computer code

Data collection	Flow cytometry data were collected using DIVA (Becton Dickinson) software.
Data analysis	All statistics were calculated using Microsoft Excel, Graphpad Prism, and RStudio using LIMMA package.

For manuscripts utilizing custom algorithms or software that are central to the research but not yet described in published literature, software must be made available to editors/reviewers. We strongly encourage code deposition in a community repository (e.g. GitHub). See the Nature Research guidelines for submitting code & software for further information.

Data

Policy information about availability of data

All manuscripts must include a data availability statement. This statement should provide the following information, where applicable:

- Accession codes, unique identifiers, or web links for publicly available datasets
- A list of figures that have associated raw data
- A description of any restrictions on data availability

RNA-sequencing data were deposited in the Gene Expression Omnibus (GEO accession # GSE144061).

Field-specific reporting

Please select the one below that is the best fit for your research. If you are not sure, read the appropriate sections before making your selection.

☒ Life sciences ☐ Behavioural & social sciences ☐ Ecological, evolutionary & environmental sciences

For a reference copy of the document with all sections, see nature.com/documents/nr-reporting-summary-flat.pdf

Life sciences study design

All studies must disclose on these points even when the disclosure is negative.

Sample size	No statistical method was used to predetermine sample size.<br>For animal experiments, n=5-10 was chosen based on the previous publications in the field.
Data exclusions	In qPCR data, samples were rarely excluded when the RNA quality was poor or the expression of the house keeping genes (internal controls) in a particular sample differed from the average of the other samples by more than 2 Ct value.
Replication	Each experiment was independently reproduced at least 2-3 times.
Randomization	The experiments were not randomized.<br>Animals were randomly assigned to different groups, to have the same average body weight in each group.
Blinding	The experiments were not blinded.

Reporting for specific materials, systems and methods

We require information from authors about some types of materials, experimental systems and methods used in many studies. Here, indicate whether each material, system or method listed is relevant to your study. If you are not sure if a list item applies to your research, read the appropriate section before selecting a response.

Materials & experimental systems

n/a	Involved in the study	
☐	☒	Antibodies
☐	☒	Eukaryotic cell lines
☐	☐	Palaeontology
☐	☒	Animals and other organisms
☐	☒	Human research participants
☐	☐	Clinical data

Methods

n/a	Involved in the study	
☐	☐	ChIP-seq
☐	☒	Flow cytometry
☐	☐	MRI-based neuroimaging

Antibodies

Antibodies used	Western Blotting: Anti-UCP1 (ab10983 for detection of mouse UCP1 and ab155117 for detection of human UCP1), Anti-ERRA (ab16363), and Anti-PPARG antibody (ab27649) were purchased from Abcam (Cambridge, MA). Anti-Beta-Tubulin (2146), Anti-FGFR1 (9740), Anti-COX2 (12282), and Anti-FLII (14189) were purchased from Cell Signaling Technologies (Beverly, MA).<br>The following antibodies were used for ChIP experiments: Anti-ERRA (ab16363, Abcam), Anti-H3K27ac (ab4729, Abcam), Anti-H3K4me3 (07-473, Millipore), and H3K9me (ab1220, Abcam).<br>FLAG-M2-magnetic beads (M8823, Sigma-Aldrich) was used for Immunoprecipitation of 3xFLAG-dCas9 in enChIP experiment.<br>Anti-mouse Sca-1 (Ly-6A/E, PerCP-Cy5.5 conjugate, clone E13-161.7, BioLegend) was used for staining of BAT-SVF cells in flow cytometry experiment.<br>These are described in the method section of the manuscript.
Validation	Anti-UCP1 (ab10983): validated in Rat brown adipose tissue by Abcam.<br>Anti-human UCP1 (ab155117): validated in Mouse interscapular brown adipose tissue whole cell lysate by Abcam.<br>Anti-ERRA (ab16363): validated in human and mouse tissues by multiple publications found on Abcam product page.<br>Anti-B-Tubulin (2146): validated in Western blot analysis of extracts from CAD and C6 cells by Cell Signaling technology.<br>Anti-FGFR1 (9740): validated in Western blot analysis of extracts from A-204 cells (FGFR1 positive) by Cell Signaling technology.<br>Anti-COX2 (12282): validated in Western blot analysis of extracts from Raw 264.7 cells by Cell Signaling technology.<br>Anti-FLII (14189): validated in Western blot analysis of extracts from various cell lines by Cell Signaling technology.<br>Anti-H3K27ac (ab4729, Abcam): validate in ChIP experiment using HeLa cells by Abcam<br>Anti-H3K4me3 (07-473, Millipore): validate in Western Blotting on HeLa acid extracted nuclear preps (positive) and recombinant Histone H3 (negative) by Millipore. An independent lab has shown that this antibody performs in chromatin immunoprecipitation (ChIP).<br>H3K9me (ab1220, Abcam): validated in ChIP experiments by Abcam and multiple investigators in mouse ES cells, HEK293, U2OS, and other cell lines.

Eukaryotic cell lines

Policy information about cell lines

Cell line source(s)	Immortalized mouse brown fat SVF cell line (Derived from the stromal vascular fraction of mouse brown adipose tissue)<br>Immortalized mouse white fat SVF (Derived from the stromal vascular fraction of mouse subcutaneous white adipose tissue)<br>Immortalized human neck brown fat SVF (Derived from the stromal vascular fraction of human deep neck fat)<br>Immortalized human neck white fat SVF (Derived from the stromal vascular fraction of human subcutaneous neck fat)<br>MEF (Mouse Embryonic Fibroblast) cell line was derived from mouse embryos.<br>C2C12 Myoblast cell line was purchased from ATCC<br>C3H/10T1/2 mouse multipotent mesenchymal progenitor cell line was purchased from ATCC
Authentication	Immortalized human neck brown fat SVF, C2C12, and C3H/10T1/2 cell lines have been authenticated (ATCC).
Mycoplasma contamination	All the cell lines were tested negative for mycoplasma contamination.
Commonly misidentified lines (See ICLAC register)	No commonly misidentified cell line was used.

Palaeontology

Specimen provenance	*Provide provenance information for specimens and describe permits that were obtained for the work (including the name of the issuing authority, the date of issue, and any identifying information).*
Specimen deposition	*Indicate where the specimens have been deposited to permit free access by other researchers.*
Dating methods	*If new dates are provided, describe how they were obtained (e.g. collection, storage, sample pretreatment and measurement), where they were obtained (i.e. lab name), the calibration program and the protocol for quality assurance OR state that no new dates are provided.*

☐ Tick this box to confirm that the raw and calibrated dates are available in the paper or in Supplementary Information.

Animals and other organisms

Policy information about studies involving animals; ARRIVE guidelines recommended for reporting animal research

Laboratory animals	Mus musculus (C57BL6J, Rosa26-Cas9-adiponectin cre:B6;12;FVB, Ucp1-cre Rosa26-Luciferase: B6:FVB, Ucp1-cre Rosa26-mTmG: B6:129:FVB), male and female, age 8-16 weeks was used for the experiments.
Wild animals	study did not include wild animals.
Field-collected samples	Study did not involve samples collected from the field.
Ethics oversight	All animal procedures were approved by the Institutional Animal Use and Care Committee at Joslin Diabetes Center.

Note that full information on the approval of the study protocol must also be provided in the manuscript.

Human research participants

Policy information about studies involving human research participants

Population characteristics	Described in the methods.
Recruitment	Described in the methods.
Ethics oversight	Described in the methods.

Note that full information on the approval of the study protocol must also be provided in the manuscript.

Clinical data

Policy information about clinical studies

All manuscripts should comply with the ICMJEguidelines for publication of clinical research and a completedCONSORT checklist must be included with all submissions.

Clinical trial registration	*Provide the trial registration number from ClinicalTrials.gov or an equivalent agency.*
Study protocol	*Note where the full trial protocol can be accessed OR if not available, explain why.*
Data collection	*Describe the settings and locales of data collection, noting the time periods of recruitment and data collection.*

Outcomes | *Describe how you pre-defined primary and secondary outcome measures and how you assessed these measures.*

ChIP-seq

Data deposition

☐ Confirm that both raw and final processed data have been deposited in a public database such as GEO.

☐ Confirm that you have deposited or provided access to graph files (e.g. BED files) for the called peaks.

Data access links<br>*May remain private before publication.*	*For "Initial submission" or "Revised version" documents, provide reviewer access links. For your "Final submission" document, provide a link to the deposited data.*
Files in database submission	*Provide a list of all files available in the database submission.*
Genome browser session<br>(e.g. UCSC)	*Provide a link to an anonymized genome browser session for "Initial submission" and "Revised version" documents only, to enable peer review. Write "no longer applicable" for "Final submission" documents.*

Methodology

Replicates	*Describe the experimental replicates, specifying number, type and replicate agreement.*
Sequencing depth	*Describe the sequencing depth for each experiment, providing the total number of reads, uniquely mapped reads, length of reads and whether they were paired- or single-end.*
Antibodies	*Describe the antibodies used for the ChIP-seq experiments; as applicable, provide supplier name, catalog number, clone name, and lot number.*
Peak calling parameters	*Specify the command line program and parameters used for read mapping and peak calling, including the ChIP, control and index files used.*
Data quality	*Describe the methods used to ensure data quality in full detail, including how many peaks are at FDR 5% and above 5-fold enrichment.*
Software	*Describe the software used to collect and analyze the ChIP-seq data. For custom code that has been deposited into a community repository, provide accession details.*

Flow Cytometry

Plots

Confirm that:

☒ The axis labels state the marker and fluorochrome used (e.g. CD4-FITC).

☒ The axis scales are clearly visible. Include numbers along axes only for bottom left plot of group (a 'group' is an analysis of identical markers).

☒ All plots are contour plots with outliers or pseudocolor plots.

☒ A numerical value for number of cells or percentage (with statistics) is provided.

Methodology

Sample preparation	Stromal-vascular fraction was isolated form BAT of Ucp1-Cre Rosa26-mTmG or Ucp1-CreERT2 Rosa26-mTmG mice. Interscapular BAT was dissected, minced, and digested with type 1 Collagenase 1.5 mg/mL; Worthington Biochemical) in Hanks' balanced salt's solution (HBSS, Lonza) containing 2% fatty acid free bovine serum albumin (FF-BSA; Gemini Bio-products, West Sacramento, CA) for 45 minutes at 37 C with gentle shaking. Dissociated tissue was centrifuged at 300 g for 10 minutes. After removing the adipocyte layer and supernatant, SVF pellet was washed with 10% FBS in DMEM, filtered through a 100 um cell strainer, and centrifuged at 300 g for 7 minutes. Cells were incubated in red blood cell lysis buffer (ACK Lysing Buffer, Lonza) for 5 minutes at 4 C. ACK was then diluted by adding 10% FBS in DMEM. The cells were then filtered through a 40 um cell strainer, centrifuged at 300 g for 5 minutes, and resuspended in 1% BSA in PBS. In experiment involving Sca-1 staining, samples were stained with anti-mouse Sca-1 (Ly-6A/E, PerCP-Cy5.5 conjugate, clone E13-161.7, BioLegend) at 1:200 dilution for 30 minutes, followed by washing in cell staining buffer (cat # 420201, BioLegened)
Instrument	BD FACSAria (Becton Dickinson)
Software	Data were collected using DIVA (Becton Dickinson) software and analyzed using FlowJo software (Tree Star, Inc.).
Cell population abundance	No post-sorting analysis was done.

Gating strategy	Debris and dead cells were excluded by forward and side scatter gating. Gating for GFP positive and negative populations was selected using Ucp1 cre;+/+ (no label) and Rosa26-mTmG (fl/fl) (tdTomato label only) control samples (Extended Data Figure 15).

- [x] Tick this box to confirm that a figure exemplifying the gating strategy is provided in the Supplementary Information.

Magnetic resonance imaging

Experimental design

Design type	*Indicate task or resting state; event-related or block design.*
Design specifications	*Specify the number of blocks, trials or experimental units per session and/or subject, and specify the length of each trial or block (if trials are blocked) and interval between trials.*
Behavioral performance measures	*State number and/or type of variables recorded (e.g. correct button press, response time) and what statistics were used to establish that the subjects were performing the task as expected (e.g. mean, range, and/or standard deviation across subjects).*

Acquisition

Imaging type(s)	*Specify: functional, structural, diffusion, perfusion.*
Field strength	*Specify in Tesla*
Sequence & imaging parameters	*Specify the pulse sequence type (gradient echo, spin echo, etc.), imaging type (EPI, spiral, etc.), field of view, matrix size, slice thickness, orientation and TE/TR/flip angle.*
Area of acquisition	*State whether a whole brain scan was used OR define the area of acquisition, describing how the region was determined.*

Diffusion MRI ☐ Used ☐ Not used

Preprocessing

Preprocessing software	*Provide detail on software version and revision number and on specific parameters (model/functions, brain extraction, segmentation, smoothing kernel size, etc.).*
Normalization	*If data were normalized/standardized, describe the approach(es): specify linear or non-linear and define image types used for transformation OR indicate that data were not normalized and explain rationale for lack of normalization.*
Normalization template	*Describe the template used for normalization/transformation, specifying subject space or group standardized space (e.g. original Talairach, MNI305, ICBM152) OR indicate that the data were not normalized.*
Noise and artifact removal	*Describe your procedure(s) for artifact and structured noise removal, specifying motion parameters, tissue signals and physiological signals (heart rate, respiration).*
Volume censoring	*Define your software and/or method and criteria for volume censoring, and state the extent of such censoring.*

Statistical modeling & inference

Model type and settings	*Specify type (mass univariate, multivariate, RSA, predictive, etc.) and describe essential details of the model at the first and second levels (e.g. fixed, random or mixed effects; drift or auto-correlation).*
Effect(s) tested	*Define precise effect in terms of the task or stimulus conditions instead of psychological concepts and indicate whether ANOVA or factorial designs were used.*

Specify type of analysis: ☐ Whole brain ☐ ROI-based ☐ Both

Statistic type for inference (See Eklund et al. 2016)	*Specify voxel-wise or cluster-wise and report all relevant parameters for cluster-wise methods.*
Correction	*Describe the type of correction and how it is obtained for multiple comparisons (e.g. FWE, FDR, permutation or Monte Carlo).*

Models & analysis

n/a	Involved in the study
☐	☐ Functional and/or effective connectivity
☐	☐ Graph analysis
☐	☐ Multivariate modeling or predictive analysis

Functional and/or effective connectivity	*Report the measures of dependence used and the model details (e.g. Pearson correlation, partial correlation, mutual information).*
Graph analysis	*Report the dependent variable and connectivity measure, specifying weighted graph or binarized graph, subject- or group-level, and the global and/or node summaries used (e.g. clustering coefficient, efficiency, etc.).*
Multivariate modeling and predictive analysis	*Specify independent variables, features extraction and dimension reduction, model, training and evaluation metrics.*
